# Abasic pivot substitution harnesses target specificity of RNA interference

**DOI:** 10.1038/ncomms10154

**Published:** 2015-12-18

**Authors:** Hye-Sook Lee, Heeyoung Seok, Dong Ha Lee, Juyoung Ham, Wooje Lee, Emilia Moonkyung Youm, Jin Seon Yoo, Yong-Seung Lee, Eun-Sook Jang, Sung Wook Chi

**Affiliations:** 1 Division of Life Sciences, College of Life Sciences and Biotechnology, Korea University, Seoul 136-701, Korea; 2 Department of Health Sciences and Technology, Samsung Advanced Institute for Health Sciences and Technology, Sungkyunkwan University, Seoul 135-710, Korea; 3 EncodeGEN Co. Ltd., Seoul 135-994, Korea; 4 Samsung Research Institute for Future Medicine, Samsung Medical Center, Seoul 135-710, Korea

## Abstract

Gene silencing via RNA interference inadvertently represses hundreds of off-target transcripts. Because small interfering RNAs (siRNAs) can function as microRNAs, avoiding miRNA-like off-target repression is a major challenge. Functional miRNA–target interactions are known to pre-require transitional nucleation, base pairs from position 2 to the pivot (position 6). Here, by substituting nucleotide in pivot with abasic spacers, which prevent base pairing and alleviate steric hindrance, we eliminate miRNA-like off-target repression while preserving on-target activity at ∼80–100%. Specifically, miR-124 containing dSpacer pivot substitution (6pi) loses seed-mediated transcriptome-wide target interactions, repression activity and biological function, whereas other conventional modifications are ineffective. Application of 6pi allows PCSK9 siRNA to efficiently lower plasma cholesterol concentration *in vivo*, and abolish potentially deleterious off-target phenotypes. The smallest spacer, C3, also shows the same improvement in target specificity. Abasic pivot substitution serves as a general means to harness the specificity of siRNA experiments and therapeutic applications.

## Abstract (web summary)

RNA interference inadvertently represses off-target transcripts. Here, Lee *et al.* report that substituting nucleotide in position 6 of the seed region of the small interfering RNAs with abasic spacers can significantly decrease miRNA-like off-target repression while preserving on-target activity.

## Graphical abstract

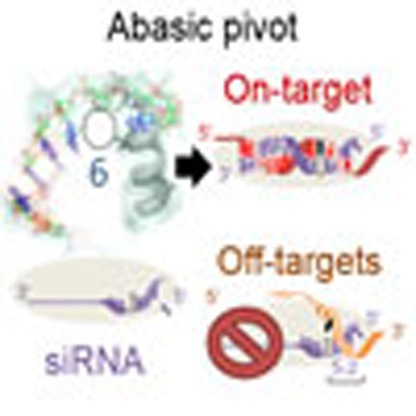

The RNA-induced silencing complex (RISC) is responsible for inducing gene silencing caused by RNA interference (RNAi), guided by ∼21 to 23 nucleotides (nt) duplexes of RNAs in a sequence-dependent manner^1^. As regulators of biological function, microRNAs (miRNAs) are endogenously produced to form such structures, of which one strand termed ‘guide strand' directs the RISC to post-transcriptionally repress target genes by altering mRNA stability and/or translation^2^,^3^. Functional miRNA–target interactions generally require partial sequence complementarity, majorly pairing as few as 6 nt matches within the seed region (positions 2–8; ref. 4). To utilize the RISC to selectively silence a desired target gene, small interfering RNAs (siRNAs) are typically designed and synthesized as duplexes, in which the guide strand is perfectly complementary to the target mRNA. When siRNAs are introduced into the cell^5^, the guide strand triggers cleavage of the intended target mRNA by loading into Argonaute (Ago2, also known as Eif2c2), the core catalytic component of the RISC^6^. However, a critical caveat in using siRNAs is that any ∼21 to 23 nt RNA incorporated into Argonaute (Ago) can also function as miRNA, by the mechanism via which miRNAs recognize target mRNAs^7^,^8^. Thus, the use of siRNAs always results in the repression of hundreds of off-target transcripts, thereby potentially leading to unintended phenotypes.

A number of studies have demonstrated that the off-target effects of siRNAs are widespread throughout transcripts^8^,^9^ and seed-centric in terms of recognition^10^,^11^ as shown with miRNAs^4^. Compared with cleavage-mediated on-target silencing, off-target activity is relatively modest at an individual transcript level^9^,^10^, and often considered phenotypically irrelevant^8^. Nevertheless at the global level of transcripts, off-target activity can affect biological functions and phenotypes. Cell death^12^ and inhibition of cell growth^10^ have been reported as a direct consequence of off-target repression^13^. miRNA-like activity also yields up to ∼30% of false-positive hits during siRNA screening in functional genetic studies, often causing more dramatic changes in the phenotype than on-target repression^14^,^15^. Several approaches have been proposed to reduce off-target effects in siRNA experiments^16^, such as the use of appropriate controls^15^, low siRNA concentrations^17^, multiple siRNAs targeting the same gene individually or as a pool^18^ and bioinformatics approaches^19^. Nevertheless, such indirect methods do not definitely tackle off-target effects, for which an absolute solution is especially necessary in the clinical application^20^. Several chemically modified nucleotides such as 2′-*O*-methyl nucleotide at position 2 (2′-OMe)^13^ and unlocked nucleic acid at position 7 (UNA)^21^ have been introduced to attenuate off-target repression, although the on-target activity was also reduced^13^,^21^,^22^. However, no approach is currently available to eliminate the miRNA-like off-target effects.

To control off-target effects, it is important to understand how the Ago–miRNA complex recognizes targets. The most well characterized features of miRNA–target interactions are seed matches, short base-pairing at least 6 nt long with seeds (within positions 2–8; ref. 4). Seed matches are informative for prediction and identification of miRNA-like off-target sites^10^,^11^. Such seed-mediated interactions are well supported by recent structural studies of human Ago–miRNA^23^,^24^ and Ago–miRNA–target complex^25^, demonstrating that nucleotides in positions 2–6 are prearranged in an A-form helical structure that makes them susceptible to base pairing (Fig. 1a). Geometrical disruption between position 6 and 7 occurs in miRNA because of the insertion of an amino acid, isoleucine (I365) from human Ago2 (refs 23, 24). Stepwise processes, in which nucleotides in positions 2–5 are initially exposed and paired for conformational changes to expose nucleotides 2 to 8, are required to overcome the kink in target recognition^25^.

In support of this implication, a requirement of consecutive base pairs in positions 2–6 (termed ‘nucleation') was elucidated by genome-wide biochemical analysis of *in vivo* miRNA–target interactions^26^. This method applies cross-linking immunoprecipitation of the RNA–protein complex (CLIP)^27^ coupled with high-throughput sequencing (HITS)^28^ to Ago (Ago HITS–CLIP)^29^. Ago HITS–CLIP analyses that were performed in the mouse brain initially identified substantial numbers of non-canonical miRNA target sites called ‘nucleation bulges', which form a bulge in target mRNAs between position 5 and 6 of the corresponding miRNA^26^. This was further identified as a general rule governing nucleation bulges, ‘pivot pairing rule'. This rule determines nucleotide composition in the bulge position, postulating that nucleotide in a bulge should be able to pair with a nucleotide in position 6 (named ‘pivot', Fig. 1b)^26^,^30^. Implicated together as the ‘transitional nucleation model', nucleation bulges should transiently form consecutive base pairs up to the pivot (transitional nucleation). This is a prerequisite for initiation and propagation of base-pairing toward the 3' end for functional miRNA–target interactions, where the nucleotide originally matching the pivot in the target RNA becomes bulged-out^26^,^31^. Nucleation bulge sites have been observed in the mouse neocortex^29^, human brain^32^ and cell lines^26^,^33^, also by using a ligation based Ago-CLIP method, CLASH^34^.

Consistent with the structural observation^23^,^24^,^25^, transitional nucleation may serve as a general mechanism for initiating miRNA-like target recognition^26^. This notion indicates that pairing in the pivot (position 6) serves as a decisive border to form functional miRNA–target interactions. To avoid the initiation step of miRNA-like off-target recognition, we impaired the pivot in siRNAs by substituting it with a spacer that contains no base. Such abasic pivot substitution is generated by using dSpacer (abasic deoxynucleotide; 6pi) or C3 spacer (three-carbon spacer; 6c3). Abasic pivot substitution eliminates seed-mediated off-target repression while preserving superior on-target activity (∼80–100% of maximal silencing activity, relative to the siRNA without a spacer). This provides a general means for harnessing the specificity of RNAi, thus preventing potential misinterpretations of gene silencing studies or adverse effects for therapeutic applications.

## Results

### miRNA-like off-target repression of siRNA is widespread

**Figure 1:**
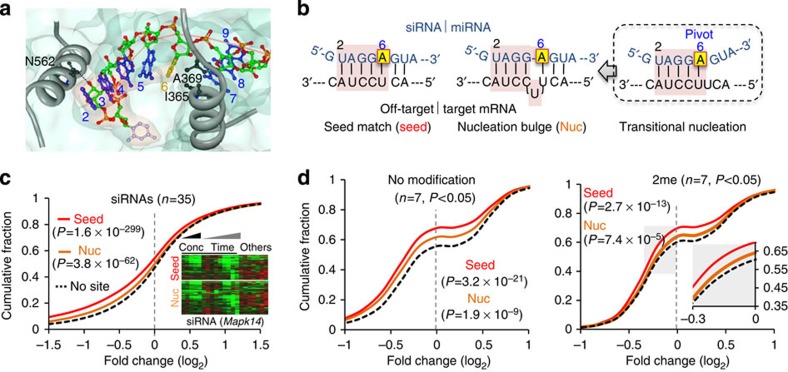
Widespread nucleation bulge sites in siRNA off-targets. (**a**) Structure of the human Ago–miRNA complex^24^; bases in blue; α-helices of Ago in grey (with indication of I365 and A369); the surface of miR-20a in faint red and Ago in faint green; the possible hydrogen bond between N562 and 2′-OH at nucleotide position 2 is indicated with a dashed-line, and may be abrogated by 2′-OMe. (**b**) miRNA-like off-targeting of siRNA (for example, an siRNA targeting *Renilla* luciferase; siRL) caused by seed matches (Seed, left panel) or nucleation bulge sites (Nuc, middle panel) through transitional nucleation (base-pairs from position 2 to 6, red shade, right panel)^26^; pivot (position 6) in yellow box; siRL in blue; off-target mRNA in black. (**c**) Meta-analysis of putative siRNA off-target transcripts that contain miRNA-like target sites (Seed or Nuc) in 3′-UTR. On the basis of compiled microarray data from expression of 35 different siRNAs^35^, cumulative fraction of transcripts depending on fold changes (log_2_ ratio, relative to control) was analysed (left panel) and compared with control transcripts (No site; transcripts with neither Seed nor Nuc); *P* values from KS-test. Heatmap analysis of putative off-target transcripts, which were clustered depending on fold changes at different concentrations (Cons), times (Time) and sequences (Others) of siRNAs targeting Mapk14 (ref. 9; right panel). (**d**) The same analysis as in left panel of **c** except only for transcripts with significant fold changes (*P*<0.05) depending on the expression of seven different siRNAs^13^; no modification (left panel) versus 2me (2′-OMe in position 2, right panel). Of note, siRNAs with 2′-OMe still showed off-target repression especially where there was marginal fold repression (right panel, grey inset). The highest *P* value (2me, Nuc, *P*=7.4 × 10^−5^, KS-test) among all the samples (except for the negative control) was still statistically significant even after Bonferroni correction was applied (*n*=7, *P*=1.9 × 10^−4^).

To confirm the observation of widespread miRNA-like off-target repression^8^,^9^ possibly mediated through transitional nucleation in siRNAs (Fig. 1b), we first performed transcriptome-wide analysis in compiled transcript profiles^35^ from 35 different siRNAs (Supplementary Table 1a). In analyses of cumulative distributions of siRNA-dependent repression, the transcripts containing nucleation bulges showed a propensity for downregulation relative to the distribution of transcripts without seed matches or nucleation bulges (‘No site' in Fig. 1c, left panel, and Supplementary Fig. 1a). Although nucleation bulge sites had less effect than seed sites, such downregulation was significant at sites in 3′-untranslated regions (3′-UTRs, *P*=3.8 × 10^−62^, Kolmogorov–Smirnov test (KS-test), relative to ‘No site', Fig. 1c, left panel), or at any location in the transcripts (*P*=2.4 × 10^−51^, KS-test, Supplementary Fig. 1a). Analysis of a subset of the compiled data^35^ focusing on siRNAs targeting a specific gene (mitogen-activated protein kinase 14; *Mapk14*, Supplementary Table 1b) indicated that such off-target effects mediated by nucleation bulges are concentration-, time- and sequence-dependent as evident in seed sites (Fig. 1c, right panel). These data suggest that off-target repression is only mediated via specific sequence of siRNA and not a consequence of the target gene repression.

Transcript profiles of siRNAs with 2′-OMe were then examined (seven different siRNAs, Supplementary Table 2) to determine whether widespread off-target repression can be effectively prevented^13^. A cumulative analysis of the putative off-target transcripts showed significant siRNA-dependent repression (Fig. 1d, *P*<0.05)^13^, providing evidence that siRNAs with 2′-OMe still caused significant off-target repression (Fig. 1d, right panel) not only for seed sites (*P*=2.7 × 10^−13^, KS-test), but also for nucleation bulge sites (*P*=7.4 × 10^−5^, KS-test). However, some off-target repression was reduced by 2′-OMe relative to unmodified siRNAs (Fig. 1d and Supplementary Fig. 1b, c). Nucleation bulges and seed sites are widespread in siRNA off-targets, and such miRNA-like off-target repression is not eliminated completely by the conventional 2′-OMe modification.

### Abasic substitution within nucleation region of siRNAs

**Figure 2:**
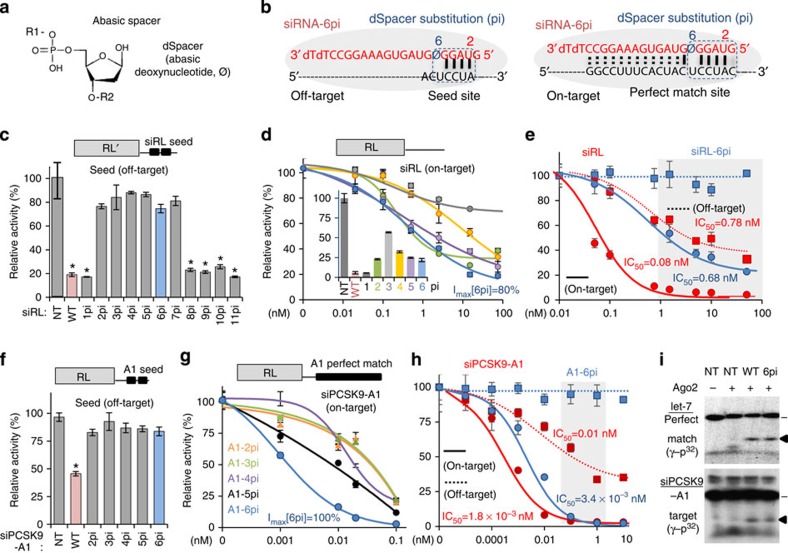
Effect of abasic spacer substitution for a nucleotide in the nucleation region of siRNA. (**a**) Abasic deoxynucleotide (dSpacer, φ), applied to a nucleotide in siRNA as abasic spacer substitution. (**b**) dSpacer substitution (pi) in the nucleation region causes a single mismatch to seed sites in off-target transcripts (for example, dSpacer pivot substitution, 6pi), leading to unstable transitional nucleation (for example, siRL-6pi). However, siRNA-6pi may induce a stable interaction only for on-target with a perfect match site through compensatory near-perfect matches (right panel). Details are in Supplementary Fig. 2. Of note, the nomenclature ‘pi' is derived from ‘φ' which here stands for abasic spacer substitution with a deoxynucleotide linker, dSpacer. (**c**) Luciferase reporter assays for miRNA-like off-target repression, mediated by seed sites for siRL (75 nM) with pi. Relative activity (average *Renilla* luciferase activity normalized to firefly luciferase) was analysed as a percentage relative to the control ('NT', non-targeting control siRNA); error bars, s.d. WT indicates the unmodified siRNA (red bar). Asterisk denotes *P*<0.01 (*t*-test, *n*=6). RL′ indicates a different sequence of *Renilla* luciferase gene, that could not be targeted by siRL. (**d**) The same luciferase assay as in **c** except for measuring on-target repression (inner set). Repression efficiency was measured at different concentrations of siRL with pi (outer set; 2–6pi, indicated by different colours). IC_50_ and *I*_max_ values are represented in Supplementary Fig. 3c. (**e**) On-target activity (solid line) was examined together with off-target activity (dotted line) for siRL (red) versus siRL-6pi (blue) by luciferase reporter assays. General siRNA concentrations used for cell culture are indicated (grey, 1–100 nM). (**f**–**g**) Effects of dSpacer substitution (pi) in the nucleation region were also examined for siPCSK9-A1 (ref. 36) in **f** as in **c** and in **g** as in **d**. (**h**) The same analysis as in **e** except for siPCSK9-A1; the grey colour indicates the therapeutic concentration. (**i**) *In vitro* Ago2 cleavage assays for let-7 (upper panel) and siPCSK9-A1 (lower panel). The triangle denotes expected size of cleaved product from the target substrate (indicated with a line).

Widespread nucleation bulges in off-target repression (Fig. 1) suggest that destabilizing transitional nucleation (base pairs in position 2–6) might completely block the initiation of off-target recognition. The 2′-OMe possesses intrinsic limitations in blocking off-target repression because it is introduced into the nucleotide backbone rather than into the base. For this reason, we used an abasic spacer, which contains no base but functions as a linker (for example, abasic deoxynucleotide spacer; dSpacer, φ, Fig. 2a) as a substitute (dSpacer substitution; pi) for a nucleotide in the nucleation region (position 2–6). We hypothesized that such abasic substitution (for example, dSpacer substitution for position 6, 6pi; Fig. 2b and Supplementary Fig. 2) could effectively prevent miRNA-like off-target repression (left panel, Fig. 2b) and conserve on-target activity (right panel, Fig. 2b).

The potential for miRNA-like off-target repression was confirmed for siRNA that targets *Renilla* luciferase (siRL), showing significant off-target repression either through seed sites (81% repression, relative to non-targeting control (NT), *P*<0.01, *t*-test, Fig. 2c) or nucleation bulge sites (34% repression, *P*<0.01, *t*-test, Supplementary Fig. 3a) in luciferase reporter assays. Of note, siRL is often used as a negative control in siRNA experiments. A nucleotide in the nucleation region was then substituted with dSpacer (pi) to maintain the nucleotide structure without a base. Regardless of the position, all siRLs containing dSpacer substitution (pi) in the nucleation region (2pi, 3pi, 4pi, 5pi or 6pi) or position 7 (7pi) showed derepression for seed sites (Fig. 2c and Supplementary Fig. 3a).

The dSpacer substitution (pi) may also affect the efficiency of siRNA on-target activity. Alternatively, a single mismatch generated by an abasic spacer could be tolerated by compensatory near-perfect matches (for example, dSpacer pivot substitution: 6pi; Fig. 2b, right panel). The efficiency of on-target repression was estimated by measuring half maximal inhibitory concentration (IC_50_) through luciferase reporter assays (Fig. 2d and Supplementary Fig. 3b,c). Every siRL containing dSpacer in the nucleation region (position 2–6) showed some repression activity (maximal inhibition rate (*I*_max_)=43–80%, relative to the unmodified (*I*_max_[WT]=100%)). Among them dSpacer pivot substitution (6pi) yielded the best efficiency of on-target repression (*I*_max_[6pi]=80%, IC_50_[6pi]=0.36 versus IC_*50*_[WT]=0.04 nM, Fig. 2c,d and Supplementary Fig. 3b,c). Intriguingly, significant repression of seed-containing off-targets (*I*_max_=81%, IC_50_=0.78 nM, Fig. 2e), which was observed in every concentration range generally used for siRNA transfection into cell cultures (1–100 nM; 50–81% repression, *P*<0.01, *t*-test), was completely eliminated by the dSpacer pivot substitution (IC_*50*_[6pi]=ND (not determined), 0% repression), although ∼9-fold decrease in the efficacy of on-target repression was observed with siRL-6pi (IC_50_[6pi]=0.68 versus IC_50_[WT]=0.08 nM, Fig. 2e).

By expanding the application to another siRNA (siPCSK9-A1)^36^ that targets proprotein convertase subtilisin/kexin type 9 (PCSK9), seed-mediated off-target repression (54% repression, *P*<0.01, *t*-test, Fig. 2f) was observed to be disappeared when dSpacer substitution (pi) was applied to the pivot (6pi) or other positions in the nucleation region (Fig. 2f; IC_50_[WT]=0.01 nM versus IC_50_[6pi]=ND, Supplementary Fig. 3d). siPCSK9-A1 was previously developed to lower low-density lipoprotein cholesterol in plasma by inhibiting PCSK9-mediated degradation of the low-density lipoprotein receptor in the liver^36^. The same positional effect of dSpacer substitution on the on-target activity of siPCSK9-A1 was confirmed by observing that the dSpacer pivot substitution (6pi) outperformed others (*I*_max_[6pi]=100%, Fig. 2g and Supplementary Fig. 3e). siPCSK9-A1-6pi showed superior conservation of on-target activity (95–100% repression) without any seed-mediated off-target repression (IC_50_[6pi]=ND versus IC_50_[WT]=0.01 nM, Fig. 2h). This effect was especially consistent in the concentration range appropriate for therapeutic use of siRNA (0.1–1.0 nM, shaded in grey colour, Fig. 2h), although overall ∼2-fold decrease in the efficacy of on-target activity was observed (IC_50_[6pi]=3.4 × 10^−3^ versus IC_50_[WT]=1.8 × 10^−3^ nM, Fig. 2g,h and Supplementary Fig. 3e). Of note, significant seed-mediated off-target repression was also observed at a low concentration of siPCSK9-A1 (25% repression at 0.0001, nM, *P*<0.01, *t*-test, Fig. 2h). Furthermore, dSpacer pivot substitution (6pi) did not alter slicing activity on perfectly matched on-target site (Fig. 2i), confirmed by *in vitro* Ago2 cleavage assay for let-7 (upper panel) and siPCSK9-A1 (lower panel). Therefore, the most favourable position for abasic substitution in siRNAs is the pivot (position 6) because this abolishes seed-mediated off-target repression while maintaining on-target activity.

### dSpacer pivot substitution outperforms in target specificity

**Figure 3:**
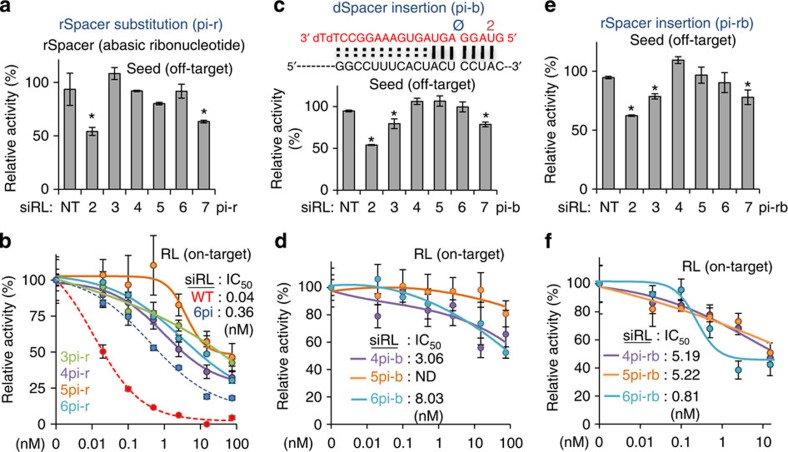
Comparison of various abasic spacer conformations within the nucleation region. (**a**) A positional effect of rSpacer (abasic ribonucleotide) substitution (pi-r), when applied to a nucleotide within the nucleation region, was examined for seed-mediated off-target repression as in Fig. 2c. siRLs harbouring pi-r within positions 3–6 showed significant derepression. (**b**) Efficiency of on-target activity of siRL with pi-r (within position 3–6) was examined as in Fig. 2d to compare the results with unmodified siRL (WT, IC_50_=0.04 nM, red dashed line, as measured in parallel with Supplementary Fig. 3b) and siRL-6pi (IC_50_=0.62 nM, blue dashed line, as measured in parallel with Fig. 2d). Details on the IC_50_ values are provided in Supplementary Fig. 4c. (**c**) Insertion of dSpacer (pi-b) into the nucleation region of siRL that results in bulge formation when siRL anneals to an on-target site (upper panel): examination for seed-mediated off-target repression by luciferase reporter assays as in **a** (lower panel). siRLs harbouring pi-b within positions 4–6 showed significant derepression. Of note, the nucleation bulge can be caused by the Ago–miRNA structure because the bulge occurs in target RNA, where there is no significant contact with Ago^23^,^24^,^25^. (**d**) The same assay as in **b** except siRL contained pi-b within positions 4–6; ND indicates that IC_50_ could not be determined. (**e**) The effect of inserting an rSpacer (pi-rb) into the nucleation region of siRL on seed-mediated off-target repression, when measured as in **a**. (**f**) The same experiment as in **d** except for pi-rb. Of note, every spacer conformation that showed derepression of seed-mediated off-targets had limited potency to induce on-target silencing, in comparison with the superior on-target activity of 6pi. Asterisk denotes *P*<0.01 (*t*-test, *n*=6).

We also investigated the possibility that other conformations of the abasic spacer could outperform the dSpacer pivot substitution (6pi). First, the effect of rSpacer (abasic ribonucleotide) substitution (pi-r) in the nucleation region of siRL was examined (Fig. 3a,b and Supplementary Fig. 4). Abolition of the seed-mediated off-target repression was observed when the rSpacer substitution was applied to a nucleotide in positions 3–6 (Fig. 3a). Nevertheless, all such siRLs containing the rSpacer substitution had less efficient on-target activity (*I*_max_≤70%, IC_50_≥0.99 nM) than dSpacer pivot substitution (6pi, *I*_max_=80%, IC_50_=0.36 nM; Fig. 3b and Supplementary Fig. 4). Next, the insertion of dSpacer (pi-b) into the nucleation region was noted to cause a bulge with alignment to the target site (Fig. 3c, upper panel). The seed-mediated off-targets were found to be derepressed when dSpacer insertion was within positions 4–6 (Fig. 3c, lower panel). Although some on-target activity was retained (*I*_max_≤48%, IC_50_≥3.06 nM), all of these insertions showed less efficient on-target activity than dSpacer pivot substitution (Fig. 3d and Supplementary Fig. 5). Similarly, after insertion of rSpacer (pi-rb), abrogation of off-target repression was observed (positions 4–6, Fig. 3e) with insufficient preservation of on-target activity (*I*_max_≤55%, IC_50_≥0.81 nM) in comparison with the dSpacer pivot substitution (Fig. 3f and Supplementary Fig. 6). All of these data demonstrate that although off-target repression can be abolished by other spacer conformations, which used rSpacer or caused an abasic bulge of siRNA in siRNA:target duplex, these conformations cannot maintain on-target activity as well as dSpacer pivot substitution.

### Abasic pivot substitution eliminates off-target repression

**Figure 4:**
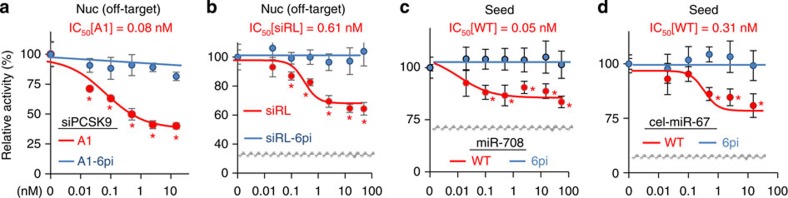
Abasic pivot substitution abolishes seed-mediated miRNA-like target repression. (**a**) miRNA-like off-target activity of siPCSK9-A1, mediated by nucleation (Nuc) bulge sites, was examined through luciferase reporter assays in the presence and absence (WT) of 6pi, as in Fig. 2h (*n*=6); error bars, s.d. Asterisk denotes *P*<0.01 (*t*-test). (**b**) The same experiment as in **a**, except for siRL. (**c**–**d**) Efficiency of seed-mediated target repression by miRNAs was measured in the presence of 6pi, as in **a**. Elimination of seed-mediated target repression is shown for (**c**) miR-708-6pi and (**d**) cel-miR-67-6pi. Of note, because cel-miR-67 is expressed only in *C.elegans*, it has been widely used as a control in miRNA or siRNA experiments. Nonetheless, cel-miR-67 showed miRNA-like off-target repression. IC_50_ values of all small RNAs containing 6pi could not be determined (IC_50_=ND) because there was no significant repression in every range of siRNA concentration (0.05–75 nM). Breaks in *y* axis were indicated in grey. ND, not determined.

To further confirm whether abasic pivot substitution can abolish miRNA-like off-target recognition, we assessed seed-mediated off-target activity for nucleation bulge sites of PCSK9-A1 and siRL (Fig. 4a,b). Significant off-target repression mediated by nucleation bulge sites for PCSK9-A1 (IC_50_=0.08 nM, 63% repression, *P*<0.01, *t*-test, Fig. 4a) and siRL (IC_50_=0.61 nM, 38% repression, *P*<0.01, *t*-test, Fig. 4b) was abolished by dSpacer pivot substitution (6pi, IC_50_=ND, 0% repression, Fig. 4a,b). In addition, siRL-6pi showed inhibition of passenger strand-mediated off-target activity (Supplementary Fig. 7). The use of dSpacer pivot substitution was then tested on miRNAs with the expectation of abrogating seed-mediated target repression (Fig. 4c,d). Synthesized miRNA duplexes containing the dSpacer pivot substitution showed derepression of the target with seed sites (IC_50_=ND, 0% repression) for miR-708 (IC_50_=0.05 nM, 20% repression, Fig. 4c) and cel-miR-67 (IC_50_=0.31 nM, 23% repression, Fig. 4d), the *C.elegans*-specific miRNA often used as a control. Notably, in all ranges of concentration tested here, there was no significant change in relative activity of luciferase reporters for every siRNA and miRNA with dSpacer pivot substitution (IC_50_[6pi]=ND, 0% repression, Figs 3 and 4). All of these data provide evidence that the dSpacer pivot substitution is generally applicable to any siRNA for eliminating miRNA-like off-target repression.

### Abasic pivot substitution conserves on-target activity

**Figure 5:**
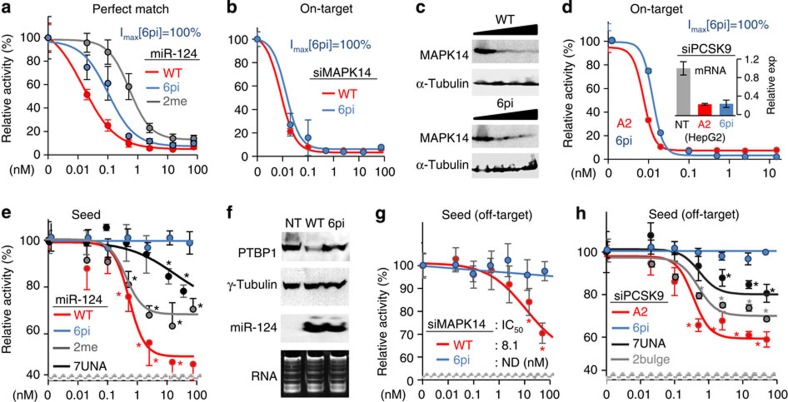
Abasic pivot substitution improves target specificity of siRNA compared with other modifications. (**a**) In the absence (WT) and presence of dSpacer pivot substitution (6pi) or 2′-OMe (2me, in position 2), luciferase reporter assays with a perfectly matched site for miR-124 were performed as in Fig. 2e (IC_50_[WT]=0.05, IC_50_[6pi]=0.10, IC_50_[2me]=0.53 nM). (**b**) The same analysis as in **a** except for siMAPK14 (IC_50_[6pi]=0.021 versus IC_50_[WT]=0.017 nM). (**c**) Immunoblot analysis of MAPK14 at different concentrations of siRNA transfection (0, 0.5, 5 or 50 nM) relative to control (α-tubulin). (**d**) Efficiency of on-target activity was estimated as in **a**, except for siPCSK9-A2 (IC_50_[6pi]=0.013 versus IC_50_[WT]=0.008 nM, left panel). Of note, IC_50_ for siMAPK14 (**b**) or siPCSK-A2 (**d**) was approximately estimated owing to its strong repressive activity. siPCSK9-A2 has the same nucleotide sequence as siPCSK9-A1 except it contains 2′-OMe in several positions other than the seed regions to avoid innate immune responses^36^. *PCSK9* mRNA levels in HepG2 cells were measured by qPCR after transfection of siPCSK9-A2 (50 nM, relative expression to control (NT) transfection, normalized to *GAPDH*, right panel). (**e**) Effect of various modifications (6pi, 2me and 7UNA; UNA modification at position 7) on seed-mediated target repression was measured for miR-124 (IC_50_[WT]=0.07, IC_50_[2me]=0.65, and IC_50_[7UNA]=7.2 nM) as performed in Fig. 4. (**f**) miR-124-6pi derepresses a validated miR-124 target, *PTBP1* (ref. 38), confirmed by immunoblot analysis; γ-tubulin served as a control. Transfected miR-124 and miR-124-6pi was confirmed by northern blotting. ‘RNA' indicates staining of total RNA. (**g**) The same experiments as in **b** except for measuring off-target repression mediated by seed sites. (**h**) Seed-mediated off-target activity of siPCSK9-A2 (IC_50_=0.91 nM) was examined in the presence of 7UNA (IC_50_=0.97 nM) or 2bulge (bulge-siRNA^37^, which contains a bulge at position 2 of the guide strand in siRNA duplex; IC_50_=0.96 nM) as in **e**. All small RNAs containing 6pi showed depression of miRNA-like targets mediated by seed regions (IC_50_=ND, 0% repression). ND, not determined. Asterisk denotes *P*<0.01 (*t*-test, *n*=6).

To examine the extent of preserved on-target activity in siRNA-6pi, IC_50_ and *I*_max_ were also measured for a perfectly matched site of miR-124 (Fig. 5a). Luciferase reporter assays showed that miR-124-6pi has the same maximal repression activity as miR-124 (*I*_max_=100%), although application of dSpacer pivot substitution showed slight reduction (∼2-fold decrease, IC_50_[6pi]=0.10 versus IC_50_[WT]=0.05 nM, Fig. 5a). For the siRNA targeting MAPK14 (siMAPK14), near-perfect conservation of the on-target activity was observed (*I*_max_=100%) across all ranges of concentrations used for cell cultures (100% conservation, 0.05–75 nM, Fig. 5b). This was further confirmed by immunoblot analyses (Fig. 5c and Supplementary Fig. 8a,b). When the dSpacer pivot substitution was applied to siPCSK9-A2, which has the same sequence as siPCSK9-A1 but with 2′-OMe modification to increase stability and avoid innate immune responses *in vivo*^36^, the same conservation of on-target activity was observed (*I*_max_=100%, Fig. 5d, left panel). Indeed, both siPCSK9-A2 and siPCSK9-A2-6pi efficiently silenced PCSK9 mRNA by inducing the same degree of repression (∼5-fold, Fig. 5d, right panel). This finding was further confirmed for siPCSK9-A1 by immunoblotting analysis (Supplementary Fig. 8c). Importantly, all of these small RNAs showed abolition of miRNA-like activity when they contained the dSpacer pivot substitution (IC_50_=ND, 0% repression, Fig. 5e–h). Taken together, these results lead to the conclusion that dSpacer pivot substitution maintains superior on-target activity (∼80–100%, relative to the unmodified) while avoiding the off-target repression.

### Incomplete elimination of off-targets by conventional methods

Next, we examined the efficiency of blocking the miRNA-like off-target repression by conventional methods such as 2′-OMe^13^ and UNA^21^. For this purpose, we applied 2′-OMe or UNA to miR-124, which has the most Ago-bound seed sites in the brain^29^,^32^, to see whether its inhibitory effect is strong enough to block the silencing activity of this seed-centric miRNA (seed, IC_50_=0.07 nM, *I*_max_=52%, relative to perfectly matched target, Fig. 5e; nucleation bulge, IC_50_=0.78 nM, *I*_max_=27%, Supplementary Fig. 8d). In the luciferase reporter assay, miR-124 containing 2′-OMe showed significant repression for both seed (IC_50_=0.65 nM, *I*_max_=32%, Fig. 5e and Supplementary Fig. 8d) and nucleation bulge sites (IC_50_=0.91 nM, *I*_max_=19%, Supplementary Fig. 8e). Such incomplete inhibition of seed-mediated miRNA-like activity was also observed for UNA applied to miR-124 (IC_50_=7.2 nM, *I*_max_=21%, Fig. 5e) and siPCSK9-A2 (IC_50_=0.97 nM, *I*_max_=18%, Fig. 5h). Bulge-siRNA, which was developed to alleviate off-target repression by containing a bulge at position 2 of the guide strand in the siRNA duplex^37^, also showed remaining off-target activity when applied to siPCSK9-A2 (IC_50_=0.96 nM, *I*_max_=29%, Fig. 5h). However, the dSpacer pivot substitution showed elimination of seed-mediated off-target repression in all cases (IC_50_=ND, 0% repression, Fig. 5e–h). This finding was further confirmed by immunoblotting of PTBP1, a previously validated miR-124 target with seed sites^38^ (Fig. 5f and Supplementary Fig. 8f–h). Of note, small RNAs containing 2′-OMe have less silencing activity toward the perfectly matched on-target than the dSpacer pivot substitution, as observed in miR-124 (IC_50_[2me]=0.53 versus IC_50_[6pi]=0.10 nM, Fig. 5a) and siRL (IC_50_[2me]=0.63 versus IC_50_[6pi]=0.41 nM, Supplementary Fig. 9a). In addition, the dSpacer pivot substitution slightly outperformed UNA in on-target repression by siPCSK9-A2 (Supplementary Fig. 9b). Overall, the current methods for overcoming off-target silencing have limited potency, whereas dSpacer pivot substitution could eliminate miRNA-like off-target repression with improved potency for preserving on-target repression.

### Global and functional validation of abasic pivot substitution

**Figure 6:**
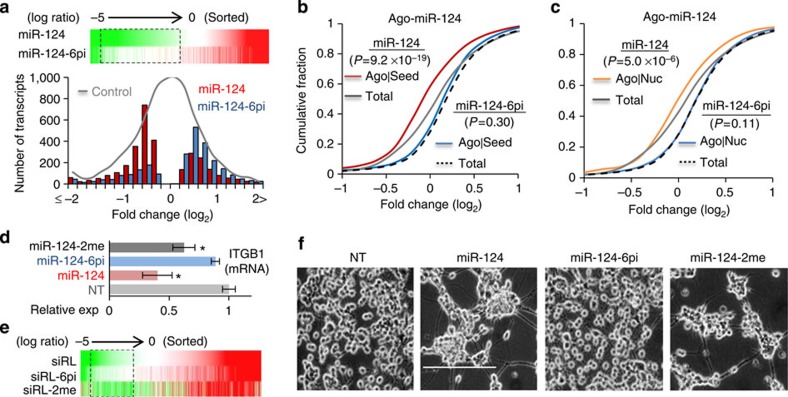
Loss of global target repression and function in miR-124 by abasic pivot substitution. (**a**) Transcriptome-wide derepression of miR-124 by dSpacer pivot substitution is represented as a heat map where transcripts are sorted by fold change (log_2_ ratio) of miR-124-dependent repression (upper panel). Distribution of the transcripts with a significant fold change depending on miR-124 (red bar) or miR-124-6pi expression (blue bar) was plotted together with all transcripts from control (NT, grey line; lower panel). The dotted box (upper panel) indicates a significant derepression range shown by comparing the distribution of fold changes (sorted fold change <0, upper panel; *P*<0.01, binomial test, lower panel). All RNA-Seq data from HeLa cells (NT versus miR-124 transfection), which do not express miR-124. (**b**) Cumulative distribution of transcripts with seed sites in *de novo* Ago–miR-124 clusters (Ago|Seed) were analysed depending on transcriptome profiles under miR-124 (red) and miR-124-6pi expression (blue), together with all transcripts (total) containing *de novo* Ago–miR-124 clusters. miR-124-6pi showed no significant difference in the distribution showing repression (fold change <0) relative to total (*P*=0.30, KS-test), indicating that miR-124-6pi cannot bind and repress target transcripts containing seed sites bound by Ago–miR-124. (**c**) The same analysis as in **b**, except for nucleation bulge sites (Nuc). Cumulative fraction analysis of transcriptome profiles under miR-124-6pi expression for Ago|Nuc showed no significant difference (*P*=0.11, KS-test). (**d**) miR-124-dependent repression of a known endogenous target mRNA (*ITGB1* (ref. 29), normalized to *GAPDH*) was measured by qPCR; 6pi versus 2me, relative to control (NT). Asterisk denotes *P*<0.01 (*t*-test, *n*=3). (**e**) The same analysis as in the upper panel of **a**, except 6pi is compared with 2me in siRL. (**f**) miR-124-induced neurite outgrowth in N2a cells was prevented by 6pi but not by 2me, likely because of remaining seed-mediated repression activity, detected as in **d**. See details in Supplementary Fig. 11. Scale bar indicates 500 μm. Time lapse images are also available (miR-124, Supplementary Movie 1; miR-124-6pi, Supplementary Movie 2; miR-124-2me, Supplementary Movie 3).

We further validate the effect of abasic pivot substitution on transcriptome profiles, initially focusing on miR-124. Analysis of RNA-Seq experiments (Supplementary Table 3a) performed in miR-124 and miR-124-6pi transfected HeLa cells showed that, in contrast to minor changes in upregulated transcripts, downregulated transcripts depending on miR-124 expression were majorly derepressed by miR-124-6pi (Fig. 6a, upper panel). Such derepression (1,293 transcripts) was significant (*P*=5.1 × 10^−5^, KS-test, miR-124 versus miR-124-6pi, Supplementary Fig. 10a) in miR-124 dependent transcripts (Fig. 6a, lower panel) and in all transcripts (*P*=4.3 × 10^−93^, KS-test, Supplementary Fig. 10a). In addition, the same RNA-Seq analyses for siRL-6pi (Supplementary Table 3a) showed the same propensity for derepression (Supplementary Fig. 10b–d).

We also investigated whether miR-124-6pi loses its global binding to direct target sites *in vivo* by using *de novo* Ago–miR-124 clusters, which appeared after transfection of miR-124 into HeLa cells, in the Ago HITS-CLIP analysis^29^. As reported previously in cumulative distribution analysis^29^, the transcripts harbouring *de novo* Ago–miR-124 clusters were significantly downregulated by miR-124 expression (*P*=2.4 × 10^−14^, relative to total, KS-test, Supplementary Fig. 10e) containing either seed sites (*P*=9.2 × 10^−19^, KS-test, Fig. 6b) or nucleation bulge sites (*P*=5.0 × 10^−6^, KS-test, Fig. 6c). Nevertheless, none of the direct miR-124 targets showed significant repression in the presence of miR-124-6pi (*P*=0.25, KS-test, Supplementary Fig. 10e), suggesting that there was no functional direct interaction between miR-124-6pi and target sites. Such abrogation of transcriptome-wide bindings of miR-124-6pi and targets was also demonstrated in the evident miR-124 targets, which were repressed by miR-124 and also contained seed sites (*P*=0.30, KS-test, Fig. 6b) or nucleation bulge sites (*P*=0.11, KS-test, Fig. 6c) in *de novo* Ago–miR-124 clusters.

Most of the miR-124 targets are known to function in neuronal differentiation^29^, explaining the neurite outgrowth phenotype induced by expression of miR-124 (ref. 39) in neuroblast-like cells such as Neuro2a (N2a)^38^,^40^. As disturbances of miR-124 function were expected in miR-124-6pi, this possibility was first confirmed by integrated analysis of *de novo* Ago–miR-124 clusters^26^,^29^ and RNA-Seq data on biological pathways, with focus on regulation of the actin cytoskeleton (Supplementary Fig. 11a). One of the well-validated critical miR-124 targets, integrin β-1 (ITGB1)^29^,^39^, was analysed (Fig. 6d). Consistent with the results (Figs 5e,f), ITGB1 mRNA was derepressed completely by the dSpacer pivot substitution but only marginally by 2′-OMe (Fig. 6d). In addition, RNA-Seq analysis demonstrated that 2′-OMe could not sufficiently block the global repression of off-targets as much as the dSpacer pivot substitution in siRL (Fig. 6e). In N2a cells, miR-124-6pi was found to lose its function of inducing neurite outgrowth (Fig. 6f). In contrast, miR-124 containing 2′-OMe still induced neurosphere-like structures and differentiation (Fig. 6f, Supplementary Fig. 11b and Supplementary Movies 1–3). These data showed that abasic pivot substitution abolishes miRNA-like target interactions and repression, enough to prevent miRNA-induced biological phenotypes.

### *In vivo* application of abasic pivot substitution

**Figure 7:**
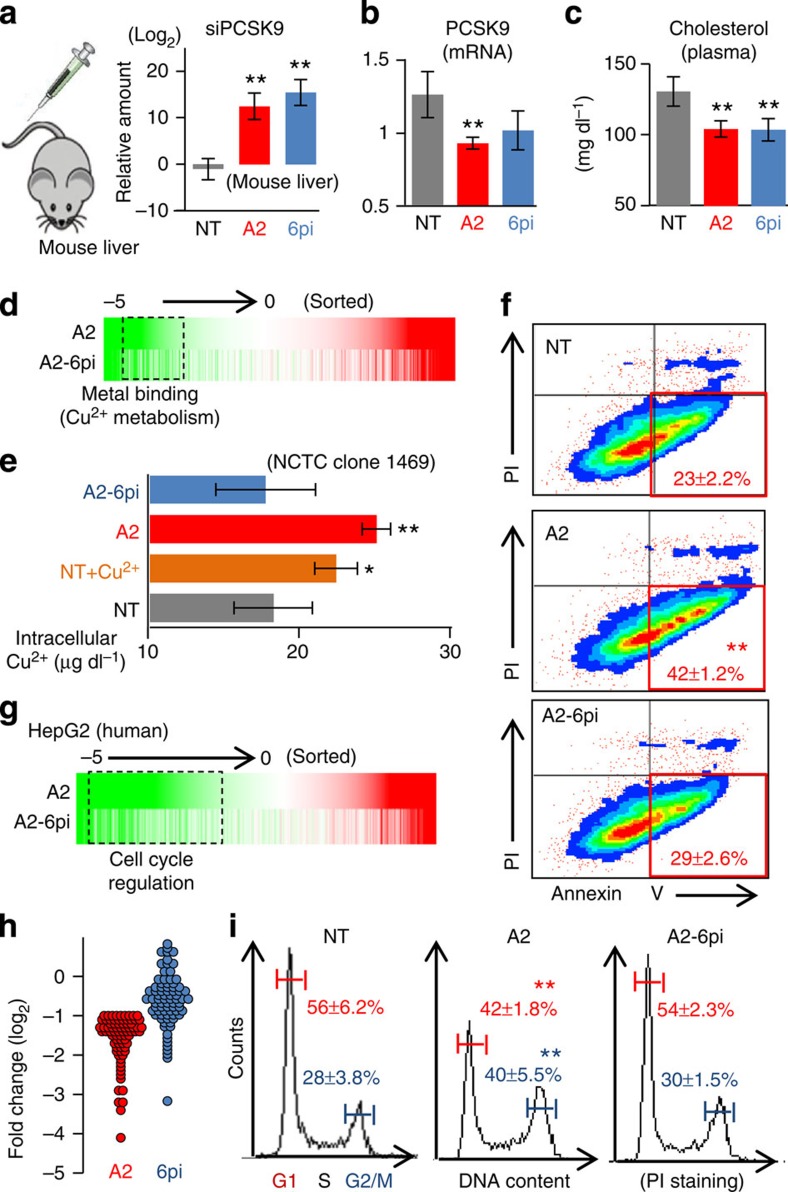
Abasic pivot substitution eliminates miRNA-like off-target effect *in vivo*. (**a**) *In vivo* delivery of siRNAs (5 mg kg^−1^) to the mouse liver was confirmed by measuring the amount of siPCSK9-A2 or siPCSK9-A2-6pi in the liver (qPCR, indicated as log_2_ ratio relative to NT, normalized to *U6*, *n*=5) after tail vein injection; error bars, s.d. The double asterisk denotes *P*<0.01 (*t*-test). (**b**) The same analysis as in **a**, except *PCSK9* mRNA is quantified to estimate on-target activity (relative amount to NT, normalized to GAPDH, *n*=5). (**c**) The concentration of plasma cholesterol, measured by a quantitative colorimetric cholesterol determination assay (*n*=5). (**d**) The same transcriptome-wide analysis of off-target repression comparing siPCSK9-A2 with siPCSK9-A2-6pi in the mouse liver, as in the upper panel of Fig. 6a. GO analysis elucidated the enrichment of metal binding function (including ‘copper metabolism') among the off-targets (Supplementary Table 4). (**e**) The amount of intracellular copper in NCTC clone 1469 cells was significantly increased by siPCSK9-A2 (25±2.2 μg dl^−1^, *P*<0.01, relative to NT, *t*-test, *n*=3), but not by siPCSK9-6pi (16±5.0 μg dl^−1^). ‘Cu^2+^' indicates treatment with 32 μM CuSO_4_. A single asterisk denotes *P*<0.05 (*t*-test). (**f**) Cell death assays of NCTC clone 1469 cells measured by FACS analysis with propidium iodide (PI) and Annexin V staining. The percentage of cells in the phase of early apoptosis (red box, mean±s.d., *n*=3) was significantly increased by siPCSK9-A2 but not by siPCSK9-A2-6pi (details in Supplementary Fig. 12d). (**g**) The same transcriptome-wide analysis of off-targets as in **d** except on HepG2 cells. (**h**) Comparison of fold changes between siPCSK9-A2 and siPCSK9-A2-6pi, in a set of off-target transcripts functioning in cell cycle regulation, identified by GO analysis in 6pi-dependent derepressed transcripts (dotted box in **g**, Supplementary Fig. 13c–f). (**i**) Cell cycle analysis of HepG2 cells, performed by FACS analysis using PI staining. A defect in cell cycle regulation was observed with siPCSK9-A2, but not with siPCSK9-A2-6pi (*n*=3).

Effectiveness of the abasic pivot substitution was then tested *in vivo* on siRNA targeting PCSK9 (siPCSK9-A2)^36^. First, siRNAs were delivered to mouse liver tissues and validated by quantitative PCR (qPCR) analysis of siPCSK9-A2 and siPCSK9-A2-6pi in the liver (*P*=8.9 × 10^−7^, one-way analysis of variance (ANOVA); Fig. 7a). PCSK9 mRNA in the liver was reduced (*P*=0.001, one-way ANOVA; Fig. 7b) by either siPCSK9-A2 or siPCSK9-A2-6pi (*P*<0.06, *t*-test, relative to NT; Fig. 7b), which was sufficient to cause significant reduction of plasma cholesterol (*P*<0.01, *t*-test; *P*=0.006, one-way ANOVA; Fig. 7c). Next, siRNA-dependent global off-target repression was further investigated through RNA-Seq analysis (Supplementary Table 3b). Substantial numbers of siPCSK9-A2 off-targets were identified (Fig. 7d) as significantly derepressed by the dSpacer pivot substitution (*P*=8.0 × 10^−5^, KS-test, Supplementary Fig. 12a).

To explore the expected phenotypic consequences of this off-target repression, gene ontology (GO) analysis was performed and revealed that functional categories related to ‘metal ion binding' were enriched in siPCSK9-A2 off-targets (Fig. 7d and Supplementary Table 4). Some of these target genes are known to regulate copper metabolism (metallothionein 1 and metallothionein 2; Supplementary Table 4). Consistent with these findings, the concentration of intracellular copper (17±4.1 μg dl^−1^) was significantly increased depending on siPCSK9-A2 expression (25±2.2  μg dl^−1^) in the mouse liver cell line (NCTC clone 1469, Fig. 7e). These effects are similar to those caused by exposure to increased plasma copper concentration (∼32 μM) in Wilson disease^41^ (22±3.1 μg dl^−1^, Fig. 7e). siPCSK9-A2 also significantly induced apoptotic cell death (∼19% increase, *P*<0.01, *t*-test, Fig. 7f and Supplementary Fig. 12d) as observed in copper-induced apoptosis (Supplementary Fig. 12c)^41^. The same off-target phenotypes were also observed with siPCSK9-A1 (Supplementary Fig. 12b,c). Moreover, all of these off-target phenotypes disappeared when they contained the dSpacer pivot substitution (Fig. 7e,f and Supplementary Fig. 12b–d).

As miRNA-like off-target effects were reported to be species-specific^42^, the human liver cell line (HepG2) was used to evaluate the potentially deleterious off-target effects and their possible prevention by the abasic pivot substitution in humans, a clinically important application. RNA-Seq analysis for transcript profiles depending on siPCSK-A2 expression (Supplementary Table 3c) showed widespread off-target transcripts in human liver cells (Fig. 7g), whereas the dSpacer pivot substitution significantly derepressed such off-targets (*P*=1.1 × 10^−9^, KS-test, Supplementary Fig. 13a). This included putative direct miRNA-like off-targets that contain seed sites (*P*=0.01, KS-test, Supplementary Fig. 13b).

To determine the off-target phenotypes, the GO functions of the derepressed transcripts were also analysed (Supplementary Fig. 13c,d). Cell cycle regulation was identified as the most likely affected phenotype, based on comparisons of fold changes between siPCSK9-A2 and siPCSK9-A2-6pi (Fig. 7h and Supplementary Fig. 13e,f). This finding was confirmed by functional pathway analysis (Supplementary Fig. 14a). Cell cycle analysis of siPCSK9-A2-expressed HepG2 showed that ∼12% of the cells were significantly decreased in G1/S but increased in G2/M, whereas siPCSK9-A2-6pi did not cause these phenotypes (Fig. 7i). The same results were observed with siPCSK9-A1 (Supplementary Fig. 14b) and such off-target effects were found to be species-specific (Supplementary Fig. 15). Taken together, we conclude that unexpected off-target phenotypes are inevitably caused by siRNAs *in vivo*, but the dSpacer pivot substitution can also be used *in vivo* to eliminate such adverse off-target effects.

## Discussion

RNAi has now become one of the most widely used methods for gene silencing because of specific inhibition of gene expression and ease of use^1^. It has been applied to study various genes for loss of function and develop therapeutic applications to knock down disease-causing genes^20^. Our initial assumption about the specificity of RNAi was found to be relative, only indicating that on-target repression activity is stronger than off-target repression^8^. Chemical modifications such as 2′-OMe^13^ and UNA^21^ have been utilized to reduce the off-target effects, but their inhibitory effect on miRNA-like off-targets is limited, as we found in this study. This may be because such modifications have been applied only to the nucleotide backbone rather than to the bases, which directly participate in target recognition. On the basis of this notion, the effectiveness of abasic substitution in blocking off-target activity and selecting specific alleles^43^ can be explained.

**Figure 8:**
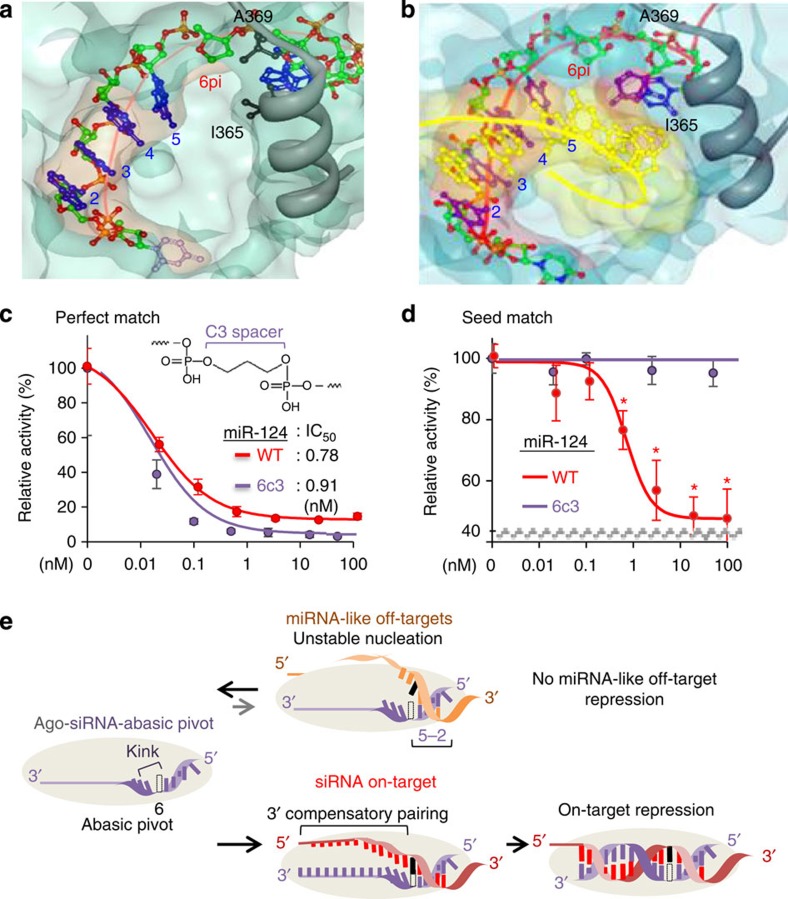
Models of siRNA containing abasic pivot substitution implicated by the usage of c3 spacer. (**a**) A surface model of Ago–miRNA structure with dSpacer pivot substitution (6pi), presented as in Fig. 1a (from 4F3T^24^ in PDB). Of note, 6pi reduced steric hindrance by generating space (4.9 Å from I365, 5.1 Å from A369) in the kink between position 6 and 7 (3.6 Å from I365, 3.9 Å from A369, Fig. 1a). Details are provided in Supplementary Fig. 16. (**b**) A surface model of Ago–miRNA–target structure with 6pi (from 4W5O^25^ in PDB). Target mRNA is yellow. (**c**) The C3 spacer (upper panel) substitution for pivot (6c3) was applied to miR-124 and its effect on repressing perfectly matched sites was analysed by estimating IC_50_ using luciferase reporter assays, as in Fig. 5a. (**d**) Effect of 6c3 on seed-mediated target repression was measured for miR-124 by luciferase reporter assays (IC_50_[6c3]=ND, IC_50_[WT]=0.07 nM) as in Fig. 5e. ND, not determined. Asterisk denotes *P*<0.01 (*t*-test, *n*=6). (**e**) miRNA-like off-target sites mediated by siRNA seed regions may be unable to induce stable transitional nucleation owing to abasic pivot substitution, avoiding miRNA-like off-target recognition and repression (upper panel). In contrast, the on-target site that is perfectly complementary to siRNA except for the abasic pivot overcomes unstable transitional nucleation (only four consecutive base pairs in positions 2–5) and the structural kink by 3′ compensatory pairing with help of the reduced steric hindrance (as modelled in **b**). This event leads to formation of the siRNA–target duplex and on-target repression (lower panel). Shaded ovals represent the Ago protein.

As there is a trade-off between on-target activity and derepression of off-targets, a critical issue regarding any method that blocks off-targets is how effectively on-target activity can be maintained. Structural studies showed that the base and 2′-hydroxyl group (2′-OH) of a pivot nucleotide are located at a close distances from the α-helix of human Ago (base, 3.6 Å from I365; 2′-OH, 3.9 Å from A369), which generates a kink after the pivot position (positions 6–7, Fig. 1a)^23^,^24^. Analysis of models derived from the human Ago–miRNA structure^23^ elucidated that dSpacer, which contains neither a base (4.9 Å from I365) nor 2′-OH (5.1 Å from A369), has less potential to cause steric hindrance (Fig. 8a and Supplementary Fig. 16a–c). In a ternary structure of the human Ago2–RNA–target, the kink in Ago–miRNA structure is absent by moving out the α-helix and a perfectly matched target widens central cleft of Ago to accommodate target pairing beyond position 8 (ref. 25). On the basis of the model derived from the ternary structure^25^, dSpacer pivot substitution seems to provide adequate space to be beneficial for such stepwise conformational transitions: moving out the kink and widening the central cleft (Fig. 8b), requiring to be further stabilized by pairing to position 8 and/or position 13–16^25^. Although 2′-OH of pivot nucleotide does not make a hydrogen bond interaction^23^,^24^,^25^, positions 2 to 7 (including pivot) of the miRNA–target duplex make extensive hydrophobic and van der Waals interactions with aliphatic segments within α-helix and PIWI domain^25^. Therefore, removing 2′-OH of pivot nucleotide may increase Ago target affinity, resulting in conservation of on-target activity. In support, other abasic conformations, which either involve rSpacer (6pi-r, Fig. 3b) or cause an abasic bulge (6pi-b, Fig. 3d; 6pi-rb, Fig. 3f), showed less efficient on-target activity than dSpacer pivot substitution.

This notion was further examined by using C3 spacer (Fig. 8c, upper panel) which consists of three carbons minimally required for the linker function (Fig. 8c), found to have the least bulky structure without 2′-OH in pivot (6.4 Å from I365, 6.2 Å from A369, Supplementary Fig. 16d). As expected, C3 spacer substitution elicited excellent repression activity for a perfectly matched on-target (Fig. 8c and Supplementary Fig. 17a,b) without inducing seed-mediated target repression (Fig. 8d and Supplementary Fig. 17c,d). Therefore, destabilizing nucleation pairings by the abasic pivot substitution may enable siRNA to avoid miRNA-like target recognition (Fig. 8e, upper panel), but facilitate to transit from weak nucleation to functional interaction with the perfectly matched on-target (Fig. 8e, lower panel), especially in the case where the abasic pivot has reduced potential of steric hindrance via abasic and no 2′-OH (for example, dSpacer, C3 spacer).

Although abasic pivot substitution conserves the on-target activity of siRNAs (∼80-100% of maximal silencing activity, relative to siRNAs without a spacer), there is some variation in its efficiency among different siRNAs (≤9-fold decrease in IC_50_, Figs 2 and 5). Such variation of on-target activity possibly depends on the sequence composition and secondary structure of target mRNAs, which is reminiscence of the general properties of RNAi^1^. Most of siRNAs containing abasic pivot substitution showed the same on-target activity (100%) as unmodified versions in the concentration range generally used for siRNA transfection into cell cultures (10–75 nM, Figs 2 and 5; except for siRL of which on-target activity is ∼80%). However, further studies should be performed to determine features of siRNA sequences, which perform the same on-target activity even when they contain abasic pivots (Fig. 5b,d), an important issue for clinical application to avoid dose-limiting toxicity.

By substituting pivot with an abasic spacer, no significant miRNA-like off-target repression was observed in every concentration range or even the maximal concentration of siRNA transfection (100 or 150 nM, Supplementary Fig. 18a). dSpacer pivot substitution did not change the amount of siRNAs loading into Ago (Supplementary Fig. 18b,c), retained the ability to cleave (Fig. 2i and Supplementary Fig. 18f) and degrade on-target mRNAs (Supplementary Fig. 18d,e). Although miRNA-like off-target effects were eliminated by the abasic pivot substitution, there was still remaining off-target effect causing an innate immune response against double-strand RNAs—the dSpacer pivot substitution was observed to have no effect on TLR3-mediated innate immune responses (Supplementary Fig. 18g). Therefore, it is suggested that abasic pivot substitution should be combined with some other modification methods that can prevent such innate immune responses, as we tried and observed in the case of siPCSK9-A2-6pi (Figs 5d,h and 7).

The abasic pivot substitution is broadly applicable to a wide range of RNAi usages. Although all of the siRNA sequences used in this study were designed with consideration of off-targeting^16^,^19^,^44^, they all showed significant off-target repression. With long-term therapeutic application, the off-target effects of siRNA may become serious, as implicated in this study of PCSK9 siRNAs. The abasic pivot substitution is essential to apply RNAi for experimental and clinical purposes, where ensuring the specificity is especially important.

## Methods

### Bioinformatics

In general, for the bioinformatics analysis, we mainly used Python scripts, UCSC genome browser (http://genome.ucsc.edu/), and Galaxy (http://galaxy.psu.edu), as described previously^26^,^29^. For the analysis of miRNA target sites, we used miRTCat (http://ion.skku.edu/mirtcat/)^30^. Hierarchical clustering was performed by using Cluster program and visualized as a heat map by using Treeview (http://rana.lbl.gov/EisenSoftware.htm), as described previously^26^,^29^. GO analysis was performed using DAVID (http://david.abcc.ncifcrf.gov/) with default parameters unless otherwise indicated. Pathway analysis was performed and visualized by KEGG (http://www.genome.jp/kegg/). All the statistical tests were done by using Scipy (http://www.scipy.org/) or Excel (two-sided test). Structural modelling and analysis were performed by using PyMOL (http://www.pymol.org/) and Protein Workshop in Protein Data Bank (PDB, http://www.rcsb.org/) based on human Ago2–miRNA (4F3T^24^, 4OLA^23^) and Ago2–miRNA–target structures (4W5O^25^).

### Prediction of miRNA-like target sites

Basically, all 6mer matches to seed sequences in position 1–8 were searched to identify seed sites (6–8 nucleotides) in 3′-UTR or whole transcripts (defined by RefSeq, downloaded from the UCSC genome browser). To search nucleation bulge sites, 7mer matches were derived from 6mer seed matches (position 2–7) in which the nucleotide of the position 5–6 target mRNA bulge sequence is complementary (Watson–Crick base pairing) to position 6 (pivot) of the corresponding siRNAs or miRNAs, as described previously^26^,^30^. For comparison of the seed and the nucleation bulge sites, we used only 7mer seed matches with lengths that are the same as those of 7mer nucleation bulge patterns.

### Meta-analysis of microarray data

Meta-analysis was performed by obtaining normalized compiled data^35^ from microarray experiments using 35 different siRNA transfections (35 different sequences, targeting nine transcripts; Supplementary Table 1a) or 21 siRNA transfections (eight different sequences, all targeting MAPK14; Supplementary Table 1b)^13^. For meta-analysis regarding 2′-OMe, normalized microarray data from the expression of seven different sequences of siRNAs as pairs (no modification versus 2′-OMe) were downloaded from the Gene Expression Omnibus (GEO) database (http://www.ncbi.nlm.nih.gov/geo/), in entirely or selectively for transcripts with significant differences (P<0.05). To select potent siRNA off-target transcripts, we predicted miRNA-like off-target sites (seed sites or nucleation bulge sites). Cumulative fraction analyses depending on fold change (log_2_ ratio) were performed only for coding transcripts (RefSeq IDs start with NM) and were analysed as described previously^26^,^29^. KS-tests were performed by using Scipy (scipy.stats.ks_2samp()).

### siRNA synthesis and modification

Custom synthesis and modification services of TriLink Biotechnologies (USA), ST Pharm (Korea) and Bioneer (Korea) were used to generate siRNAs with a modification. dSpacer (abasic deoxynucleotide), rSpacer (abasic ribonucleotide) and C3 (propyl) spacer were introduced at the indicated positions. The 2′-OMe^13^ was introduced at position 2 and UNA^21^ was introduced at position 7, as reported previously^13^,^21^. Duplex forms of siRNAs against *Renilla* luciferase (siRL, guide strand: 5′p-GUAGGAGUAGUGAAAGGCCdTdT-3′, passenger strand: 5′p-GGCCUUUCACUACUCCUACdTdT-3′, ‘dT' indicates thymidine deoxynucleotide), siRNAs against *PCSK9* (siPCSK9-A1, guide strand: 5′-UUCCGAAUAAACUCCAGGCdTdT-3′, passenger strand: 5′p-GCCUGGAGUUUAUUCGGAAdTdT-3′; siPCSK9-A2, guide strand: 5′p-UUCCGAAuAAACUCcAGGCdTdT-3′, passenger strand: 5′-GccuGGAGuuuAuucGGAAdTdT-3′, lower case indicates 2′-OMe modification)^36^, and siRNA against *MAPK14* (siMAPK14, guide strand: 5′p-AACCGCAGUUCUCUGUAGGdTdT-3′, passenger strand: 5′p-CCUACAGAGAACUGCGGUUdTdT-3′)^9^ were produced *in vitro* by following the reaction (90 °C for 2 min, 30 °C for 1 h and 4 °C for 5 min). The miRNAs (mmu-miR-124-3p, mmu-miR-708 and cel-miR-67) that we used here were synthesized and duplexed with the same sequences in miRBase (http://www.mirbase.org/). A non-targeting siRNA (NT) that was derived from cel-miR-67 (*C. elegans-*specific miRNA provided as a negative control by Dharmacon) was further modified by introducing 2′-OMe (position 1 and 2) in both guide and passenger strands (guide strand: 5′p-uaCUCUUUCUAGGAGGUUGUGAdTdT-3′, passenger strand: 5′ p-ucACAACCUCCUAGAAAGAGUAdTdT-3′), which blocked both on-target and off-target repression according to previous study^13^.

### Cell culture and transfection

The human cervical adenocarcinoma cell line HeLa (ATCC CCL-2), human hepatocellular carcinoma cell line HepG2 (Korean Cell Line Bank) and mouse neuroblastoma cell line Neuro-2a (N2a, ATCC CCL-131) were maintained in Dulbecco's modified Eagle's medium (DMEM; Gibco) supplemented with 10% fetal bovine serum (FBS, Gibco), 100 U ml^−1^ penicillin, and 100 μg ml^−1^ streptomycin at 37 °C with 5% CO_2_ incubation. The mouse liver cell line NCTC clone 1469 (Korean Cell Line Bank) was maintained either in the same medium or with 10% horse serum (WelGENE) instead of FBS. NCTC clone 1469 cells were transfected by using Lipofectamine 2000 (Invitrogen), whereas HepG2 and N2a cells were transfected by using RNAiMAX (Invitrogen) with 50 nM RNA duplexes according to the general protocol provided by the manufacturer, unless otherwise indicated. Transfection into HeLa cells was performed as described previously^26^. The cells were generally collected 24 h after transfection in all experiments, unless otherwise indicated.

### Luciferase reporter assays

Luciferase reporter assays were performed as described previously^26^. In brief, psiCheck-2 plasmids (Promega) were co-transfected with duplexed siRNAs or miRNAs by using Lipofectamine 2000 (Invitrogen). Twenty-four hours after transfection into HeLa cells, relative activity (*Renilla* luciferase activity normalized to firefly luciferase) was measured by Dual-Luciferase Reporter Assay System (Promega) with the GloMax-Multi Detection System (Promega) with replicates (*n*=6) according to the manufacturer's protocol. In general, IC_50_ was calculated by performing nonlinear least squares fitting for the sigmoid function using Scipy (scipy.optimize.curve_fit()). In cases where least squares failed to fit the function, an approximate IC_50_ was calculated from the regression line.

### Construction of luciferase reporters

To measure the on-target activity of siRL, the psiCheck-2 vector (Promega) was used. In the 3′-UTR of synthetic *Renilla* luciferase, we inserted two seed match sites (position 2–8) for measuring off-target repression to ensure sensitivity of the reporters and a perfect match site for measuring on-target repression. In general, synthetic duplex oligos (Bioneer, Korea) containing various target sites were cloned into the psiCheck-2 plasmid (PCSK9, forward: 5′-TCGAGGCCTGGAGTTTATTCGGAAGC-3′, reverse: 5′-GGCCGCTTCCGAATAAACTCCAGGCC-3′; PCSK9-Seed, forward: 5′-TCGAGATTCGGAAATTCGGAAGC-3′, reverse: 5′-GGCCGCTTCCGAATTTCCGAATC-3′; PCSK9-Nuc, forward: 5′-TCGAGATTTCGGAAATTTCGGAAGC-3′, reverse: 5′-GGCCGCTTCCGAAATTTCCGAAAUC-3′;miR-708-Seed, forward: 5′-TCGAGAGCTCCTTAGCTCCTTGC-3′, reverse: 5′-GGCCGCAAGGAGCTAAGGAGCTC-3′; Cel-miR-67-Seed, forward: 5′-TCGAGGGTTGTGAGGTTGTGAGC-3′, reverse: 5′-GGCCGCTCACAACCTCACAACCC-3′; miR-124, forward: 5′-TCGAGGGCATTCACCGCGTGCCTTAGC-3′, reverse: 5′-GGCCGCTAAGGCACGCGGTGAATGCCC-3′; MAPK14, forward: 5′-TCGAGCCTACAGAGAACTGCGGTTGC-3′, reverse: 5′-GGCCGCAACCGCAGTTCTCTGTAGGC-3′; MAPK14-Seed, forward: 5′-TCGAGCTGCGGTTCTGCGGTTGC-3′, reverse: 5′-GGCCGCAACCGCAGAACCGCAGC-3′) through XhoI and NotI sites. To test seed and nucleation bulge sites of miR-124, we used previously constructed luciferase reporters derived from a G-bulge site in *Mink1* (ref. 26). To evaluate the off-target effects of siRL, synthetic *Renilla* luciferase gene in psiCheck-2 was switched with different sequences of this gene in the pRL-TK plasmid (Promega), of which fragment was amplified (forward: 5′-TATAGGCTAGCCACCATGAC-3′) to contain downstream seed matches (reverse: 5′-ATTACTCGAGTAGGAGTGTAGGAGTCGAAGCGGCCGCTCTAG-3′) or nucleation bulges (reverse: 5′-ATTACTCGAGTAGGAAGTGTAGGAAGTCGAAGCGGCCGCTCTAG-3′) with NheI and XhoI sites or seed matches of the passenger strand (reverse: 5′-CGAAGCGGCCGCGGCCTTTCGGCCTTTCCTCGAGGCCGCTCTAGAATTATTGTTC-3′) with NheI and NotI sites.

### *In vitro* Ago2 cleavage assay

Slicing activity of the siRNA-loaded RISC was assessed by performing *in vitro* Ago2 cleavage assay as described^45^, with some modifications. Briefly, guide strands of let-7 (hsa-let-7a), let-7-6pi, siPCSK9-A1 and siPCSK9-A1-6pi were synthesized (Bioneer, TriLink Biotechnologies) and loaded onto the recombinant human Ago2 protein (Sino Biological Inc.) *in vitro* by incubating them at 37 °C for 1 h (20 mM Tris-HCl, 150 mM NaCl, and 1 mM MgCl_2_). Their cognate substrates (let-7, 5′-ACUAUACAACCUACUACCUCGUUUUUUUUUUUUUUUU-3′; siPCSK9-A1, 5′-GCCUGGAGUUUAUUCGGAAUUUUUUUUUUUUUUUU-3′) were also synthesized, further 5′-end labelled with γ-P^32^ ATP by T4 PNK (NEB) and purified by G25 column (GE Healthcare). Subsequently, the labelled substrate was added to the Ago2-guide strand complex and incubated at 37 °C for 1 h. The reaction was stopped by boiling with denaturation buffer (NEB) for 5 min, and resolved on a 15% denaturing polyacrylamide gel.

### Immunoblotting

Cell lysates were prepared using 1 × RIPA lysis buffer (Biosaesang) supplemented with a protease inhibitor (Roche), cleared by centrifugation at 12,000*g*, separated by SDS–PAGE, and transferred onto PVDF membranes (Millipore). Primary antibodies were incubated as indicated; overnight at 4 °C in 1 × TBST with 5% skim milk or 5% BSA; PTBP1 (1:1,000, Invitrogen), MAPK14 (1:1,000, Cell Signaling Technology), PCSK9 (1:1,000, Cayman Chemical Company), γ-tubulin (1:1,000, Cell Signaling Technology) and α-tubulin (1:1,000, Cell Signaling Technology).

### Northern blot for small RNAs

Total RNA was extracted using TRIzol (Invitrogen) and digested with DNase 1 (Qiagen). 10 μg RNA was incubated with formamide (Sigma-Aldrich) at 65 °C for 10 min and separated in 12% acrylamide denaturing (urea) gels. The gels were stained with SYBR-Safe (Invitrogen) to visualize the RNA samples. RNA was transferred to Zeta-Probe GT membranes (Bio-Rad) and cross-linked by ultraviolet irradiation (Stratagene). The membranes were blocked in hybridization buffer (0.5 M Na_2_PO_4_ pH7.2, 15% formamide, 1% BSA and 7% SDS) for 30 min at 37 °C in a rotating hybridization oven. The miR-124 probes (5′-GGCATTCACCGCGTGCCTTA-3′) were labelled with γ-^32^P ATP using PNK (NEB) and cleaned with a G25 column (GE Healthcare). Probes were heated at 65 °C before being added into the hybridization bottle, and membranes were hybridized at 37 °C overnight. The membranes were washed three times with 1 × SSC containing 0.1% SDS, and then was exposed to X-ray film for 1 day at −80 °C.

### RNA-Seq

RNA-Seq libraries were generated from total RNA samples either by the not-so-random priming (NSR) method^46^ or the poly-adenylation cDNA (polyA) method (Supplementary Table 3). For the NSR method, 1 μg of total RNA extracted by RNeasy Mini Kit (Qiagen) was used to construct libraries by following the protocol^46^ up to the second strand synthesis step. A modified protocol involving a series of PCR amplifications was then used to introduce Truseq indexed adaptor sequence (Illumina). In brief, the first PCR was set up as follows: 28 μl of purified DNA, 10 μl of 5 × buffer 2, 5 μl of 25 mM MgCl_2_, 2.5 μl of 10 mM dNTPs, 2 μl of 25 μM NSR-PCR-F-N (5′-AATGATACGGCGACCACCGACACTCTTTCCCTACACGACGCTCTTCCGATCTCT-3′), 2 μl of 25 μM NSR-R-N (5′-CGTGTGCTCTTCCGATCTGA-3′), and 0.5 μl of Expand High-Fidelity Plus Polymerase (Roche); 2 min at 94 °C; 2 cycles of 10 s at 94 °C, 2 min at 40 °C and 1 min at 72 °C; eight cycles of 10 s at 94 °C, 30 s at 60 °C, 1 min at 72 °C; and 5 min at 72 °C. The DNA was purified using Agencourt AMPureXP (Beckman), eluted in 30 μl of water, and subjected to the second PCR reaction as follows: 28 μl of purified DNA, 10 μl of 5 × buffer 2, 5 μl of 25 mM MgCl_2_, 2.5 μl of 10 mM dNTPs, 2 μl of 25 μM NSR-PCR-F-N, 2 μl of 25 μM NSR-R-bar (5′-GTGACTGGAGTTCAGACGTGTGCTCTTCCGATCTGA-3′), and 0.5 μl of Expand High-Fidelity Plus Polymerase (Roche); 2 min at 94 °C; 15 cycles of 10 s at 94 °C, 2 min at 40 °C and 1 min at 72 °C; 15 cycles of 10 s at 94 °C, 30 s at 60 °C, 1 min with an additional 10 s added at each cycle at 72 °C; and 5 min at 72 °C. The DNA was purified with Agencourt AMPureXP beads (Agencourt), eluted in 30 μl of water and subjected to the third PCR reaction as follows: 15 μl of purified DNA, 2.5 μl of 10 × AccuPrime Pfx Reaction mix, 0.4 μl of 25 μM NSR-PCR-F-N, 0.4 μl of 25 μM R-Tag (5′-CAAGCAGAAGACGGCATACGAGAT-6mer barcode-GTGACTGGAGTTCAGACG-3′), 6.5 μl of water and 0.25 μl of AccuPrime Pfx DNA Polymerase (Invitrogen); 2 min at 95 °C; two cycles of 20 s at 95 °C, 30 s at 58 °C and 40 s at 68 °C; and 5 min at 68 °C. The PCR products were run on a 2% agarose gel, excised in the 300- to 500-bp range and gel-purified. NSR RNA-Seq libraries were then sequenced by HiSeq 2,000 system (Illumina) as 100 single-end reads and demultiplexed by CASAVA (Illumina). For the polyA method, RNA-Seq libraries were constructed, sequenced and de-multiplexed by Otogenetics (Norcross, GA, USA) using a HiSeq 2000 platform for 100 nt pair-end reads.

### Analysis of RNA-Seq and Ago HITS-CLIP data

The demultiplexed sequencing reads were aligned to the human genome (hg19) or mouse genome (mm9) using TopHat2 (ref. 47; pair-end reads, tophat2 -a 4 -g 1 --b2-sensitive -r 100 --mate-std-dev=50 --no-discordant; single-end reads, tophat2 -a 4 -g 1 --b2-sensitive -r 100 --mate-std-dev=50 --library-type fr-unstranded) under a supply of RefSeq gene annotations. Transcript levels were quantified using Cufflinks (cufflinks -N -b) and differential transcript profiles were analysed by Cuffdiff (Cuffdiff --FDR=0.1 -b -N --min-alignment-count=10 --library-norm-method=geometric). Only those values with a valid status were selected. Treeview was used to visualize the data as a heat map. The cumulative fraction depending on fold change (log_2_ ratio) was analysed as described previously^26^,^29^. Depending on the analysis, a ‘significant' call from Cuffdiff was used to select transcripts with significant changes. Ago HITS-CLIP^29^ data were downloaded from the following link (http://ago.rockefeller.edu/ or http://ago.korea.ac.kr/Ago_Clip_data/ or http://ago.korea.ac.kr/pivot/). *De novo* Ago miR-124 clusters were analysed as described previously^26^,^29^. To map miR-124 seed sites in *de novo* Ago miR-124 clusters, 6mer matches to the miR-124 seed (position 1–8) were used.

### miR-124 induced differentiation of N2a cells

NT, miR-124, miR-124-6pi or miR-124-2me (50 nM) was transfected into N2a cells by RNAiMax (Invitrogen) according to the manufacturer's protocol. To acquire the still images, an inverted microscope (Zeiss Axiovert 200) was used. To generate time-lapse images, Lumascope 400 (Etaluma, with × 10 lens) was used with 20-min intervals between 26 and 86 h after the transfection of miR-124 (Supplementary Movie 1), miR-124-6pi (Supplementary Movie 2), or miR-124-2me (Supplementary Movie 3).

### Quantitative RT–PCR analysis

Total RNA was isolated by using RNeasy Mini Kit (Qiagen) through on-column DNA digestion with the RNase-Free DNase Set (Qiagen). Reverse transcription was performed with SuperScript III Reverse Transcriptase (Invitrogen) and oligo(dT) primer. qPCR analysis was performed with the SYBR Green PCR Master Mix (Applied Biosystems) and custom primers (mouse ITGB1, forward: 5′-TTCAGTGAATGGCAACAATG-3′, reverse: 5′-AGCAACCACGCCTGCTAC-3′; human PCSK9, forward: 5′-AGGGGAGGACATCATTGGTG-3′, reverse: 5′-CAGGTTGGGGGTCAGTACC-3′; mouse PCSK9, forward: 5′-TGCAAAATCAAGGAGCATGGG-3′, reverse: 5′-CAGGGAGCACATTGCATCC-3′), using the AB 7300 real-time PCR instrument (Applied Biosystems). All the reactions were run in triplicate with the standard two-step cycling protocol. Relative quantification was calculated by the ΔCT method, using GAPDH (forward: 5′-TGCACCACCAACTGCTTAGC-3′, reverse: 5′-GGCATGGACTGTGGTCATGAG-3′) as a control.

To quantify siRNA, we followed the miRNA qPCR method^48^. Briefly, 1 μg of small RNAs was purified with the miRNeasy Mini Kit (Qiagen), and polyadenylated by using Poly(A) Polymerase (Ambion) in a total of 10 μl volume. Polyadenylated RNAs were then reverse-transcribed by using SuperScript III Reverse Transcriptase (Invitrogen) with 2 μM of oligo(dT) adaptor primer (5′-GCGAGCACAGAATTAATACGACTCACTATAGGTTTTTTTTTTTTVN-3′). qPCR analysis was performed as described above, except with different primers (siPCSK9-A1 or -A2, forward: 5′-TTCCGAATAAACTCCAGGC-3′, reverse: 5′-GCGAGCACAGAATTAATACGACTCAC-3′), cycling conditions (5 min at 95 °C; 45 cycles of 15 s at 95 °C, 15 s at 55 °C and 20 s at 72 °C; and 5 min at 72 °C) and reference control (U6, forward: 5′-CGCTTCGGCAGCACATATAC-3′, reverse: 5′-TTCACGAATTTGCGTGTCAT-3′).

### Cell cycle and cell death analyses

For cell cycle analysis, HepG2 or NCTC clone 1469 were harvested at 48 h after transfection, resuspended (1 × 10^5^ cells) in 200 μl of PBS containing 20 mM EDTA (Sigma-Aldrich), and then fixed with 700 μl of ethanol (Biosesang) for an hour at 4 °C. Then, 10 μl of 1 μg μl^−1^ propidium iodide (PI; Sigma-Aldrich) was added after treatment with 5 μl RNase A (0.2 μg μl^−1^, Sigma-Aldrich) for 30 min at 37 °C. The cells were analysed by BD FACSCalibur (BD Biosciences). Of note, all the cells were synchronized by 24 h serum starvation after the transfection.

For cell death analysis, FITC Annexin V Apoptosis Detection Kit I (BD Pharmingen) was used. In brief, ∼1 × 10^5^ cells in 100 μl of 1 × binding buffer were stained by adding 5 μl of 50 μg ml^−1^ PI and 5 μl of FITC Annexin V for 15 min. After adding 400 μl of 1 × binding buffer, the samples were transferred to 5 ml tubes with cell strainer caps (BD Falcon) and analysed by BD Aria I (BD Biosciences). In this assay, the cells were collected 72 h after the transfection.

### Experiments with mice

siRNAs (5 mg kg^−1^) were administered to 6-week-old male C57BL/6 mice (Orient, a branch of Charles River Laboratories) via intravenous injections using *in vivo*-jetPEI (Polyplus) according to the manufacturer's protocol. The mice were divided into three groups depending on the injected siRNAs (*n*=5 each group, sample size was chosen on the basis of the minimum number used in previous study^36^): NT, A2 and A2-6pi. Briefly, siRNA and *in vivo*-jetPEI complexes (N/P ratio=6) were generated by following the manufacturer's protocol and injected into the tail vein with a sterile syringe (1.0 ml) and a 30-gauge needle. Two days after the injections, liver tissues and blood from the abdominal aorta were collected. Plasma was separated from the blood by centrifugation (2,000*g* for 20 min at 4 °C). All the samples were stored at −80 °C until analysed. Total cholesterol in murine plasma was measured by the Cholesterol E Enzymatic Colorimetric Method (Wako Chemicals) according to the manufacturer's protocol. The excised liver tissues were used for RNA extraction, followed by RNA-Seq and qPCR analysis. This study was reviewed and approved by the Institutional Animal Care and Use Committee (IACUC) of Samsung Biomedical Research Institute (SBRI). SBRI is an Association for Assessment and Accreditation of Laboratory Animal Care International (AAALAC International) accredited facility and abides by the Institute of Laboratory Animal Resources (ILAR) guide.

### Quantification of intracellular copper

NCTC clone 1469 cells were washed once with D-PBS (PBS without Ca^2+^ and Mg^2+^; WelGENE), detached by scraper and resuspended in D-PBS. The same number of cells (∼4 × 10^4^) was prepared by ADAM Automated Cell Counter (NanoEnTek) following the manufacturer's protocol. The cells were lysed by sonication with Bioruptor (Diagenode): 30 s on/off interval for five treatments (5 min total) at high-power settings. The cell lysates were cleared by centrifugation (12,000*g* for 5 min at 4 °C), lysed in 0.5 N NaOH (incubation for 30 min with shaking at 1,000 r.p.m.), and boiled at 100 °C for 30 min. The copper concentration of prepared samples was measured by using QuantiChrom Copper Assay Kits (BioAssay Systems) following the manufacturer's instruction. The cells were collected at 48 h after transfection. As a positive control for this assay, the cells were treated with 32 μM CuSO_4_ and collected 4 h after transfection.

### 5′-end RACE-PCR

Total RNA was extracted from HepG2 cells (24 or 48 h after transfection of 50 nM siPCSK9-A1 or -A2) using RNeasy Mini Kit (Qiagen) through on-column DNA digestion with the RNase-Free DNase Set (Qiagen). The FirstChoice RLM-RACE Kit (Life Technologies) was used according to the manufacturer's manual. Briefly, 1–10 μg of RNA samples was directly ligated with an RNA oligo-nucleotide adaptor (Adaptor 1: 5′-GCUGAUGGCGAUGAAUGAACACUGCGUUUGCUGGCUUUGAUGAAA-3′, Adaptor 2: 5′-AGGGAGGACGAUGCGG-3′) using T4 RNA ligase at 16 °C overnight. In the reverse transcriptase reaction, we used either an oligo(dT) adaptor primer (indicated as ‘RT2', the same primer used in qPCR) or a *PCSK9* specific primer (RT1: 5′-ATTGATGACATCTTTGGCAGAGAAGTGGATCAGTCTCTGC-3′, RT4: 5′-CAGGTTGGGGGTCAGTACC-3′). cDNA was amplified by PCR with various primer sets (primer 1: 5′-GCTGATGGCGATGAATGAACACTG-3′, primer 2: 5′-GCTGAGGCTGGGGAGTAGAGGCAGGCATCGTCCCG-3′, primer 3: 5′-AGGGGAGGACGATGCGG-3′, primer 6: 5′-AGGGAGGACATCATTGGTG-3′, primer 7: 5′-CAGGTTGGGGGTCAGTACC-3′). As a positive control, 5′-end RACE-PCR of CXCR4 mRNA was conducted according to the manufacturer's protocol. Amplified products of the expected size were gel-purified and then verified by sequencing after TA cloning (Takara).

### Quantification of Ago-associated small RNAs

A mixture of 50 nM NT with 50 nM siPCSK-A1 or of 50 nM NT with 50 nM siPCSK9-A1-6pi was transfected into HeLa cells (*n*=3) using Lipofectamine 2000 (Invitrogen). After 24 h incubation, the transfected cells were collected to immunoprecipitate the Ago complex. Briefly, the cells were lysed in lysis buffer (1 × PBS, 0.1% SDS, 0.5% deoxycholate, 0.5% NP-40, protease inhibitor cocktail (Roche), RNAsin (Promega)) and by Bioruptor (Diagenode). The DNA in the lysates was degraded by RQ1 DNase at 37 °C for 5 min. Immunoprecipitation of Ago complex was performed by 2E12 (Abnova) and Dynabead Protein A (Invitrogen) for 4 h at 4 °C. Small RNAs in the complex were then tailed with adenosines using the Poly(A) tailing kit (Ambion) and used to generate cDNA with Superscript III (Invitrogen) and oligo(dT) adaptor primers. The resulting cDNAs were amplified as described in the small RNA qPCR method for quantification of siPCSK9-A1 or siPCS9-A1-6pi associated with Ago (normalized to Ago-associated control NT).

### IFN-β ELISA assay

The siRNA-induced innate immune response was examined by measuring production of IFN-β. HeLa cells in 12-well plates were co-transfected with 200 ng of a plasmid expressing human TLR3 (pcDNA3-TLR3-CFP, a gift from Doug Golenblock, Addgene plasmid # 13641) and siRLs with pi in various positions (75 nM, position 1–6) with Lipofectamine 2000 (Invitrogen), in triplicate. After 24-h incubation, 250 μl of the medium (from 1 ml total) was collected. IFN-β was quantified with the VeriKine Human IFN-β ELISA Kit (PBL Assay Science). A synthetic dsRNA analogs polyinosinic acid-polycytidylic acid (Poly I:C, Sigma) was used as a positive control (1 μg ml^−1^, 24 h incubation).

## Additional information

**Accession codes**: The RNA-Seq data (fastq files) can be accessed through the Sequence Read Archive (SRP047107, SRP047205 and SRP047269) or our project website (http://ago.korea.ac.kr/6pi/).

**How to cite this article:** Lee, H.-S. *et al.* Abasic pivot substitution harnesses target specificity of RNA interference. *Nat. Commun.* 6:10154 doi: 10.1038/ncomms10154 (2015).

## Supplementary Material

Supplementary Information Supplementary Figures 1-18, Supplementary Tables 1-4 and Supplementary References

Supplementary Movie 1 Time-lapse movie of miR-124 transfected N2a cells. The movie begins at 26 hours and ends at 86 hours after the transfection.

Supplementary Movie 2 Time-lapse movie of miR-124-6pi transfected N2a cells. The movie begins at 26 hours and ends at 86 hours after the transfection

Supplementary Movie 3 Time-lapse movie of miR-124-2me transfected N2a cells. The movie begins at 26 hours and ends at 86 hours after the transfection.
