# Supplementary material for: Abasic pivot substitution harnesses target specificity of RNA interference
Source: Nat Commun. 2015 Dec 18;6:10154. doi: 10.1038/ncomms10154 (PMC4703836; doi:10.1038/ncomms10154)
Supplement: Supplementary Information — Supplementary Figures 1-18, Supplementary Tables 1-4 and Supplementary References [file ncomms10154-s1.pdf]

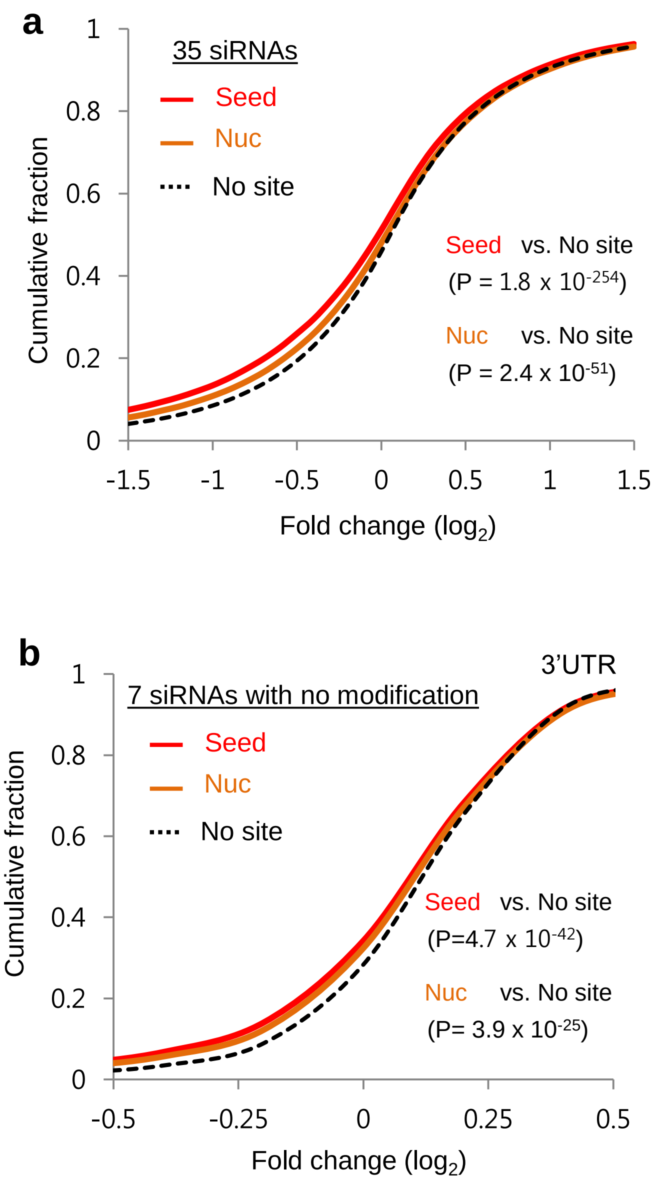

Supplementary Figure 1. Cumulative fraction analyses for miRNA-like off-target effects of siRNAs mediated by seed or nucleation bulge sites. (a) Cumulative fraction analyses for the transcripts containing seed (Seed, red line) or nucleation bulge sites (Nuc, orange line), together with transcripts without any site of these (No site, dotted line) in compiled microarray data from 35 different siRNAs (Supplementary Table 1). P-values are from KS-test. (b-c). The same analysis performed in (a), except only considering the sites in 3'UTR for 7 different siRNAs in Supplementary Table 2A, comparing no modification (b) with 2me (2'-Ome in position 2) (c). Of note, the transcripts containing nucleation bulge sites as well as seed sites showed significant tendency to be downregulated by siRNAs in all analyses regardless of containing 2'-OMe modification.

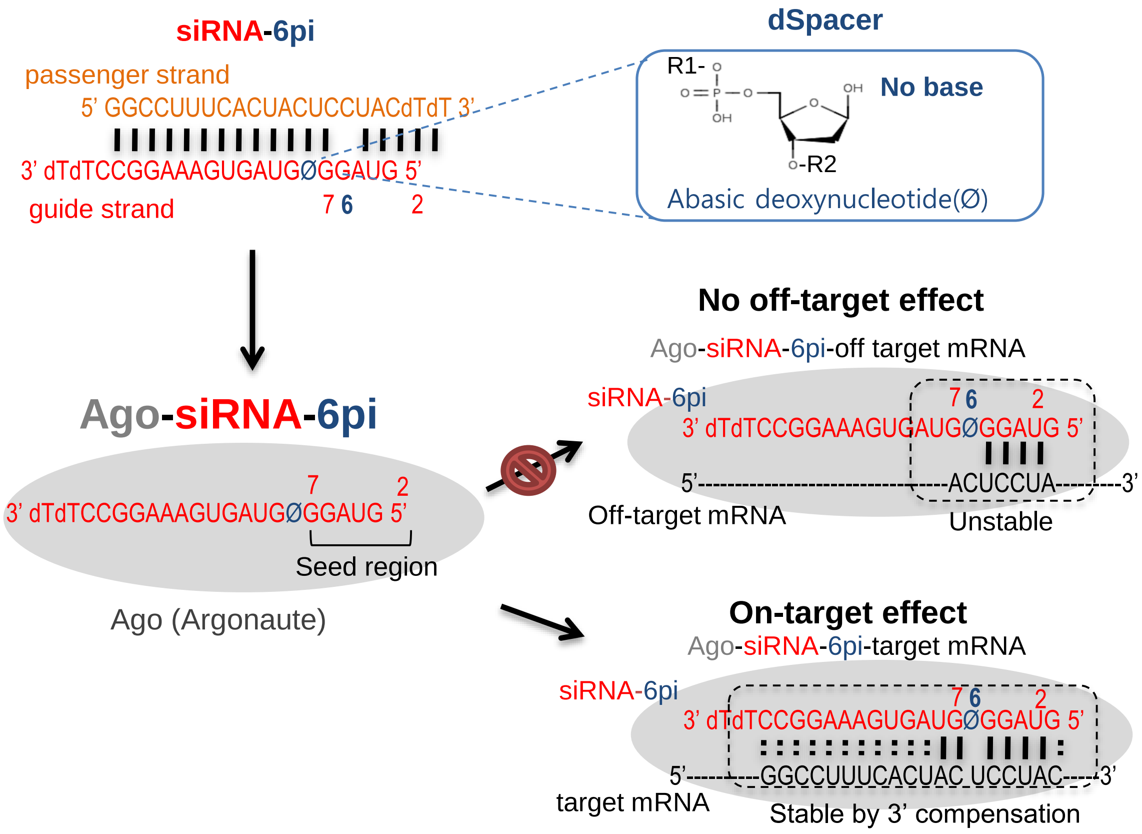

Supplementary Figure 2. A schematic model of siRNA-6pi preventing off-target repression and preserving on-target activity. A schematic model is represented for *Renilla* luciferase siRNA (siRL) containing 6pi (dSpacer pivot substitution) in guide strand, based on transitional nucleation model (Fig. 1b)[9]. The model shows that 6pi could abrogate the miRNA-like off-target repression through destabilizing transitional nucleation and also preserve on-target activity through compensatory near-perfect matches, which stabilize the siRNA-target interaction in the presence of Ago. Of note, 6pi is suspected to be limited to only derepress seed-mediated off-target effects, unable to derepress other off-targets generated by miRNA-like target interactions mediated by regions other than the seed. However, such the non-seed mediated interactions have been reported as minor in miRNA-target interactions[9,10] and even much rare in off-targets of siRNA[4].

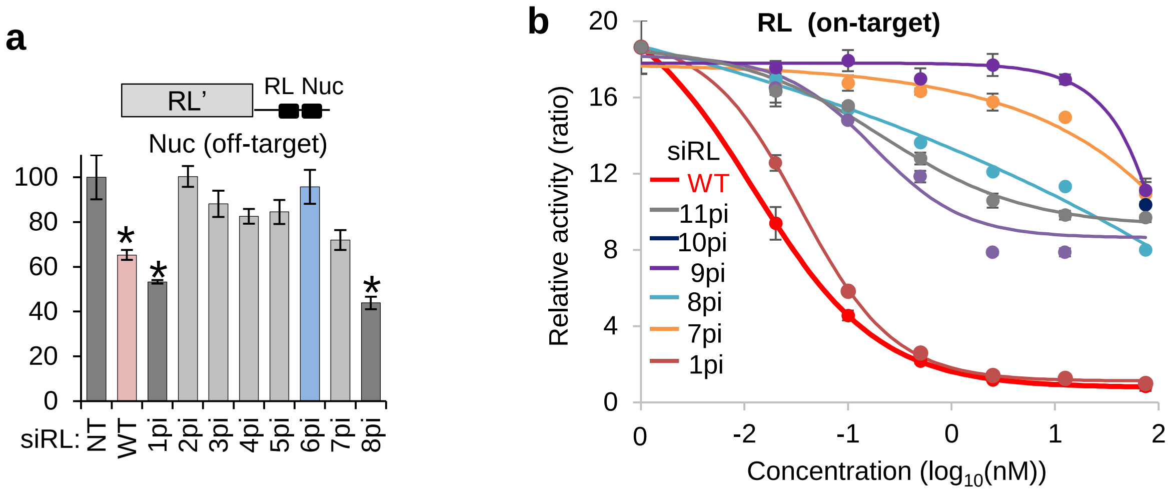

c

siRL	IC_{50} (nM)	I_{max} (%)
WT	0.04	100
1pi	0.05	95
2pi	0.22	77
3pi	0.41	43
4pi	1.12	68
5pi	0.38	75
6pi	0.36	80
7pi	11.3*	42
8pi	6.31*	55
9pi	0.18	42
10pi	62.21*	37
11pi	0.21	45

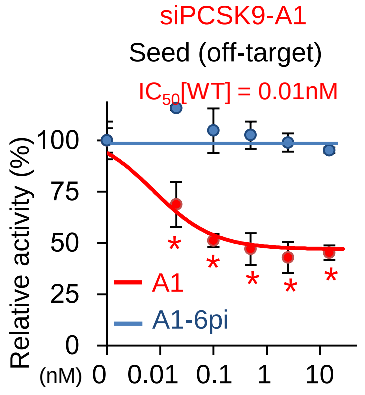

e

A1	IC_{50} (nM)	I_{max} (%)
WT	1.8×10^{-3}	100
2pi	0.04*	93
3pi	0.04*	92
4pi	0.01	93
5pi	0.01	97
6pi	3.4×10^{-3}	100

Supplementary Figure 3. Efficiency of siRNA on-target activity depending on the position of dSpacer substitution (pi). (a) Luciferase reporter assays for miRNA-like off-target repression, mediated by nucleation bulge sites were performed for siRL (75nM) containing dSpacer substitution (pi) as performed in Fig. 2c. (b) Efficiency of on-target repression was measured at different concentrations of siRL with dSpacer substitution (pi) in different position (1-11; 2-6 in Fig. 2d, others in (b)). Relative activity indicates *Renilla* luciferase (RL) normalized to firefly luciferase as ratio (n=6); error bars, s.d.. Concentration of siRNA used in the assay is indicated as log_{10} value. ©. Half maximal inhibitory concentration (IC_{50}) and maximal inhibition rate (I_{max}) of siRL containing pi in different position (1-11), estimated by luciferase reporter assays in Fig. 2c and (b), is represented. In the case where IC_{50} was unable to be calculated by the least squares fitting to sigmoid function, IC_{50} was approximately estimated from the regression line (denoted by an asterisk in the table). (d) miRNA-like off-target activity of siPCSK9-A1, mediated by seed sites, was examined through luciferase reporter assays in the presence and absence (WT) of 6pi, as in Fig. 2h. (e) IC_{50} and I_{max} for siPCSK9-A1 were estimated as in (c). Unmodified siRNA is highlighted with red and 6pi with blue. Of note, 6pi showed the best performance in maintaining on-target activity based on IC_{50} and I_{max} without miRNA-like off-target repression.

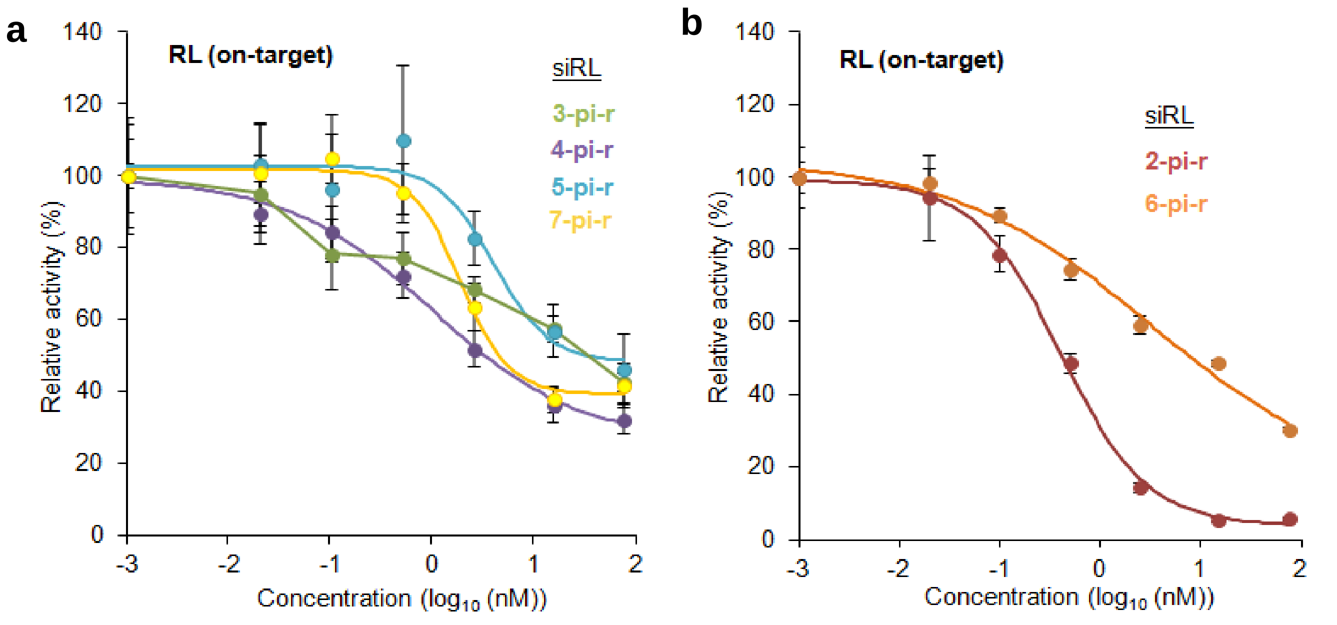

c

siRL	IC_{50} (nM)	I_{max} (%)
2-pi-r	0.41	94
3-pi-r	4.21*	57
4-pi-r	0.99	68
5-pi-r	4.01	54
6-pi-r	3.17	70
7-pi-r	1.91	58

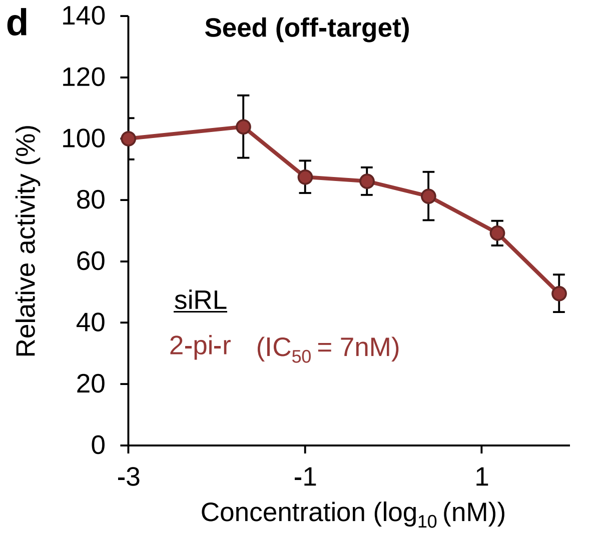

Supplementary Figure 4. On-target and off-target effect of siRNAs containing rSpacer (abasic ribonucleotide) substitution (pi-r) in the seed region. (a-c) Effect of pi-r in siRL (position 2-7) on the on-target activity was examined (a-b) by measuring IC_{50} and I_{max} (c) using luciferase reporter assays as performed in Supplementary Figure 3. (c) The pi-r modification in positions 3-6, that was shown to abolish the off-target repression in Fig. 3a, was highlighted with grey shade. In the case where IC_{50} was unable to be calculated by the least squares fitting to sigmoid function, IC_{50} was approximately estimated from the regression line (denoted by an asterisk in the table). Of note, the pi-r in position 6 is unable to conserve the on-target activity as much as 6pi. (d) Effect of 2pi-r on the seed-mediated off-target repression was measured by estimating IC_{50} due to its superior on-target activity observed in (b). However, siRL-2pi-r has the remaining off-target activity as shown by IC_{50} (7nM). Relative activity indicates *Renilla* luciferase (RL) normalized to firefly luciferase (n=6); error bars, s.d.

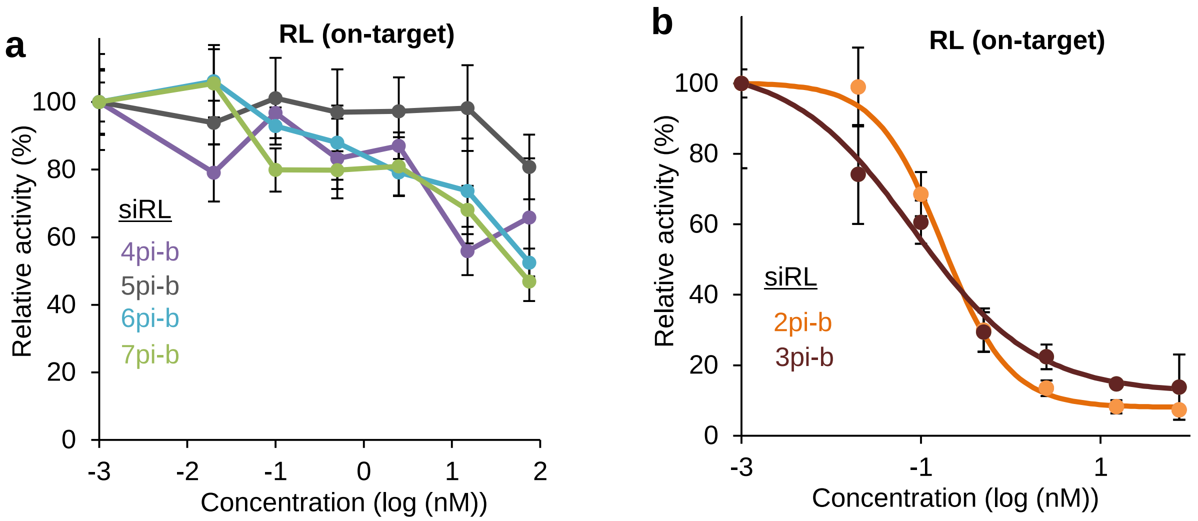

c

siRL	IC_{50} (nM)	I_{max} (%)
2pi-b	0.18	93
3pi-b	0.08	86
**4pi-b**	**3.06**	**34**
**5pi-b**	**n.d.**	**19**
**6pi-b**	**8.03***	**48**
7pi-b	4.71*	53

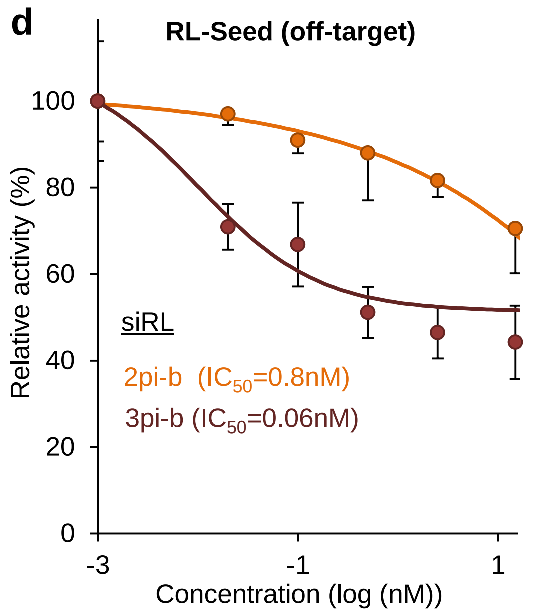

Supplementary Figure 5. Effect of inserting an dSpacer (pi-b) in the seed region (position 2-7) on the on-target activity and the off-target repression. (a-b) pi-b, which generates bulge in abasic position when the given siRNA hybridizes the target site, was introduced into the seed region of siRL and its effect on on-target repression was measured. (c) Calcualtion of IC_{50} and I_{max} using luciferase reporter assays performed in (a) and (b). In the case where IC_{50} was unable to be calculated by the least squares fitting to sigmoid function, IC_{50} was approximately estimated from the regression line (denoted by an asterisk in the table). However, 5pi-b showed no significant repression in all range of siRNA concentration for the on-target activity. Therefore, we indicated it as n.d (not determined). Of note, pi-b modification in position 6 is unable to maintain on-target activity as much as 6pi. (d) Effect of 2pi-b and 3pi-b on the seed-mediated off-target repression was measured by calculating IC_{50} due to its superior on-target activity as shown in (b). However, siRL-2pi-b and siRL-3pi-b has the remaining off-target activity, as shown by IC_{50} (IC_{50}[2pi-b]=0.8 nM, IC_{50}[3pi-b]=0.05 nM). Relative activity indicates *Renilla* luciferase (RL) normalized to firefly luciferase (n=6); error bars, s.d.

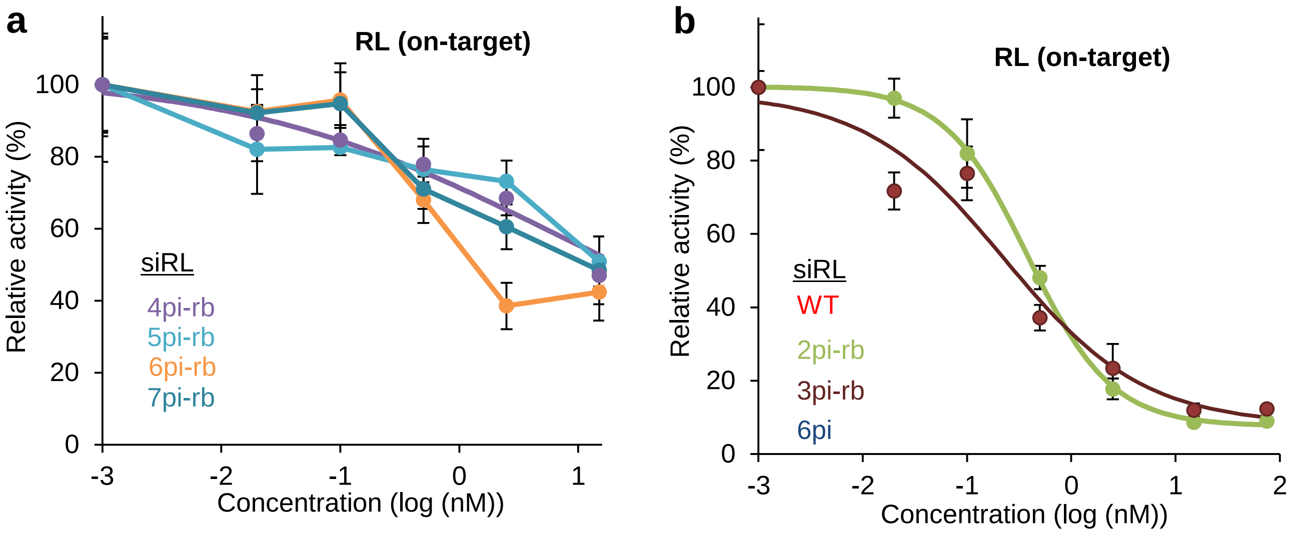

c

siRL	IC_{50} (nM)	I_{max} (%)
2pi-rb	0.38	91
3pi-rb	0.23	88
**4pi-rb**	**5.19**	**49**
**5pi-rb**	**5.22***	**36**
**6pi-rb**	**0.81**	**55**
7pi-rb	0.35	26

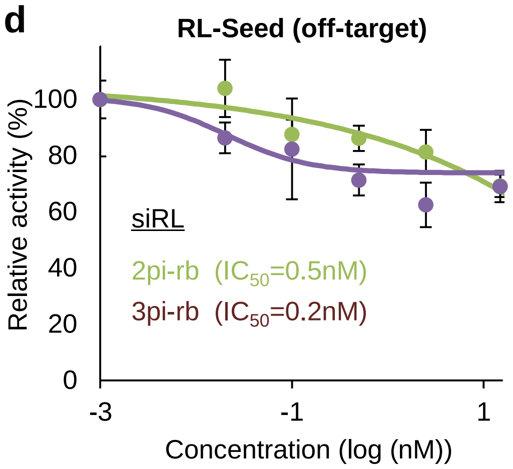

Supplementary Figure 6. Effect of inserting rSpacer (pi-rb) in the seed region on on-target activity and off-target repression. (a-d) The same analysis as performed in Supplementary Figure 5 except using rSpacer (abasic ribonucleotide) instead of dSpacer (abasic deoxynucleotide), which generates bulge in abasic position when the given siRNA hybridizes the target site. Of note, among all the abasic conformations including dSpacer substitution (pi) and rSpacer substitution (pi-r) in the seed region, 6pi performed the best in specificity (Fig 2-3 and Supplementary Fig. 3-6).

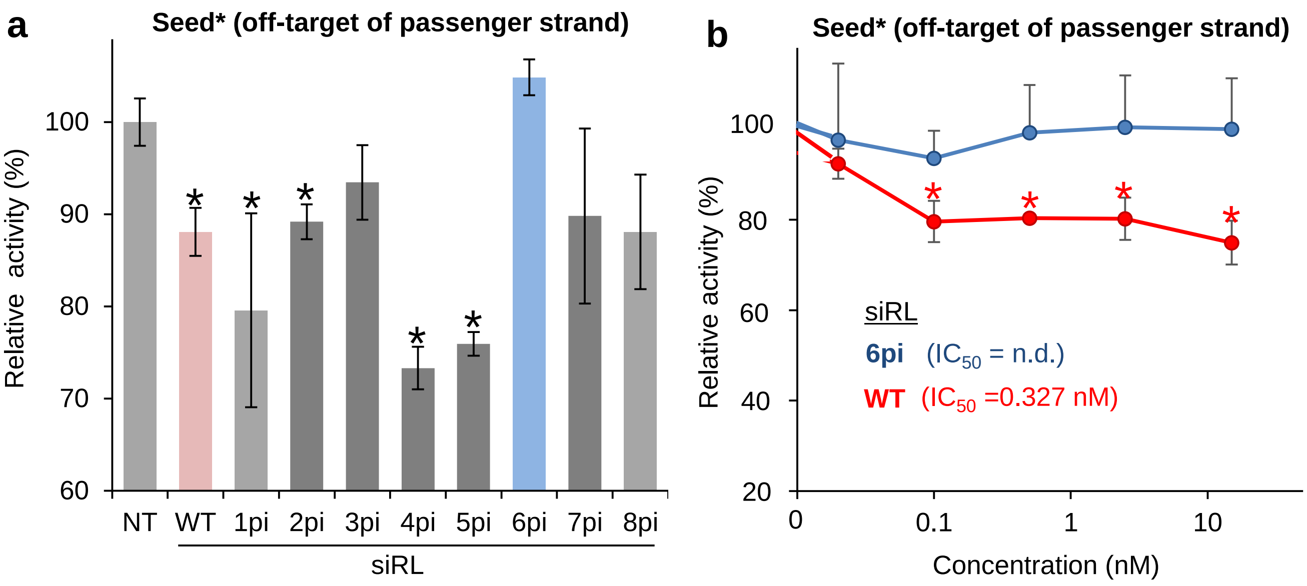

Supplementary Figure 7. Inhibition of passenger strand mediated off-target repression by introducing 6pi in guide strand. (a) The seed-mediated off-target repression by passenger strand of siRNAs was estimated by luciferase reporter assay with seed sites of siRL passenger strand after introducing dSpacer substitution (pi) in various position (1-8) of siRL guide strand. Among the dSpacer substitution (pi) showing inhibition of off-target repression mediated by guide strand of siRL (2-7pi; Fig. 2c), 3pi and 6pi also showed the abrogation of passenger-strand mediated off-target repression. However, siRL-3pi showed poor on-target activity (Fig. 2d). (b) Abrogation of the passenger strand mediated off-target repression by 6pi (0% repression) was also confirmed by measuring IC_{50} (n.d.). Asterisk denotes the case where P<0.01 (t-test, n=6); error bars, s.d.

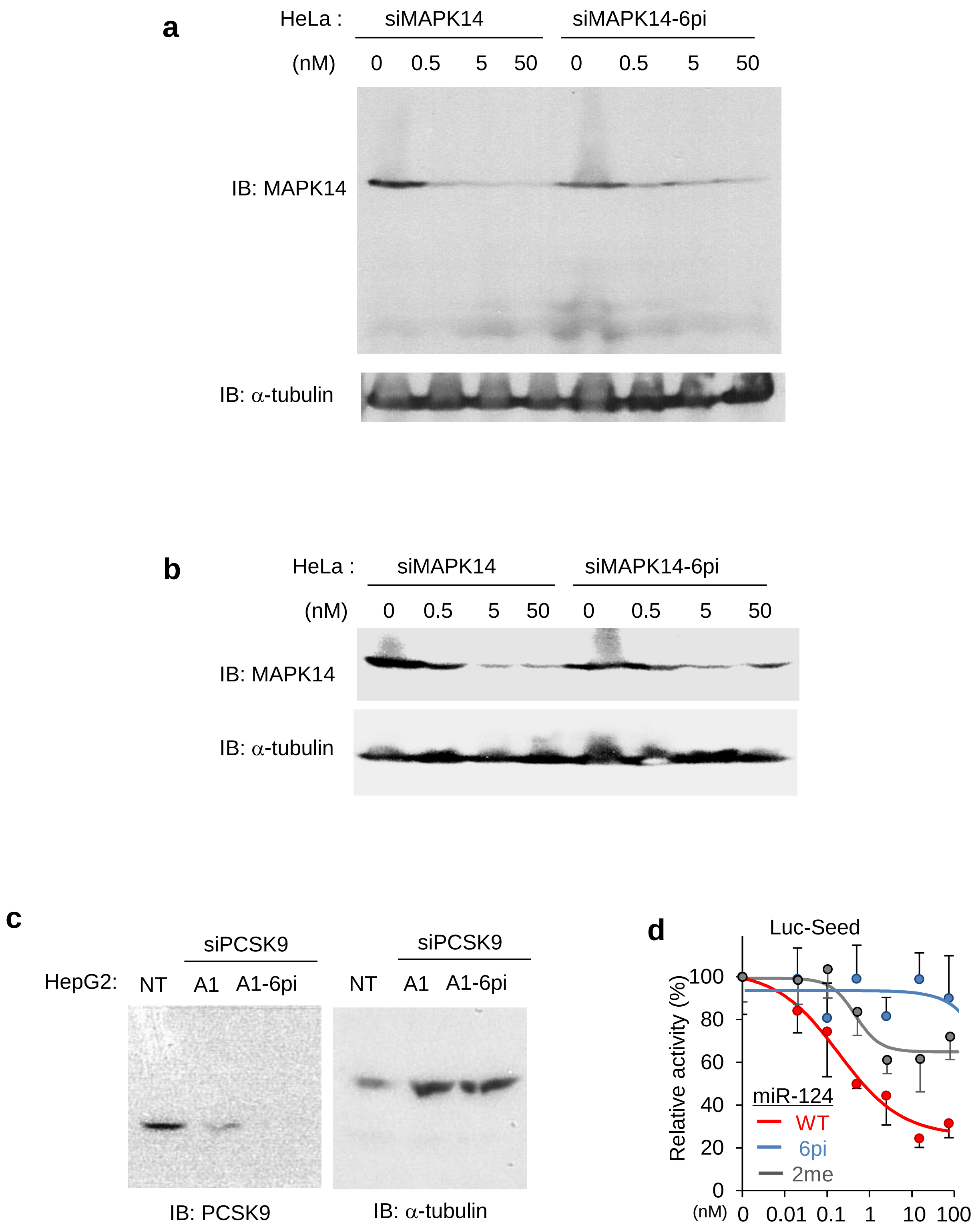
a
HeLa :
siMAPK14
siMAPK14-6pi
(nM)
0
0.5
5
50
0
0.5
5
50
IB: MAPK14
IB: α-tubulin
b
HeLa :
siMAPK14
siMAPK14-6pi
(nM)
0
0.5
5
50
0
0.5
5
50
IB: MAPK14
IB: α-tubulin
c
HepG2:
siPCSK9
NT
A1
A1-6pi
siPCSK9
NT
A1
A1-6pi
IB: PCSK9
IB: α-tubulin
d
Luc-Seed
Relative activity (%)
100
80
60
40
20
0
miR-124
WT
6pi
2me
(nM)
0
0.01
0.1
1
10
100

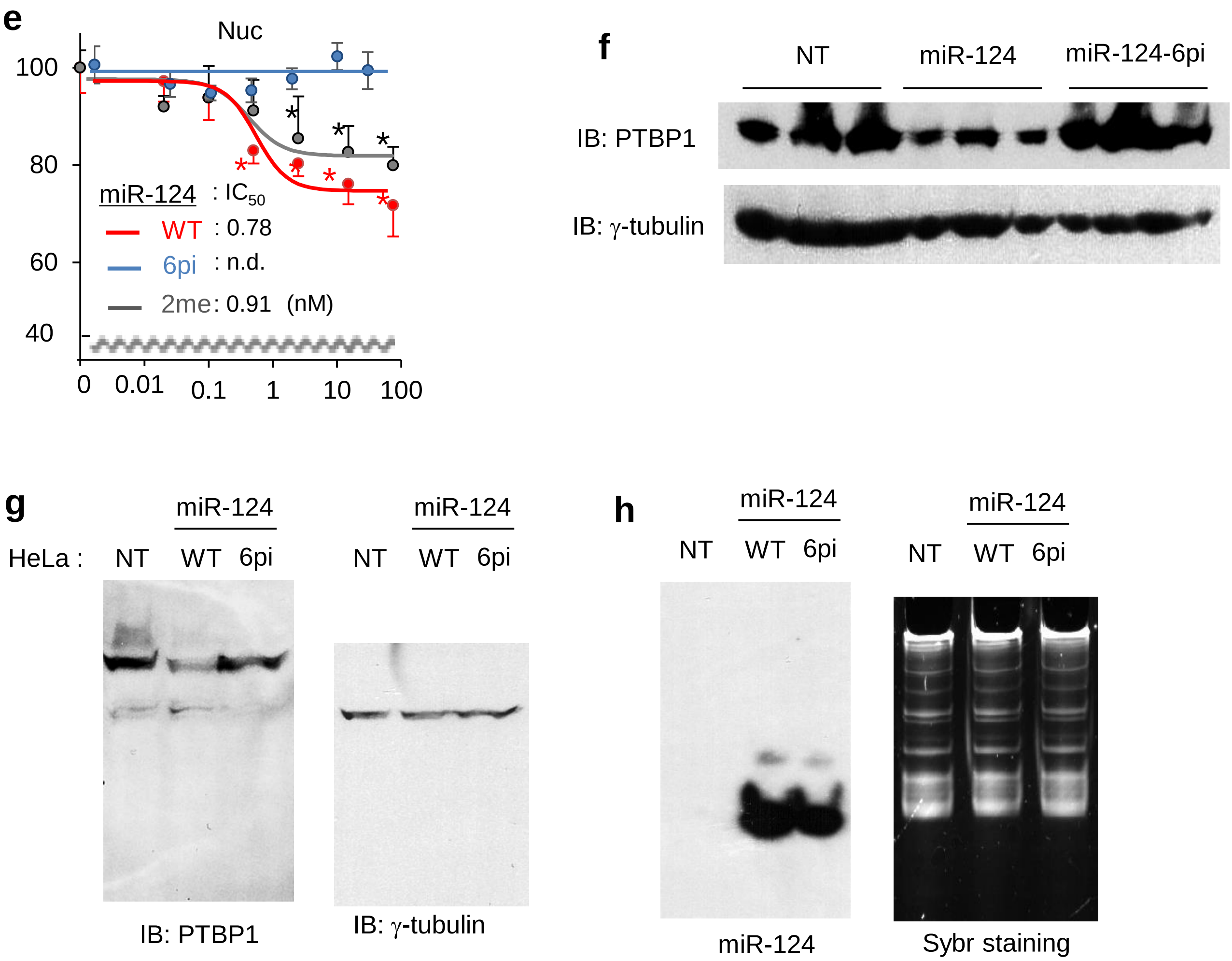

Supplementary Figure 8. Improvement of siRNA specificity by 6pi was confirmed by immunoblot analyses. (a) Conservation of siRNA on-target activity by 6pi was confirmed by performing immunoblot analyses of endogeneous on-target protein, MAPK14, relative to a control (α-tubulin). Different concentrations of siMAPK14 or siMAPK14-6pi were transfected into HeLa for these assays; The same immunoblot results as in Fig. 5c. (b) A result from an experimental replicate as performed in (a). (c) On-target activity of siPCSK9-A1 was examined by immunoblotting PCSK9 proteins in HepG2 after the transfection of 50nM siPCSK9-A1 or siPCSK9-A1-6pi. PCSK9 proteins were dramatically reduced by both A1 and A1-6pi. (d-e) miRNA-like repression mediated by seed (d) or nucleation bulge sites (e) were examined for miR-124 by luciferase reporter assay when 6pi or 2me was applied. IC_{50} values indicate complete derepression by 6pi and remaining repression by 2me. Of note, miR-124 containing 2me in both guide strand (2′OMe in position 2 of guide strand) and passenger strand (2′OMe in positions 1 and 2 of passenger strands) also showed the same results for seed sites (d) as observed in Fig. 5e where 2′OMe in position 2 of guide strand and no modification in passenger strand was used. (f-h) Derepression of a miR-124 target by 6pi was shown by immunoblotting PTBP1, a known miR-124 target. Synthesized NT, miR-124, or miR-124-6pi (75nM) was transfected into HeLa in triplicate for this assay (f). An additional replicate result was represented in (g) with northern blotting results (h) confirming that there is no difference between transfected miR-124 and miR-124-6pi level in the cells. Sybr staining indicates total RNAs loaded for the analysis (h).

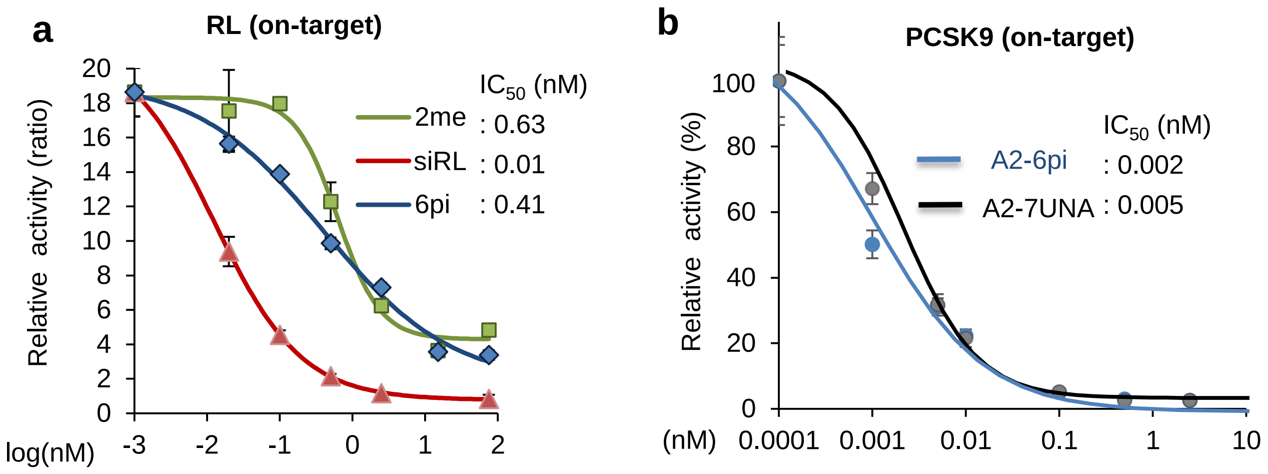

Supplementary Figure 9. dSpacer pivot substitution conserves on-target activity more efficiently than other conventional modifications for siRNAs. (a) On-target activity of siRNA containing 6pi or 2′-OMe (2me, in position 2) was examined for siRL by estimating IC_{50} based on the results from luciferase reporter assays. 6pi showed better on-target activity than 2me. (b) Same analysis as performed in (a) except for comparing 6pi with UNA (7UNA, UNA in position 7) applied to siPCSK9-A2. Of note, the guide strand of A2 is without 2′-OMe modification. Both 2′-OMe and UNA showed less efficient on-target activity than 6pi.

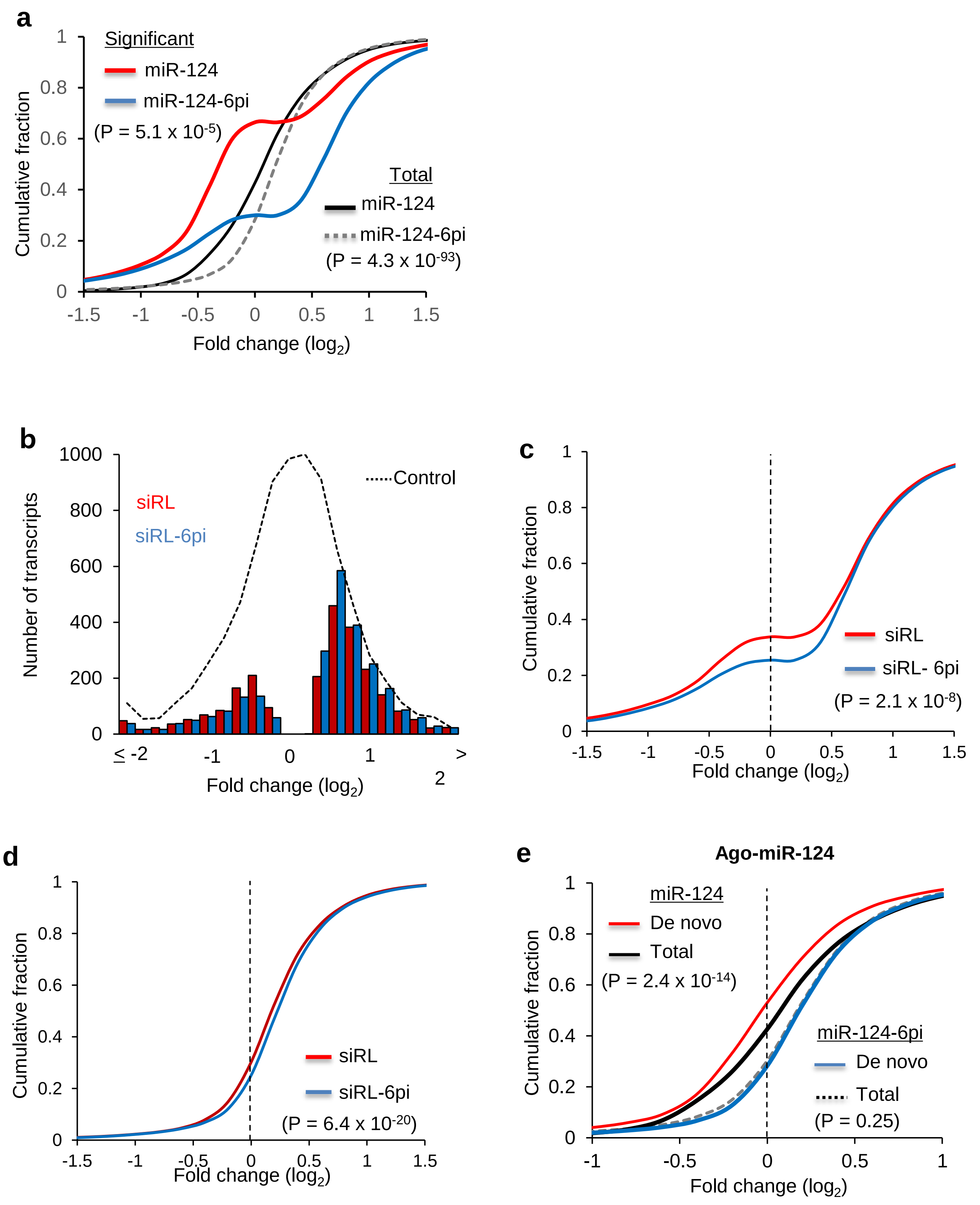

a
Significant
miR-124
miR-124-6pi
(P = 5.1 x 10-5)
Total
miR-124
miR-124-6pi
(P = 4.3 x 10-93)
Cumulative fraction
Fold change (log2)
b
Control
siRL
siRL-6pi
Number of transcripts
Fold change (log2)
c
siRL
siRL- 6pi
(P = 2.1 x 10-8)
Cumulative fraction
Fold change (log2)
d
siRL
siRL-6pi
(P = 6.4 x 10-20)
Cumulative fraction
Fold change (log2)
e
Ago-miR-124
miR-124
De novo
Total
(P = 2.4 x 10-14)
miR-124-6pi
De novo
Total
(P = 0.25)
Cumulative fraction
Fold change (log2)

Supplementary Figure 10. Transcriptome-wide assessment of miRNA-like off-target effects in the presence of dSpacer pivot substitution. (a) Cumulative distributions of significant miR-124 dependent (red line) or miR-124-6pi dependant transcripts (blue line), identified by RNA-Seq analyses in HeLa (significant calls in Cuffdiff), where the corresponding miRNA was transfected. Comparisons between miR-124 and miR-124-6pi showed that 6pi induced significant derepression (as shown by P-value from KS-test). Similar result was also observed by comparing total transcripts from miR-124 (black line) with those from miR-124-6pi (dotted line). (b) Same distribution analysis performed in Fig. 6a (bottom panel), except for comparing siRL with siRL-6pi. 6pi applied to siRL showed transcriptome-wide derepression of off-target effects in RNA-Seq analyses. (c) Cumulative fraction analyses of significant siRL (red line) and siRL-6pi dependent transcripts (blue line) using the same method performed in (a). (d) Same analysis in C, except for comparing to total transcripts as performed in (a). (e) Cumulative distribution analyses of the transcripts bound by Ago-miR-124 ('De novo': de novo Ago-miR-124 clusters identified by Ago HITS-CLIP) were performed in conjunction with transcriptome profiles under miR-124 (red) or miR-124-6pi expression (blue). The analyses were compared to the distribution of all expressed transcripts (total). The cumulative distribution from miR-124-6pi showed no difference with total, implicating that 6pi-induced derepression was brought by preventing Ago complex from binding target sites (mainly through seeds).

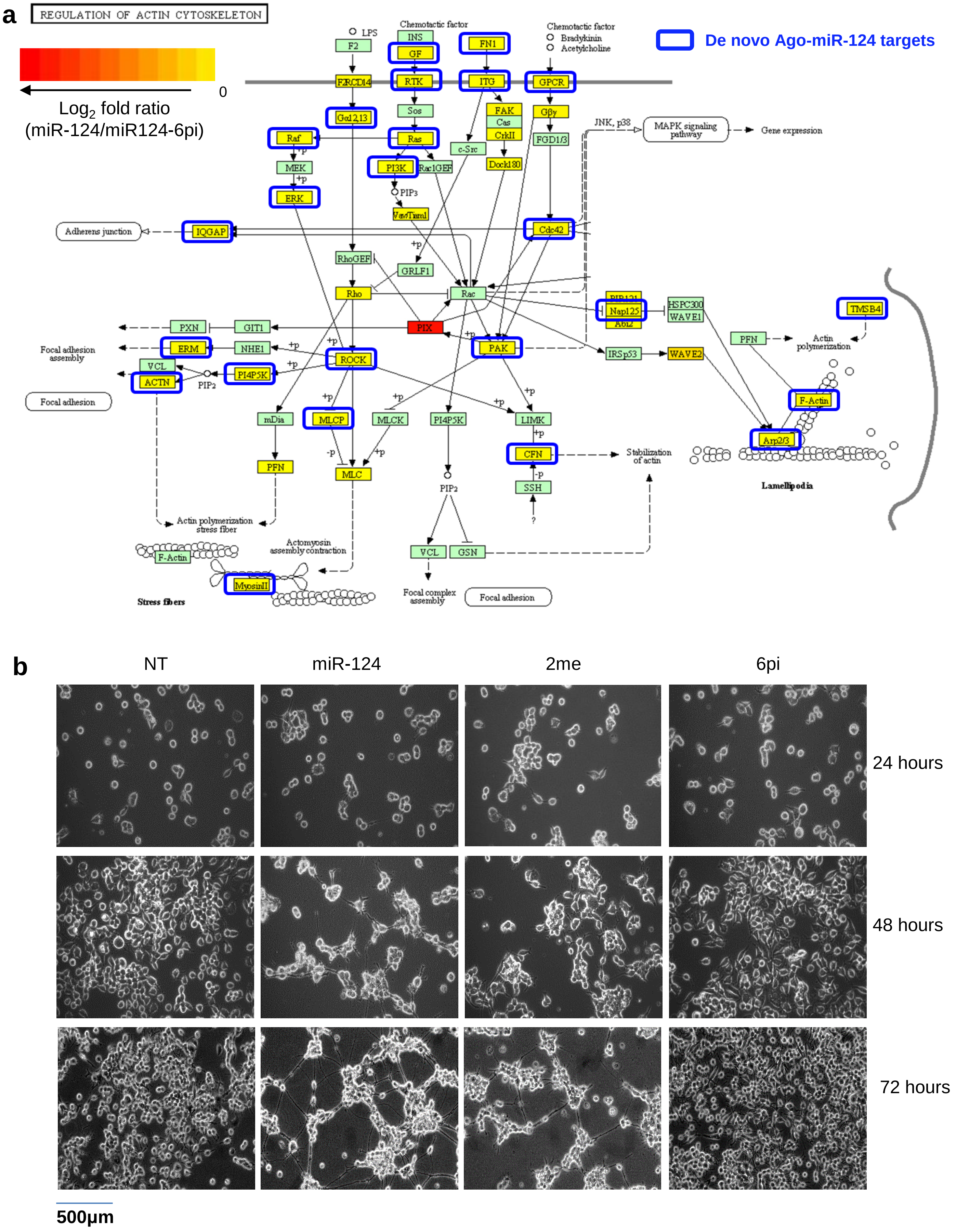
a
REGULATION OF ACTIN CYTOSKELETON
De novo Ago-miR-124 targets
0
Log_2 fold ratio
(miR-124/miR124-6pi)
Chemotactic factor
LPS
F2
INS
GF
FN1
Bradykinin
Acetylcholine
F2RCD14
RTK
ITG
GPCR
Gα12,13
Sos
FAK
Cas
CrkII
Gβγ
Raf
Ras
c-Src
FGD1/3
JNK, p38
MAPK signaling pathway
Gene expression
MEK
PI3K
Rac1GEF
Dock180
ERK
PIP3
VavTiam1
Adherens junction
IQGAP
Cdc42
RhoGEF
GRLF1
Rho
Rac
PIR121
Nap125
Abi2
HSPC300
WAVE1
TMSB4
PXN
GIT1
PIX
PFN
Actin polymerization
Focal adhesion assembly
ERM
NHE1
PAK
IRSp53
WAVE2
VCL
ROCK
ACTN
PIP2
PI4P5K
F-Actin
Focal adhesion
mDia
MLCP
MLCK
PI4P5K
LIMK
Arp2/3
CFN
Stabilization of actin
PFN
MLC
SSH
Lamellipodia
Actin polymerization stress fiber
Actomyosin assembly contraction
VCL
GSN
F-Actin
MyosinII
Stress fibers
Focal complex assembly
Focal adhesion
b
NT
miR-124
2me
6pi
24 hours
48 hours
72 hours
500µm

Supplementary Figure 11. Effect of dSpacer pivot substitution on miR-124 induced neurite outgrowth comparing with 2'-OMe (a) The biological pathway regulating actin cytoskeleton (adopted from KEGG (http://www.genome.jp/kegg/)) was analyzed by integrating miR-124-6pi induced derepression results and Ago-miR-124 bindings. Most of genes, which function at cascades of actin cytoskeleton pathways, were identified as 6pi dependent transcripts (based on RNA-Seq results) and also as direct targets of Ago-miR-124 (based on Ago HITS-CLIP results[9]), implicating that miR-124-6pi may lose regulatory function in this pathway. Colors indicate $\log_2$ fold ratio obtained from RNA-Seq analysis (miR-124 vs. miR-124-6pi in HeLa); green is for genes that were not affected. Direct miR-124 targets containing *de novo* miR-124 clusters identified by Ago HITS-CLIP are indicated by blue rectangle. Of note, actin cytoskeleton pathway is known to be related with neurite outgrowth phenotype during neuronal differentiation, also reported to be regulated by miR-124[11]. (b) Repression of miR-124 induced neurite outgrowth in N2a cells by dSpacer pivot substitution, shown by images at different time points after the transfection. miR-124-6pi lost its function in inducing neurite outgrowth. However, miR-124 containing 2'-OMe (miR-124-2me) still enabled to induce neurosphere-like structure and differentiation, which is similar with what shown by unmodified miR-124 (time laps images, Supplementary Movies 1-3).

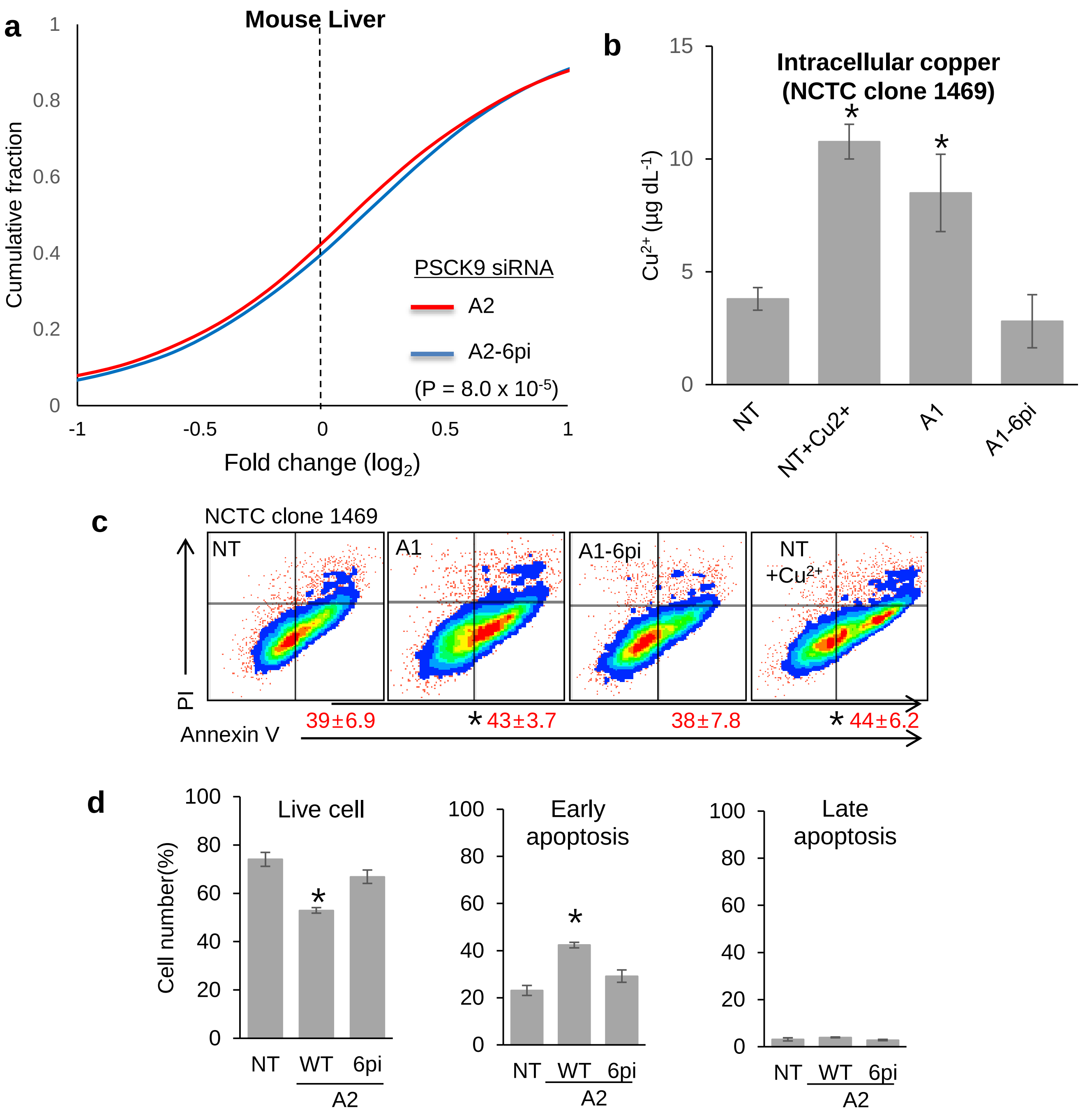

Supplementary Figure 12. Identification of off-target phenotypes of PCSK9 siRNAs and rescue by 6pi in mouse. (a) A cumulative fraction analysis of transcripts from RNA-Seq analysis performed in mouse liver tissue, where delivery of A2 or A2-6pi was confirmed (Fig. 7a). The putative siPCSK9-A2 off-targets in mouse liver were significantly derepressed by siPCSK9-A2-6pi *in vivo* (P-value indicated, KS-test). (b) siPCSK9-A1 expression significantly increases intracellular copper in mouse liver cell line, NCTC clone 1469, but A1-6pi showed no change relative to a control ('NT', non-targeting control siRNA); 'Cu^{2+}', treatment of 32µM $CuSO_4$. (c) Cell death assays of NCTC clone 1469 cells under NT, siPCSK9-A1 and siPCSK9-A1-6pi expression, measured by FACS analysis with PI and Annexin V staining; indicated as percentage with standard deviation on the representative FACS results. (d) Results of cell death assay performed in Fig. 7f are also represented as bar graphs. It shows significant increase in early apoptosis and decrease in live cells, induced by siPCSK9-A2 and not by siPCSK9-A2-6pi in NCTC clone 1469. In all analyses, error bar indicates standard deviation and asterisk denotes $P<0.01$, t-test (n = 3).

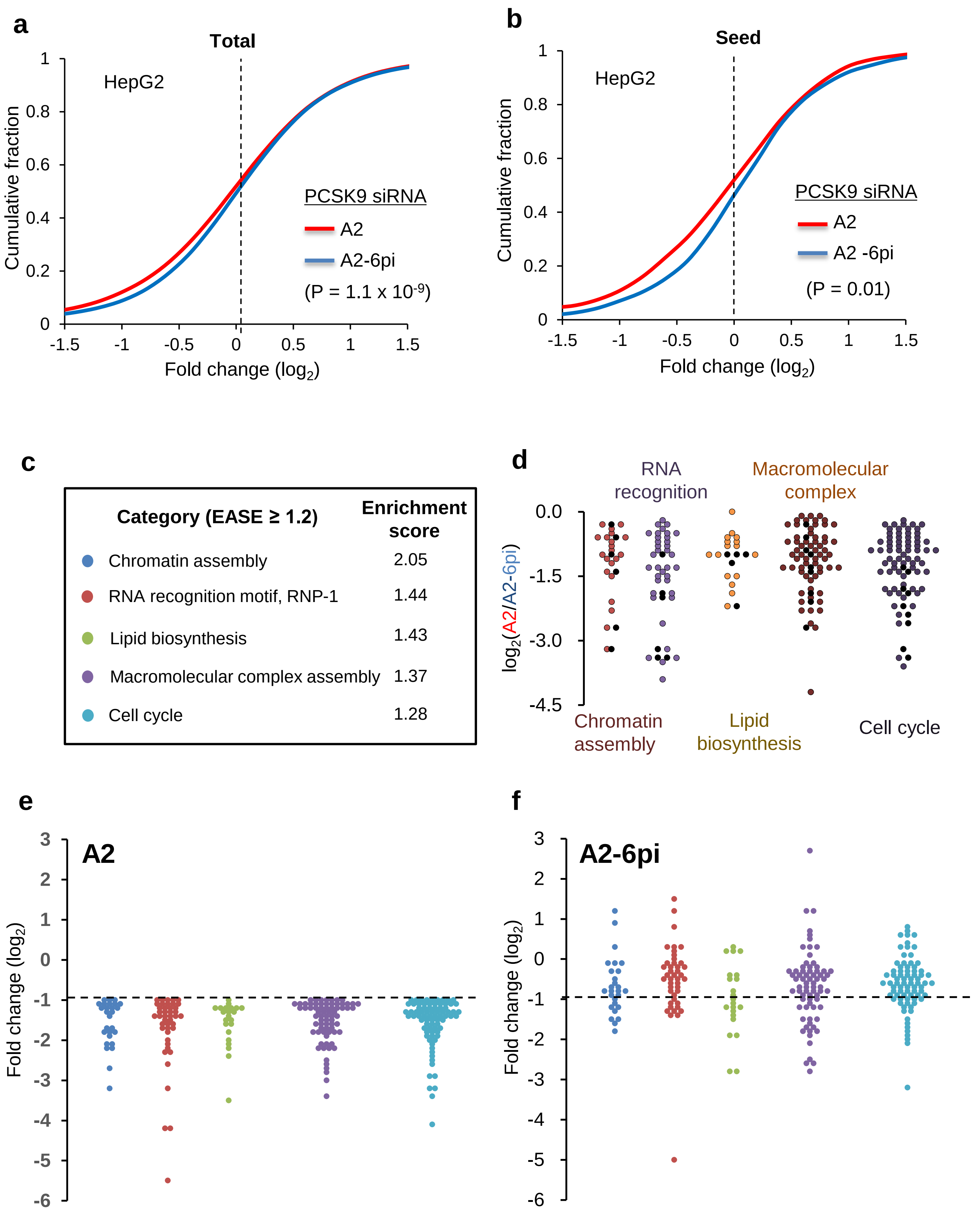

a
Total
HepG2
Cumulative fraction
PCSK9 siRNA
A2
A2-6pi
(P = 1.1 x 10^-9)
Fold change (log2)
b
Seed
HepG2
Cumulative fraction
PCSK9 siRNA
A2
A2 -6pi
(P = 0.01)
Fold change (log2)
c
Category (EASE ≥ 1.2)
Enrichment score
Chromatin assembly 2.05
RNA recognition motif, RNP-1 1.44
Lipid biosynthesis 1.43
Macromolecular complex assembly 1.37
Cell cycle 1.28
d
RNA recognition
Macromolecular complex
log2(A2/A2-6pi)
Chromatin assembly
Lipid biosynthesis
Cell cycle
e
A2
Fold change (log2)
f
A2-6pi
Fold change (log2)

Supplementary Figure 13. Determination of the putative off-target phenotypes of PCSK9 siRNAs in human liver cells. (a) Derepression of siPCSK9-A2 off-targets by 6pi in human liver cell line, HepG2. A cumulative fraction analysis was performed as in Supplementary Figure 12a, except for comparing siPCSK9-A2 with siPCSK9-A2-6pi in HepG2. (b) Same cumulative fraction analysis in (a), except for considering the putative direct off-targets of A2, which contain predicted seed sites (7mer). (c) Gene ontology (GO) analysis of the putative A2 off-target transcripts, which showed A2 dependant repression ($\log_2$(A2/NT) < -2) and also derepressed by siPCSK9-A2-6pi ($\log_2$(A2-6pi/A2) > 0.25). GO analysis was performed by using DAVID (with default options, http://david.abcc.ncifcrf.gov/) and for clustering functional annotations in the putative siPCSK9-A2 off-target transcripts under the background of all expressed transcripts in HepG2. GO categories in the results of this analysis were further selected by using 1.2 as threshold in EASE score. Ultimately, 5 functional GO categories were selected and indicated in the box. (d) The degree of siPCSK9-A2 off-target repression comparing with siPCSK9-6pi ($\log_2$(A2/A2-6pi)) was analyzed for 5 functional clusters of GO categories (selected in (c)). (e-f) In detail analysis performed as in (d), but only for transcripts that were repressed by siPCSK9-A2 expression ($\log_2$(A2) < -1). Results are separately shown as fold changes, which were induced by the expression of siPCSK9-A2 (e) vs. siPCSK9-A2-6pi (f) in HepG2. Of note, "cell cycle" is one of the most dramatically altered GO category in terms of fold derepression ($\log_2$(A2/A2-6pi)).

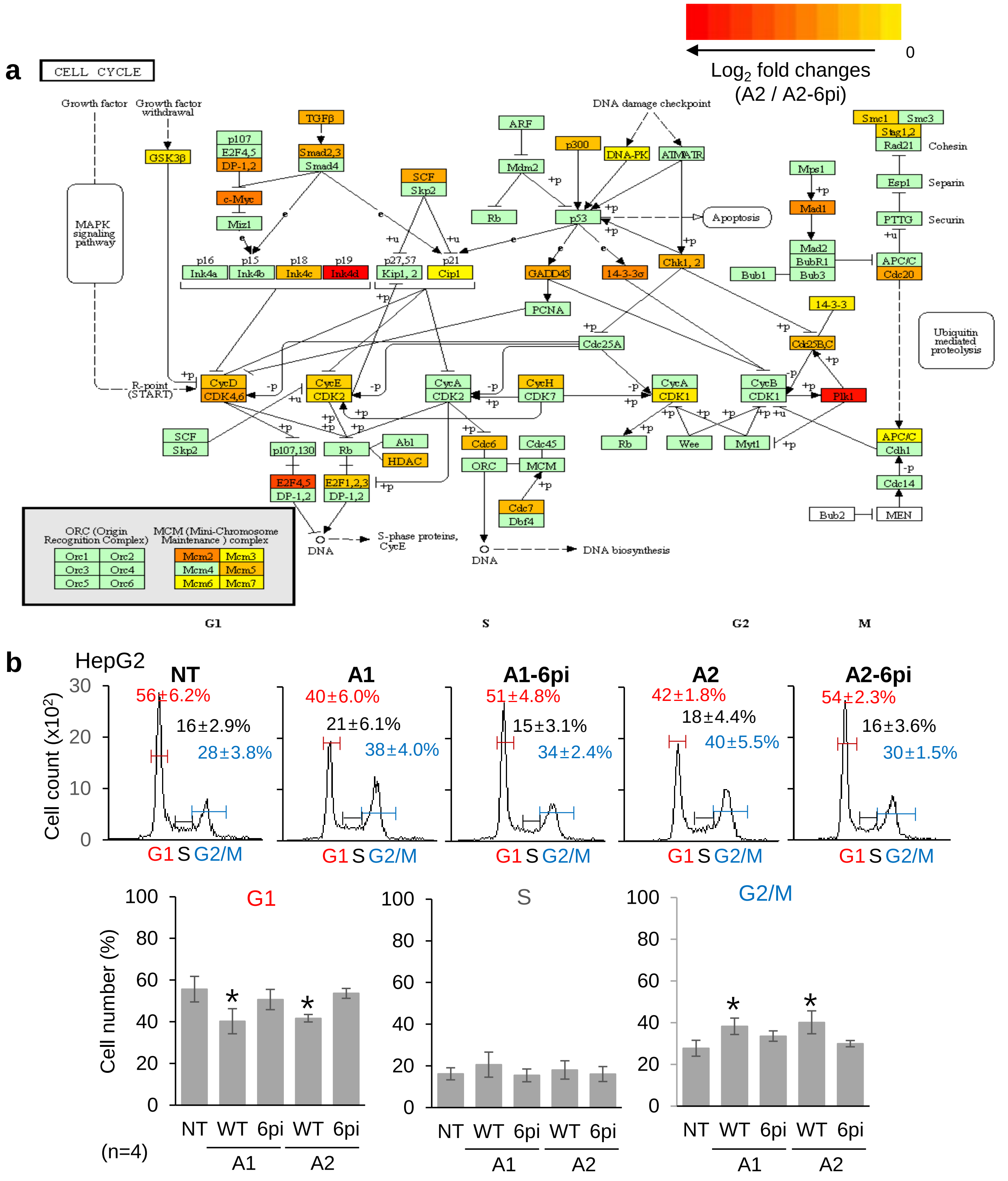

Supplementary Figure 14. Cell cycle defect induced by PCSK9 siRNAs and its recue by dSpacer pivot substitution in HepG2. (a) Same pathway analysis as performed in Supplementary Figure 11a, except only using $\log_2$ fold changes caused by siPCSK9-A2 and siPCSK9-A2-6pi expression, measured by RNA-Seq analysis in HepG2. About a half of genes in the pathway of cell cycle regulation were repressed as siPCSK9-A2 off-targets, implicating that there might be a defect in cell cycle regulation in siPCSK9-A2 expressed HepG2. (b) Cell cycle analysis of NT, siPCSK9-A1, siPCSK9-A1-6pi, siPCKS9-A2 and siPCSK9-A2-6pi transfected HepG2 cells by FACS analysis with propidium iodide (PI) staining. Defect in cell cycle was induced by the expression of siPCKS9-A1 or siPCSK9-A2, but not by siPPCSK9-A1-6pi or siPCSK9-A2-6pi in HepG2; indicated as percentage of cell with s.d. (upper panel, n=3) on the representative FACS results. The results are also represented as bar graphs (lower panel); error bars, s.d. ; Asterisk denotes $P<0.01$, t-test.

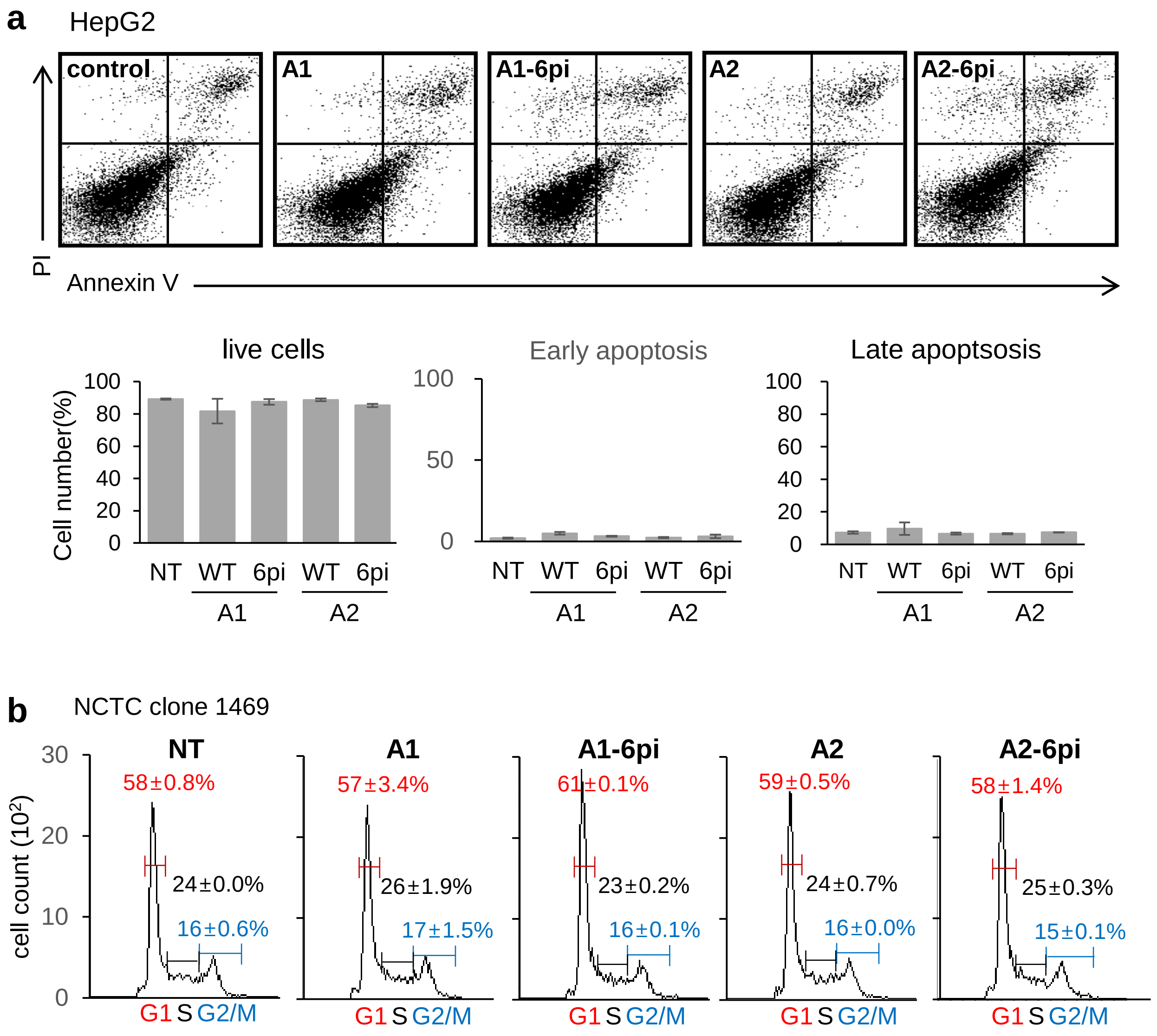

Supplementary Figure 15. Species-specific off-target phenotypes observed in PCSK9 siRNAs. (a) induction of apoptotic cell death by PCSK9 siRNAs (siPCSK9-A1 and siPCSK9-A2; Supplementary Figure 12c-d) is a mouse specific off-target phenotype, confirmed by observing no changes of cell death in HepG2 even under the expression of siPCSK9-A1 or siPCSK9-A2. Cell death analysis was performed as in Supplementary Figure 12c-d. (b) The defect of cell cycle induced by PCSK9 siRNAs is a human specific off-target phenotype, confirmed by observing no changes of cell cycles in NCTC clone 1469 under the expression of siPCSK9-A1 or siPCSK9-A2. Cell cycle analysis was performed as in Supplementary Figure 14b.

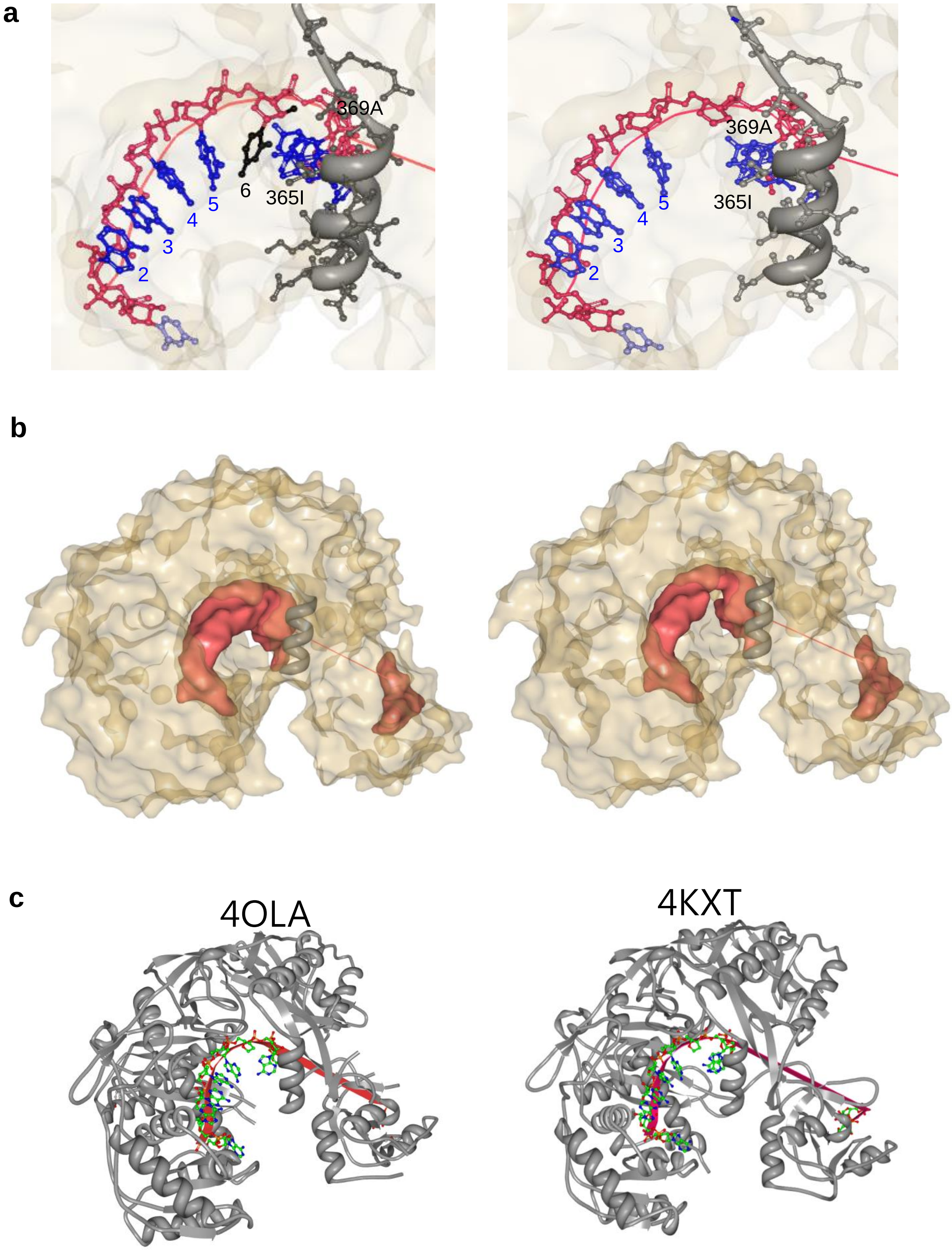
a
369A
6
365I
5
4
3
2
369A
5
365I
4
3
2
b
c
4OLA
4KXT

d

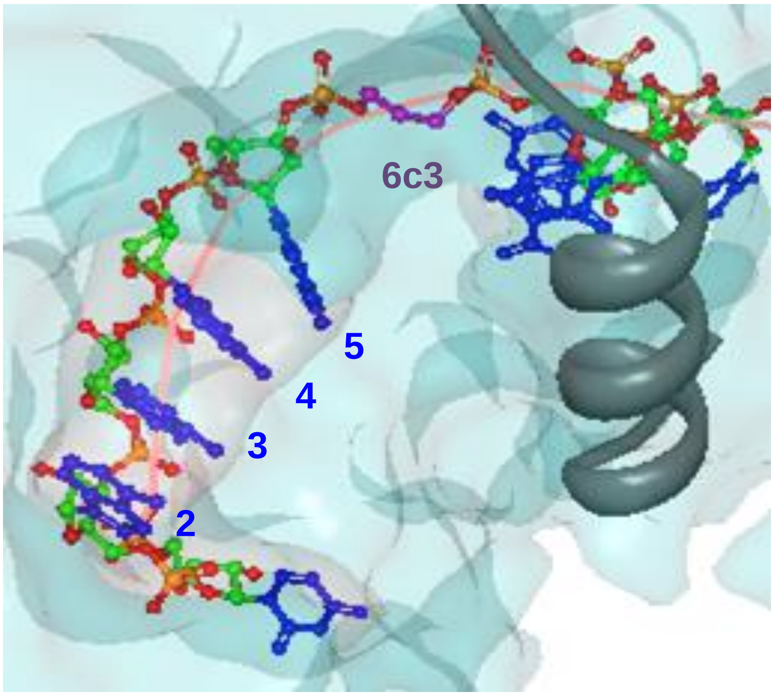

Supplementary Figure 16. Structural analysis and modeling of human Ago-miRNA complex with dSpacer pivot substitution. (a) Detail view of human Ago-miRNA structure (4F3T[8], left panel) and its Ago-miRNA-6pi model (right panel); Ago, grey; miRNA, red; base, blue; pivot base, black. Alpha-helix from Ago, which generates a kink (positon 6-7) in miRNA, is indicated with impeding amino acid residues (I365 and A369). (b) Surface structure of human Ago-miRNA (4F3T[8], left panel) and its model containing 6pi (right panel); Ago in brown and miRNA in red. Of note, 6pi reduced the bulkiness of surface in pivot which closely contacts with alpha-helix of human Ago producing the kink. (c) Ago-miRNA-6pi models derived from 4OLA[6] (left panel) and 4KXT[7] (right panel). (d) Modeling of Ago-miRNA cotaining C3 spacer (Fig. 8c, upper panel) substitution for pivot (6c3); Ago, faint cyan; miRNA, faint red; base, blue; C3 spacer, purple.

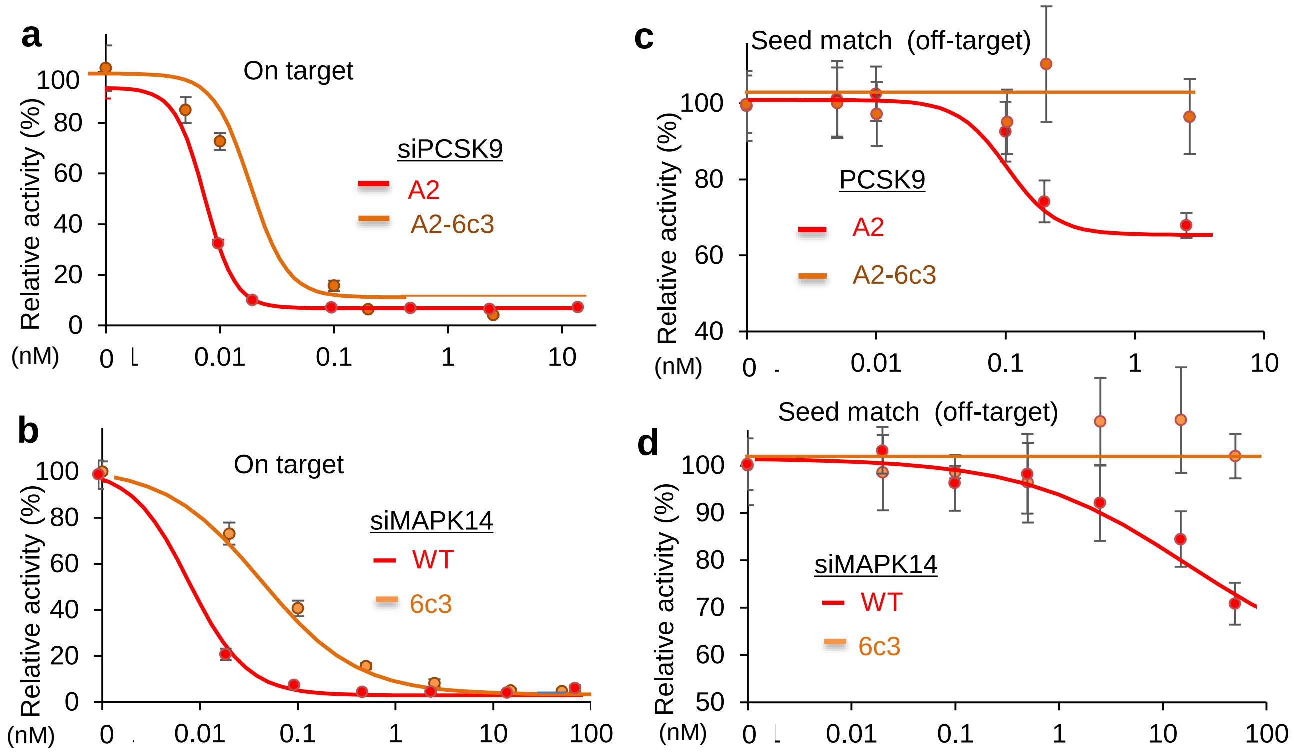

Supplementary Figure 17. C3 spacer substitution for pivot improves target specificity of siRNAs. (a) C3 spacer (Fig. 8c, upper panel) substitution for pivot (6c3) was applied to PCSK9 siRNA (siPCSK9-A2) and its effect on repressing perfectly matched on-target sites was analyzed by estimating IC_{50} and I_{max} using luciferase reporter assays (I_{max}=100%, IC_{50}[6c3] = 0.04 nM, IC_{50}[WT] = 0.008 nM). (b) Same analysis performed in (a) except for 6c3 applied to siMAPK14 (I_{max} = 100%, IC_{50}[6c3] = 0.07nM, IC_{50}[WT] = 0.007 nM). (c) Effect of 6c3 on seed-mediated off-target repression was measured for siPCSK9-A2 by luciferase reporter assays (IC_{50}[6c3] = n.d., IC_{50}[WT] = 0.08 nM). (d) Same analysis performed in (c) except for siMAPK14 (IC_{50}[6c3] = n.d., IC_{50}[WT] = 8.12 nM).

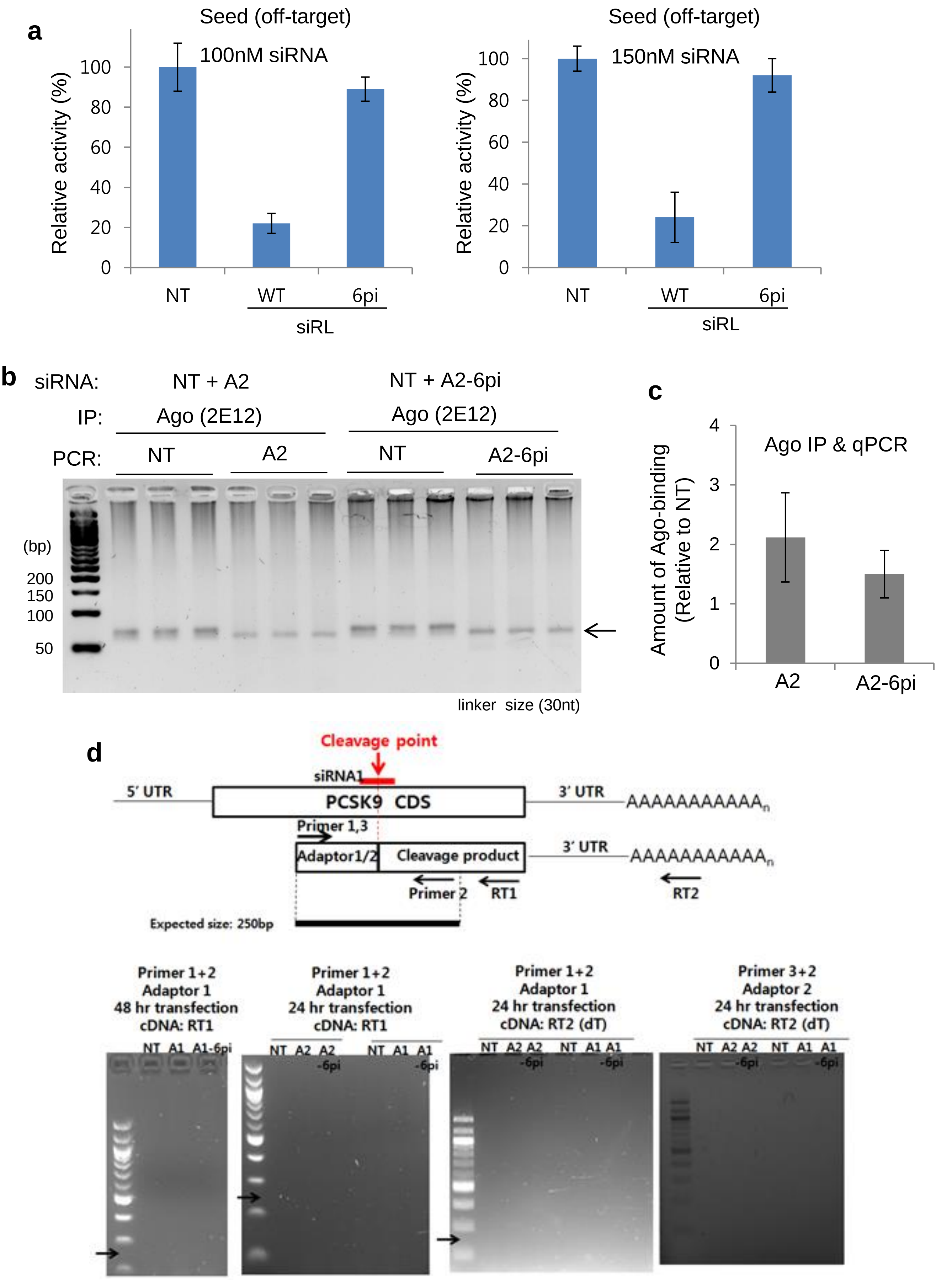
a
Seed (off-target)
100nM siRNA
Relative activity (%)
0
20
40
60
80
100
NT
WT
6pi
siRL
Seed (off-target)
150nM siRNA
Relative activity (%)
0
20
40
60
80
100
NT
WT
6pi
siRL
b
siRNA:
NT + A2
NT + A2-6pi
IP:
Ago (2E12)
Ago (2E12)
PCR:
NT
A2
NT
A2-6pi
(bp)
200
150
100
50
linker size (30nt)
c
Ago IP & qPCR
Amount of Ago-binding (Relative to NT)
0
1
2
3
4
A2
A2-6pi
d
Cleavage point
siRNA1
5′ UTR
PCSK9 CDS
3′ UTR
AAAAAAAAAAA
Primer 1,3
Adaptor1/2
Cleavage product
3′ UTR
AAAAAAAAAAA
Primer 2
RT1
RT2
Expected size: 250bp
Primer 1+2
Adaptor 1
48 hr transfection
cDNA: RT1
NT A1 A1-6pi
Primer 1+2
Adaptor 1
24 hr transfection
cDNA: RT1
NT A2 A2 -6pi
NT A1 A1 -6pi
Primer 1+2
Adaptor 1
24 hr transfection
cDNA: RT2 (dT)
NT A2 A2 -6pi
NT A1 A1 -6pi
Primer 3+2
Adaptor 2
24 hr transfection
cDNA: RT2 (dT)
NT A2 A2 -6pi
NT A1 A1 -6pi

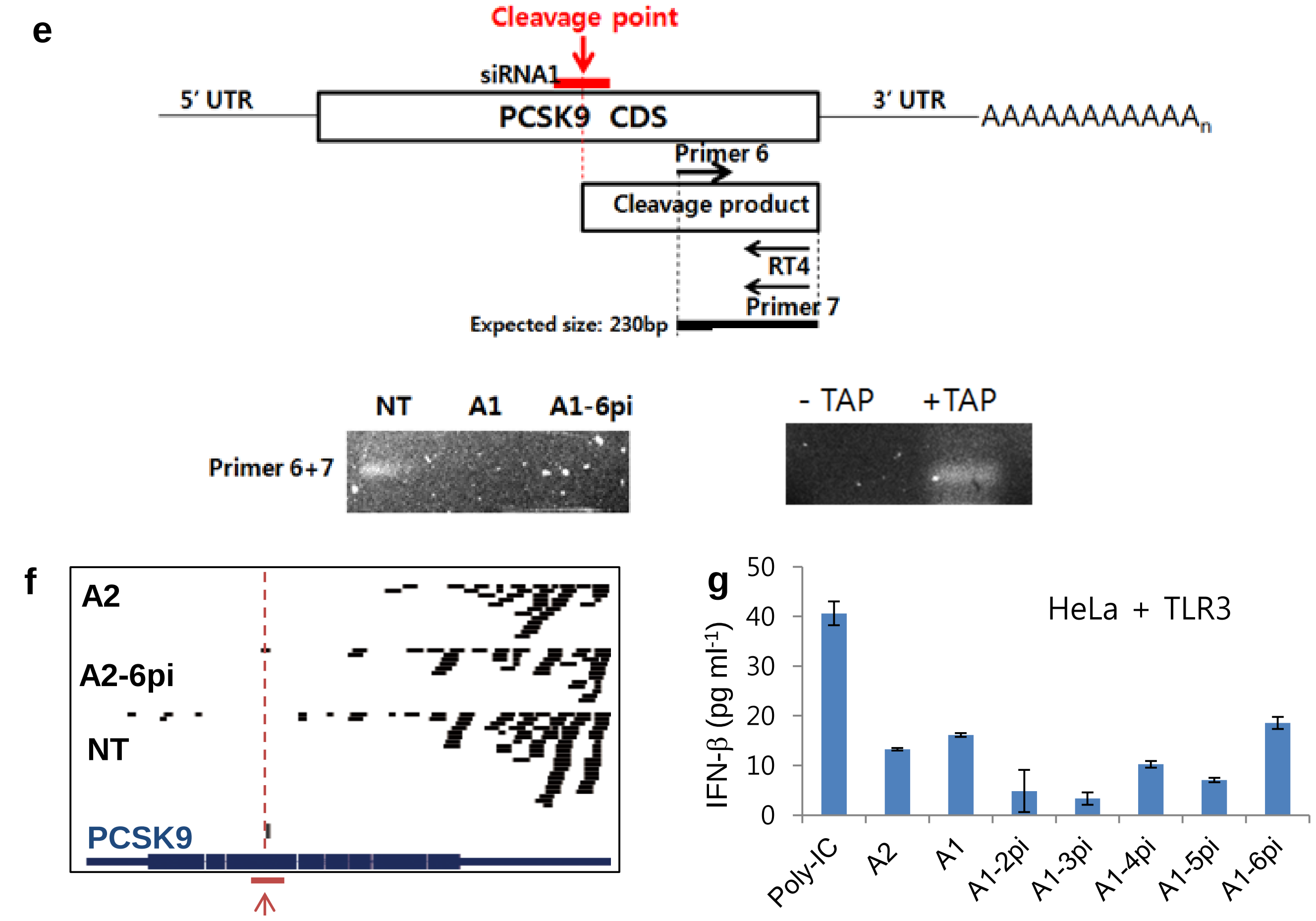

Supplementary Figure 18. Effect of siRNA-6pi on seed-mediated off-target repression, Ago loading, cleavage of on-target and TLR3-mediated innate immune response. (a) off-target repression mediated by seed was completed abolished by 6pi even in the case of using high concentration of siRNA; 100nM (left panel) or 150nM (right panel) of siRNAs (siRL, siRL-6pi, NT). Of note, 150nM is the highest concentration that can be applied to HeLa cells without significant toxicity. (b-c) Amount of siRNAs complexed with Ago was not changed by 6pi. To examine the effect of 6pi on Ago loading, Ago protein complex was immunoprecipitated by Ago2 specific antibody (2E12) in HeLa cell lysates after siPCSK9-A2 or siPCSK9-A2-6pi was cotransfected with NT. Amount of siRNAs in Ago complex was examined by RT-PCR amplification (b) and was further quantitated as relative ratio to co-transfected NT by qPCR (c). Significant difference was not observed. (d) To see whether 6pi affects the cleavage activity of Ago for an on-target *in vivo*, RACE-PCR was performed to detect a cleavage product of PCSK9 mRNA by siPCSK9-A1 or siPCSK9-A2 in HepG2 cells. As represented (upper panel), various combinations of different linkers and primers were attempted, but there was no expected cleavage product observed (lower panel); arrows for indicating expected size. (e) To confirm the negative results we observed in (d), a primer set that could amplify both intact and cleaved products was used for RT-PCR as indicated (upper panel). Although expected size of product was amplified in the control (NT), there was no product amplified in the presence of A1 or A1-6pi (lower left panel). A control experiment, which amplified CXCR4 mRNA by 5'-end RACE-PCR, confirmed that RACE-PCR method we used had no problem (lower right panel); (-)TAP was used as negative control. (f) Sequencing reads from RNA-seq experiments performed in siPCSK9-A2, siPCSK9-A2-6pi or NT delivered mouse liver (Fig. 7a-d) were visualized for PCSK9 mRNA; Red bar for location of on-target site, red arrow and dotted line for expected cleavage site by siPCSK9-A2. Reduced numbers of sequencing reads in siPCSK9-A2 and siPCSK9-A2-6pi relative to NT were consistent with the results observed by qPCR (Fig. 7b). Of note, we did observed one read of which 3' end was exactly matched to the cleavage site point siPCSK9-A2-6pi. Although we didn't observed the read supporting the cleavage in A2, it might be because of reduced amount of PCSK9 mRNA. (g) siRNA mediated innate immune response was examined by measuring IFN-β production in TLR3 transfected HeLa cells using ELISA assay. siPCSK9-A1 elicited more IFN-β production than A2 (the same as A1 except containing several 2'-OMe modifications to inhibit innate immune response); the consistent results with previous reports[13]. Poly-IC was used as positive control. Of note, we did not observed any significant change of IFN-β production by siPCSK9-A1-6pi relative to siPCSK9-A1.

a

Gene/siRNA	Cell line (source)	siRNA sequence (guide strand)	siRNA_sequence (passenger strand)	Seed (2-8, 7mer)	Nuc
MAPK14-1	HeLa (Jackson 2003)	AACCGCAGUUCUCUGUAGG-dTdT	CCUACAGAGAACUGCGGUU-dTdT	CTGCGGT	TGGCGGT
MAPK14-2	HeLa (Jackson 2003)	UCCAACAGACCAAUCACAU-dTdT	AUGUGAUUGGUCUGUUGGA-dTdT	CTGTTGG	TGGTTGG
MAPK14-3	HeLa (Jackson 2003)	UACUUUAGACCUCGGAGAA-dTdT	UUCUCCGAGGUCUAAAGUA-dTdT	CTAAAGT	TAAAAGT
MAPK14-4	HeLa (Jackson 2003)	UUUAGGUCCCUGUGAAUUA-dTdT	UAAUUCACAGGGACCUAAA-dTdT	GACCTAA	ACCCTAA
MAPK14-5	HeLa (Jackson 2003)	CAUAAGGAUCGGCCACUGG-dTdT	CCAGUGGCCGAUCCUUAUG-dTdT	TCCTTAT	CCCTTAT
MAPK14-6	HeLa (Jackson 2003)	GUACUGAGCAAAGUAGGCA-dTdT	UGCCUACUUUGCUCAGUAC-dTdT	CTCAGTA	TCCAGTA
MAPK14-7	HeLa (Jackson 2003)	GUGGCACAAAGCUGAUGAC-dTdT	GUCAUCAGCUUUGUGCCAC-dTdT	TGTGCCA	GTTGCCA
APOB-Hs1	HUH7 (Burchard 2009)	AAUUUUUCAAAGUUCCAAU-dTdT	AUUGGAACUUUGAAAAAUU-dTdT	GAAAAAT	AAAAAAT
APOB-Hs2	HUH7 (Burchard 2009)	UAGUUAUUCAGGAAGUCUA-dTdT	UAGACUUCCUGAAUAACUA-dTdT	AATAACT	ATTAACT
APOB-Hs3	HUH7 (Burchard 2009)	AUUUCAGGAAUUGUUAAAG-dTdT	CUUUAACAAUUCCUGAAAU-dTdT	CCTGAAA	CTTGAAA
APOB-Hs4	HUH7 (Burchard 2009)	UUGGUAUUCAGUGUGAUGA-dTdT	UCAUCACACUGAAUACCAA-dTdT	AATACCA	ATTACCA
PIK3CA-2629	HeLa (Jackson 2006b)	GGCCAAAGAUUCAAAGCCA-dTdT	UGGCUUUGAAUCUUUGGCC-dTdT	CTTTGGC	TTTTGGC
MPHOSPH1-2692	HeLa (Jackson 2006b)	GGUGAUCACUCUCCUUCAU-dTdT	AUGAAGGAGAGUGAUCACC-dTdT	TGATCAC	GAATCAC
PRKCE-1295	HeLa (Jackson 2006b)	CUCAAAUAGGUCGUCCUCA-dTdT	UGAGGACGACCUAUUUGAG-dTdT	TATTTGA	ATTTTGA
VHL-2651	HeLa (Jackson 2006b)	CUUACCCUUUUGGGUUCUG-dTdT	CAGAACCCAAAAGGGUAAG-dTdT	AGGGTAA	GGGGTAA
VHL-2652	HeLa (Jackson 2006b)	CACACCCUGCCUAUUUCCU-dTdT	AGGAAAUAGGCAGGGUGUG-dTdT	AGGGTGT	GGGGTGT
SOS1-1582	HeLa (Jackson 2006b)	CAGAAACCUGGUGGUCAAU-dTdT	AUUGACCACCAGGUUUCUG-dTdT	GGTTTCT	GTTTTCT
PIK3CB6338	HeLa (Jackson 2006a)	UUGACCAUGAUGUUGUCAC-dTdT	GUGACAACAUCAUGGUCAA-dTdT	ATGGTCA	TGGGTCA
PLK1319	HeLa (Jackson 2006a)	UUGUAGAGGAUGAGGCGUG-dTdT	CACGCCUCAUCCUCUACAA-dTdT	CTCTACA	TCCTACA
PIK3CB6340	HeLa (Jackson 2006a)	AUAGGAUUCAUAUUAGGAG-dTdT	CUCCUAAUAUGAAUCCUAU-dTdT	AATCCTA	ATTCCTA
PLK772	HeLa (Jackson 2006a)	UGAUCCGGAGGUAGGUCUC-dTdT	GAGACCUACCUCCGGAUCA-dTdT	CCGGATC	CGGGATC
IGF1R-1	HeLa (Jackson 2003)	CUCGGUAAUGACCGUGAGC-dTdT	GCUCACGGUCAUUACCGAG-dTdT	TTACCGA	TAACCGA
IGF1R-2	HeLa (Jackson 2003)	CCGAGUAAUGUUCCUCAGG-dTdT	CCUGAGGAACAUUACUCGG-dTdT	TTACTCG	TAACTCG
IGF1R-3	HeLa (Jackson 2003)	GAGGUAACAGAGGUCAGCA-dTdT	UGCUGACCUCUGUUACCUC-dTdT	GTTACCT	TTTACCT
IGF1R-4	HeLa (Jackson 2003)	AGCUACACAGGCCGUGUCG-dTdT	CGACACGGCCUGUGUAGCU-dTdT	GTGTAGC	TGGTAGC
IGF1R-5	HeLa (Jackson 2003)	UCCGGCCAUCUGAAUCAUC-dTdT	GAUGAUUCAGAUGGCCGGA-dTdT	TGGCCGG	GGGCCGG
IGF1R-6	HeLa (Jackson 2003)	GUCCCACAGUUGCUGCAAG-dTdT	CUUGCAGCAACUGUGGGAC-dTdT	TGTGGGA	GTTGGGA
IGF1R-7	HeLa (Jackson 2003)	GCCGCGGAUGACCGUGAGG-dTdT	CCUCACGGUCAUCCGCGGC-dTdT	TCCGCGG	CCCGCGG
IGF1R-8	HeLa (Jackson 2003)	GAAGAUGACCAGGGCGUAG-dTdT	CUACGCCCUGGUCAUCUUC-dTdT	TCATCTT	CAATCTT
IGF1R-9	HeLa (Jackson 2003)	AAGCCCAAUAUCCUUGAGA-dTdT	UCUCAAGGAUAUUGGGCUU-dTdT	TTGGGCT	TGGGGCT
IGF1R-12	HeLa (Jackson 2003)	GGUAACAGAGGUCAGCAUU-dTdT	AAUGCUGACCUCUGUUACC-dTdT	CTGTTAC	TGGTTAC
IGF1R-13	HeLa (Jackson 2003)	CAGCAAGUACUCGGUAAUGNN	CAUUACCGAGUACUUGCUGCU	ACTTGCT	CTTTGCT
IGF1R-14	HeLa (Jackson 2003)	CACUCGGAACAGCAGCAAGNN	CUUGCUGCUGUUCCGAGUGGC	TCCGAGT	CCCGAGT
IGF1R-15	HeLa (Jackson 2003)	UCGAGGCCAGCCACUCGGANN	UCCGAGUGGCUGGCCUCGAGA	GGCCTCG	GCCCTCG
IGF1R-16	HeLa (Jackson 2003)	CUCCGAGGCUCUCGAGGCCNN	GGCCUCGAGAGCCUCGGAGAC	CCTCGGA	CTTCGGA

b

Gene/siRNA	Conc (nM)	Time (Hour)	Cell line (source)	siRNA sequence (guide strand)	siRNA_sequence (passenger strand)	Seed (2-8, 7mer)	Nuc
MAPK14-1	0.08	24	HeLa (Jackson 2003)	AACCGCAGUUCUCUGUAGG-dTdT	CCUACAGAGAACUGCGGUU-dTdT	CTGCGGT	TGGCGGT
MAPK14-1	0.12	24	HeLa (Jackson 2003)	AACCGCAGUUCUCUGUAGG-dTdT	CCUACAGAGAACUGCGGUU-dTdT	CTGCGGT	TGGCGGT
MAPK14-1	4	24	HeLa (Jackson 2003)	AACCGCAGUUCUCUGUAGG-dTdT	CCUACAGAGAACUGCGGUU-dTdT	CTGCGGT	TGGCGGT
MAPK14-1	20	24	HeLa (Jackson 2003)	AACCGCAGUUCUCUGUAGG-dTdT	CCUACAGAGAACUGCGGUU-dTdT	CTGCGGT	TGGCGGT
MAPK14-1	100	24	HeLa (Jackson 2003)	AACCGCAGUUCUCUGUAGG-dTdT	CCUACAGAGAACUGCGGUU-dTdT	CTGCGGT	TGGCGGT
MAPK14-1	100	1	HeLa (Jackson 2003)	AACCGCAGUUCUCUGUAGG-dTdT	CCUACAGAGAACUGCGGUU-dTdT	CTGCGGT	TGGCGGT
MAPK14-1	100	2	HeLa (Jackson 2003)	AACCGCAGUUCUCUGUAGG-dTdT	CCUACAGAGAACUGCGGUU-dTdT	CTGCGGT	TGGCGGT
MAPK14-1	100	4	HeLa (Jackson 2003)	AACCGCAGUUCUCUGUAGG-dTdT	CCUACAGAGAACUGCGGUU-dTdT	CTGCGGT	TGGCGGT
MAPK14-1	100	6	HeLa (Jackson 2003)	AACCGCAGUUCUCUGUAGG-dTdT	CCUACAGAGAACUGCGGUU-dTdT	CTGCGGT	TGGCGGT
MAPK14-1	100	12	HeLa (Jackson 2003)	AACCGCAGUUCUCUGUAGG-dTdT	CCUACAGAGAACUGCGGUU-dTdT	CTGCGGT	TGGCGGT
MAPK14-1	100	24	HeLa (Jackson 2003)	AACCGCAGUUCUCUGUAGG-dTdT	CCUACAGAGAACUGCGGUU-dTdT	CTGCGGT	TGGCGGT
MAPK14-1	100	48	HeLa (Jackson 2003)	AACCGCAGUUCUCUGUAGG-dTdT	CCUACAGAGAACUGCGGUU-dTdT	CTGCGGT	TGGCGGT
MAPK14-1	100	72	HeLa (Jackson 2003)	AACCGCAGUUCUCUGUAGG-dTdT	CCUACAGAGAACUGCGGUU-dTdT	CTGCGGT	TGGCGGT
MAPK14-1	100	96	HeLa (Jackson 2003)	AACCGCAGUUCUCUGUAGG-dTdT	CCUACAGAGAACUGCGGUU-dTdT	CTGCGGT	TGGCGGT
MAPK14-2	100	24	HeLa (Jackson 2003)	UCCAACAGACCAAUCACAU-dTdT	AUGUGAUUGGUCUGUUGGA-dTdT	CTGTTGG	TGGTTGG
MAPK14-3	100	24	HeLa (Jackson 2003)	UACUUUAGACCUCGGAGAA-dTdT	UUCUCCGAGGUCUAAAGUA-dTdT	CTAAAGT	TAAAAGT
MAPK14-4	100	24	HeLa (Jackson 2003)	UUUAGGUCCCUGUGAAUUA-dTdT	UAAUUCACAGGGACCUAAA-dTdT	GACCTAA	ACCCTAA
MAPK14-5	100	24	HeLa (Jackson 2003)	CAUAAGGAUCGGCCACUGG-dTdT	CCAGUGGCCGAUCCUUAUG-dTdT	TCCTTAT	CCCTTAT
MAPK14-6	100	24	HeLa (Jackson 2003)	GUACUGAGCAAAGUAGGCA-dTdT	UGCCUACUUUGCUCAGUAC-dTdT	CTCAGTA	TCCAGTA
MAPK14-7	100	24	HeLa (Jackson 2003)	GUGGCACAAAGCUGAUGAC-dTdT	GUCAUCAGCUUUGUGCCAC-dTdT	TGTGCCA	GTTGCCA
MAPK14-8	100	24	HeLa (Jackson 2003)	GAGUUCCCGUGAAAAGGCC-dTdT	GGCCUUUUCACGGGAACUC-dTdT	GGGAACT	GGGAACT

Supplementary Table 1. **Microarray data used in meta-analyses to examine miRNA-like off-target effects.** (a) Information of published microarray data[1-4], which measured fold changes of transcripts where siRNA was expressed (35 different siRNAs). For performing cumulative distribution analysis (Fig. 1c, left panel and Supplementary Figure 1a), normalized $\log_2$ fold ratios were selected from the compiled microarray data[5]. (b) The list of microarray data used in the heat map analysis (Fig. 1c, right panel), focusing on siRNAs targeting the same gene (MAPK14) in the compiled normalized set[5]. 14 different combinations of siRNA concentration and time (harvest time after siRNA transfection) for a specific siRNA sequence (MAPK14-1) were analyzed using published microarray data[2]. In addition, 8 different siRNA sequences (MAPK14-1 to -8) were also analyzed for the same condition (100nM siRNA, 24 hours).

a

GEO Accession	siRNA name	siRNA sequence (guide strand)	siRNA_sequence (passenger strand)	Seed (2-8, 7mer)	Nuc
GSM134317	PIK3CA-2629	GGCCAAAGAUUCAAAGCCA	UGGCUUUGAAUCUUUGGCC	CTTTGGC	TTTTGGC
GSM134319<br>GSM134330	MPHOSPH1-2692	GGUGAUCACUCUCCUUCAU	AUGAAGGAGAGUGAUCACC	TGATCAC	GAATCAC
GSM134321<br>GSM134332	SOS1-1582	CAGAAACCUGGUGGUCAAU	AUUGACCACCAGGUUUCUG	GGTTTCT	GTTTTCT
GSM134323<br>GSM134334	PRKCE-1295	CUCAAAUAGGUCGUCCUCA	UGAGGACGACCUAUUUGAG	TATTTGA	ATTTTGA
GSM134325<br>GSM134336	VHL-2651	CUUACCCUUUUGGGUUCUG	CAGAACCCAAAAGGGUAAG	AGGGTAA	GGGGTAA
GSM134327<br>GSM134338	VHL-2652	CACACCCUGCCUAUUUCCU	AGGAAAUAGGCAGGGUGUG	AGGGTGT	GGGGTGT
GSM134522	MAPK14-193	AACCGCAGUUCUCUGUAGG	CCUACAGAGAACUGCGGUU	CTGCGGT	TGGCGGT

b

GEO Accession	siRNA name	siRNA sequence (guide strand)	siRNA_sequence (passenger Strand)	Seed (2-8, 7mer)	Nuc
GSM134318<br>GSM134329	PIK3CA-2629	GGCCAAAGAUUCAAAGCCA	UGGCUUUGAAUCUUUGGCC	CTTTGGC	TTTTGGC
GSM134320<br>GSM134331	MPHOSPH1-2692	GGUGAUCACUCUCCUUCAU	AUGAAGGAGAGUGAUCACC	TGATCAC	GAATCAC
GSM134322<br>GSM134333	SOS1-1582	CAGAAACCUGGUGGUCAAU	AUUGACCACCAGGUUUCUG	GGTTTCT	GTTTTCT
GSM134324<br>GSM134335	PRKCE-1295	CUCAAAUAGGUCGUCCUCA	UGAGGACGACCUAUUUGAG	TATTTGA	ATTTTGA
GSM134326<br>GSM134337	VHL-2651	CUUACCCUUUUGGGUUCUG	CAGAACCCAAAAGGGUAAG	AGGGTAA	GGGGTAA
GSM134328<br>GSM134339	VHL-2652	CACACCCUGCCUAUUUCCU	AGGAAAUAGGCAGGGUGUG	AGGGTGT	GGGGTGT
GSM134538	MAPK14-193	AACCGCAGUUCUCUGUAGG	CCUACAGAGAACUGCGGUU	CTGCGGT	TGGCGGT

Supplementary Table 2. Microarray data used for the analysis of miRNA-like off-target effects in 2′-OMe modified siRNAs. (a-b) The list of microarray data downloaded from GEO database (http://www.ncbi.nlm.nih.gov/geo/) to be used for the comparison of off-target effects between unmodified (a) and 2′-OMe modified siRNAs (b), based on the previous publication[3]. Normalized $\log_{10}$ fold ratios in downloaded files from GEO were converted into $\log_2$, and used for cumulative distribution analysis (Fig. 1d and Supplementary Figure 1b-c). The nucleotides in position 2, modified to 2′-OMe were indicated in red (b).

a

Exp	Sample Name	siRNA	Source	RNA-Seq Library	Total reads	Accepted hits (RefSeq)
1	HeLa-A	NT	HeLa	PolyA	16,235,171	9,270,618
2	HeLa-B	NT	HeLa	PolyA	15,119,643	6,449,078
4	HeLa-C	NT	HeLa	NSR	12,652,033	4,187,328
2	HeLa-Cont	Cel-miR-67	HeLa	PolyA	14,983,064	3,589,030
1	HeLa-124-A	miR-124	HeLa	PolyA	13,001,596	6,955,535
4	HeLa-124-B	miR-124	HeLa	NSR	13,559,456	6,933,732
1	HeLa-124-6pi-A	miR-124-6pi	HeLa	PolyA	17,095,211	13,546,866
4	HeLa-124-6pi-B	miR-124-6pi	HeLa	NSR	11,499,203	5,603,331
1	HeLa-RL-A	siRL	HeLa	PolyA	18,690,809	14,686,104
4	HeLa-RL-B	siRL	HeLa	NSR	15,005,454	7,379,360
1	HeLa-RL-6pi-A	siRL-6pi	HeLa	PolyA	19,815,739	15,058,973
4	HeLa-RL-6pi-B	siRL-6pi	HeLa	NSR	11,551,512	5,152,714
2	HeLa-RL-2me	siRL-2me	HeLa	PolyA	21,145,907	7,429,700

b

Exp	Sample Name	siRNA	Source	RNA-Seq Library	Total reads	Accepted hits (RefSeq)
6	Mouse-NT	NT	Mouse liver	PolyA	23,735,236	10,778,918
5	Mouse-A2-A	A2	Mouse liver	NSR	27,348,301	15,380,671
6	Mouse-A2-B	A2	Mouse liver	PolyA	25,839,994	9,295,863
5	Mouse-A2-6pi-A	A2-6pi	Mouse liver	NSR	26,258,233	12,731,871
6	Mouse-A2-6pi-B	A2-6pi	Mouse liver	PolyA	22,699,508	8,569,293

c

Exp	Sample Name	siRNA	Source	RNA-Seq Library	Total reads	Accepted hits (RefSeq)
3	HepG2-A	NT	HepG2	NSR	7,002,338	1,605,079
3	HepG2-B	NT	HepG2	NSR	4,124,133	1,515,705
3	HepG2-C	NT	HepG2	NSR	5,342,310	1,668,301
6	HepG2-D	NT	HepG2	PolyA	23,205,020	12,208,560
3	HepG2-A2-A	A2	HepG2	NSR	5,760,763	1,438,473
3	HepG2-A2-B	A2	HepG2	NSR	9,079,300	2,660,904
5	HepG2-A2-C	A2	HepG2	NSR	15,100,068	4,234,490
5	HepG2-A2-D	A2	HepG2	NSR	19,371,434	3,068,877
6	HepG2-A2-E	A2	HepG2	PolyA	22,136,988	10,765,579
3	HepG2-A2-6pi-A	A2-6pi	HepG2	NSR	10,477,579	3,444,336
3	HepG2-A2-6pi-B	A2-6pi	HepG2	NSR	11,226,958	3,529,221
6	HepG2-A2-6pi-C	A2-6pi	HepG2	PolyA	21,984,628	11,730,699

Supplementary Table 3. RNA-Seq . (a-c) RNA-Seq libraries were constructed by performing NSR or PolyA method (See details in on-line methods section). For this, total RNAs were extracted from HeLa cells (a), mouse liver tissues (b), and HepG2 cells (c) after different siRNAs were delivered. There were six trials of RNA-Seq experiments in parallel as indicated in "Exp" (1, PolyA; 2, PolyA; 3, NSR; 4, NSR; 5, NSR; 6, PolyA). "Total reads" indicates the number of reads from high-throughput sequencing. "Accepted hits (RefSeq)" indicates the number of mapped reads by running TopHat2 under supply of RefSeq gene annotation. Raw data (fastq files) can be dowloaded from the link in our project website (http://ago.korea.ac.kr/6pi/) or Sequence Read Archive (SRP047107, SRP047205, SRP047269).

RefSeq	Log_2 (A2/NT)	log_2 (A2-6pi/A2)	GO function	Gene description
NM_008491	-5.221	0.486		lipocalin 2
**NM_013602**	**-3.931**	**0.772**	**metal ion binding**	**metallothionein 1**
**NM_008630**	**-3.688**	**0.448**	**metal ion binding**	**metallothionein 2**
NM_001271419	-2.362	1.149		lymphocyte antigen 6 complex, locus A (Ly6a), transcript variant 5
NM_001164038	-2.212	0.411	embryonic development	lymphocyte antigen 6 complex, locus E
NM_008183	-2.165	1.399		predicted gene 6665; glutathione S-transferase, mu 2
NM_019467	-2.064	1.498	metal ion binding	allograft inflammatory factor 1
NM_029465	-2.063	0.980		C-type lectin domain family 4, member g
NM_009344	-1.975	0.563	apoptosis	pleckstrin homology-like domain, family A, member 1
NM_007870	-1.795	0.706	apoptosis, metal ion binding	deoxyribonuclease 1-like 3
NM_007812	-1.742	1.543	metal ion binding	cytochrome P450, family 2, subfamily a, polypeptide 21, pseudo gene; cytochrome P450, family 2, subfamily a, polypeptide 5
NM_009690	-1.738	0.234	apoptosis	CD5 antigen-like
NM_011016	-1.715	0.326	Inflammatory reponse	orosomucoid 2
NM_029803	-1.705	1.012		interferon, alpha-inducible protein 27 like 2A
NM_010378	-1.660	0.022	Inflammatory reponse , nucleotide binding	histocompatibility 2, class II antigen A, alpha; histocompatibility 2, class II antigen E alpha
NM_010359	-1.587	2.109		glutathione S-transferase, mu 3
NM_016917	-1.568	0.412	metal ion binding	solute carrier family 40 (iron-regulated transporter), member 1
NM_007822	-1.495	1.094	metal ion binding	cytochrome P450, family 4, subfamily a, polypeptide 14
NM_007824	-1.484	0.616	Lipid metabolism , metal ion binding	cytochrome P450, family 7, subfamily a, polypeptide 1
NM_029796	-1.483	0.211		leucine-rich alpha-2-glycoprotein 1
NM_153133	-1.477	0.188	lipid metabolism	retinol dehydrogenase 9
NM_026381	-1.417	0.814	apoptosis	shisa homolog 5 (Xenopus laevis)
NM_013640	-1.315	0.485		proteasome (prosome, macropain) subunit, beta type 10
NM_026784	-1.300	0.249	Lipid metabolism, nucleotide binding	phosphomevalonate kinase
NM_009040	-1.293	0.298	Metal ion binding	retinol dehydrogenase 16
NM_198095	-1.251	0.296		bone marrow stromal cell antigen 2
NM_026764	-1.178	0.328		glutathione S-transferase, mu 4
NM_008175	-1.131	0.162	embryonic development	granulin

Supplementary Table 4. Putative off-target transcripts of siPCSK9-A2 in mouse liver. The list of putative siPCSK9-A2 off-target transcripts, which showed significant A2 dependent repression (log_2(A2/NT) < 0, significant call in Cuffdiff) but derepressed by siPCSK9-A2-6pi (log_2(A2-6pi/A2) > 0), identified by RNA-Seq analyses in siRNA-delivered mouse liver tissues (confirmed by the results in Fig. 7a). The putative siPCSK9-A2 off-targets were further analyzed by gene ontology (GO), of which terms are represented together as table. The most enriched GO term, 'metal ion binding', is highlighted with red. GO categories in the table were selected by performing clustering of functional annotation using DAVID (with default option, only in GO terms of molecular function and biological process; http://david.abcc.ncifcrf.gov/) within the putative siPCSK9-A2 off-target transcripts under the background of expressed transcripts in mouse liver (all detected transcripts in control, 'NT'). Representing GO terms for each functional clusters are as follows: metal ion binding (GO:0046872), apoptosis (GO:0006915), inflammatory response (GO:0006954), lipid metabolism (GO:0008202), embryonic development (GO:0001701) and nucleotide binding (GO:0000166). MT1 (metallothionein1) and MT2 (metallothionein2), which are known to function in copper metabolism, are highlighted with bold letter.

Supplementary References

1. Burchard, J. et al. MicroRNA-like off-target transcript regulation by siRNAs is species specific. *RNA* **15**, 308-15 (2009).
2. Jackson, A.L. et al. Expression profiling reveals off-target gene regulation by RNAi. *Nat Biotechnol* **21**, 635-7 (2003).
3. Jackson, A.L. et al. Position-specific chemical modification of siRNAs reduces "off-target" transcript silencing. *RNA* **12**, 1197-205 (2006).
4. Jackson, A.L. et al. Widespread siRNA "off-target" transcript silencing mediated by seed region sequence complementarity. *RNA* **12**, 1179-87 (2006).
5. Khan, A.A. et al. Transfection of small RNAs globally perturbs gene regulation by endogenous microRNAs. *Nat Biotechnol* **27**, 549-55 (2009).
6. Schirle, N.T. & MacRae, I.J. The crystal structure of human Argonaute2. *Science* **336**, 1037-40 (2012).
7. Nakanishi, K., Weinberg, D.E., Bartel, D.P. & Patel, D.J. Structure of yeast Argonaute with guide RNA. *Nature* **486**, 368-74 (2012).
8. Elkayam, E. et al. The structure of human argonaute-2 in complex with miR-20a. *Cell* **150**, 100-10 (2012).
9. Chi, S.W., Hannon, G.J. & Darnell, R.B. An alternative mode of microRNA target recognition. *Nat Struct Mol Biol* **19**, 321-7 (2012).
10. Chi, S.W., Zang, J.B., Mele, A. & Darnell, R.B. Argonaute HITS-CLIP decodes microRNA-mRNA interaction maps. *Nature* **460**, 479-86 (2009).
11. Wang, Y. et al. Structure of an argonaute silencing complex with a seed-containing guide DNA and target RNA duplex. *Nature* **456**, 921-6 (2008).
12. Makeyev, E.V., Zhang, J., Carrasco, M.A. & Maniatis, T. The MicroRNA miR-124 promotes neuronal differentiation by triggering brain-specific alternative pre-mRNA splicing. *Mol Cell* **27**, 435-48 (2007).
13. Frank-Kamenetsky, M. et al. Therapeutic RNAi targeting PCSK9 acutely lowers plasma cholesterol in rodents and LDL cholesterol in nonhuman primates. *Proc Natl Acad Sci U S A* **105**, 11915-20 (2008).
